# Characterization of the Adult Head Transcriptome and Identification of Migration and Olfaction Genes in the Oriental Armyworm *Mythimna separate*

**DOI:** 10.1038/s41598-017-02513-6

**Published:** 2017-05-24

**Authors:** Hai-Xu Bian, Hong-Fang Ma, Xi-Xi Zheng, Ming-Hui Peng, Yu-Ping Li, Jun-Fang Su, Huan Wang, Qun Li, Run-Xi Xia, Yan-Qun Liu, Xing-Fu Jiang

**Affiliations:** 1 Insect Resource Center for Engineering and Technology of Liaoning Province, College of Bioscience and Biotechnology, Shenyang Agricultural University, Shenyang, 110866 China; 2 School of Basic Medicine, Guangzhou University of Chinese Medicine, Guangzhou, 510006 China; 3 State Key laboratory for Biology of Plant Diseases and Insect Pest, Institute of Plant Protection, Chinese Academy of Agricultural Sciences, Beijing, 100193 China

## Abstract

The oriental armyworm *Mythimna separate* is an economically important insect with a wide distribution and strong migratory activity. However, knowledge about the molecular mechanisms regulating the physiological and behavioural responses of the oriental armyworm is scarce. In the present study, we took a transcriptomic approach to characterize the gene network in the adult head of *M*. *separate*. The sequencing and *de novo* assembly yielded 63,499 transcripts, which were further assembled into 46,459 unigenes with an N50 of 1,153 bp. In the head transcriptome data, unigenes involved in the ‘signal transduction mechanism’ are the most abundant. In total, 937 signal transduction unigenes were assigned to 22 signalling pathways. The circadian clock, melanin synthesis, and non-receptor protein of olfactory gene families were then identified, and phylogenetic analyses were performed with these *M*. *separate* genes, the model insect *Bombyx* *mori* and other insects. Furthermore, 1,372 simple sequence repeats of 2–6 bp in unit length were identified. The transcriptome data represent a comprehensive molecular resource for the adult head of *M*. *separate*, and these identified genes can be valid targets for further gene function research to address the molecular mechanisms regulating the migratory and olfaction genes of the oriental armyworm.

## Introduction

The oriental armyworm, *Mythimna separate* Walker (Lepidoptera: Noctuidae), is a polyphagous, migratory pest of high economic importance and can attack >300 plant species in nearly 100 families of crops, such as corn (*Zea mays*), sorghum (*Sorghum bicolor*), and rice (*Oryza sativa*). This pest is widely distributed between latitudes 45 °N and 45 °S and between longitudes 60 °E and 170 °W and has been documented in China, Russia, Japan, India, Eastern Australia, New Zealand, and some parts of the Pacific Islands. China, India, and Australia have experienced periodic outbreaks of oriental armyworm infestation^1^. Larvae of *M*. *separate* feed mainly on leaves, and the older larvae of 4–6 instars cause the main harm, typically leaving only the midrib uneaten. This results in heavy losses. *M*. *separate* overwinters during the pupa stage in the soil but also sometimes during the larval or adult stage. The moth of *M*. *separate* can migrate up to 1500 km. The moth undertakes a seasonal, long-distance, multi-generation roundtrip migration between southern and northern China each year^2^. To date, chemical control is still the major strategy to protect crops from damage by the oriental armyworm, leading to the development of resistance to many chemical insecticides. This species has been known to be a model migratory pest moth and is used as a test insect to explore new agricultural pesticides^3^.

At the start of this work, the genomic data available in public databases for *M*. *separate* are particularly scarce, and only 190 sequences, including 65 mRNA sequences, were accessible in the NCBI database for this economical pest. Even for the *Mythimna* genus that contains more than 288 species worldwide, only 102 mRNA sequences out of 1240 nucleotide sequences were available in NCBI. To address this issue and obtain a comprehensive understanding of *M*. *separate* molecular biology, more recent work based on *de novo* transcriptomic sequencing has led to the identification of insecticide resistance-related genes^4^. However, little is known regarding the underlying molecular mechanisms regulating the oriental armyworm physiological and behavioural responses.

Here, we provide a *de novo* transcriptome analysis of adult heads from *M*. *separate* using the Illumina HiSeq platform. An objective of this study is to characterize the transcriptome in the *M*. *separate* adult head. We chose the head because it is the sensory and feeding centre and supports the antennae. The brain inside of the head is the most important component of the central nervous system, playing a vital role in insect behaviour. We specifically focused on genes involved in migration, including the circadian clock and melanin synthesis pathway genes, as well as non-receptor genes related to olfaction. We provide a comprehensive list of genes related to these key processes in *M*. *separate*. In the monarch butterfly, *Danaus plexippus*, the circadian clock in the brain plays an important role in migration by providing the timing component of the time-compensated sun compass orientation^5, 6^. Therefore, it is possible that the circadian clock in the brain is involved in the induction of the oriental armyworm migration, as in *D*. *plexippus* ^7^. As an important neurotransmitter, dopamine modulates various aspects of insect behaviour, such as locomotor activity, decision making, phase change, learning and memory^8–10^, and it is also a precursor of melanin, which is a predominant insect pigment. Olfaction plays a key role in various insect behaviours, such as those related to locating suitable hosts, avoiding predators, identifying oviposition sites, and finding sexual partners^11^. In the present study, a total of 63,499 transcripts were generated by the adult head transcriptome data. Among these transcripts, we identified 14 homologs of the genes involved in the *Drosophila* clock, 20 melanin synthesis genes, and 53 olfaction-related genes by comprehensive phylogenetic analysis. Furthermore, the adult heads from multiple individuals also allowed us to examine the simple sequence repeat (SSR) feature in *M*. *separate*. These genes identified here can be valid targets for further gene function research to address the molecular mechanisms regulating oriental armyworm migration and olfaction.

## Results

### Sequencing and *de novo* assembly

To date, there remains no published genome for *M*. *separate*. Thus, for this study, we *de novo*-assembled and blast-annotated the head transcriptome for this species. Twenty-eight adult heads, excluding the eyes, were collected from a pool of animals in the morning, afternoon and evening to create the cDNA library. Transcriptomic sequence data were generated using the head cDNA library and Illumina HiSeq^TM^2500/MiSeq technology. We acquired 27,208,038 raw reads from the head transcriptome. After removing adapters, ambiguous nucleotides and low quality sequences, 3.27 Gbp of clean sequence data in 26,128,167 clean reads remained, with a Q20 value of 95.94% and a GC content of 46.02%. These clean reads were then randomly clipped into 25-mers for sequence assembly using Trinity software. The assembly resulted in 63,499 transcripts longer than 200 bp, which were further assembled into 46,459 unigenes, with an N50 of 1,153 and mean length of 690 bp, including 8,739 unigenes larger than 1,000 bp (Table S1). Paired-end reads that do not contain ambiguous bases were deposited in the NCBI Sequence Read Archive under the accession no. SRX2430648, and the assembled sequences were deposited in the NCBI Transcriptome Shotgun Assembly (TSA) under accession no. GFCT00000000, associated with Bioproject PRJNA357654.

To validate the reliability of the transcriptome sequence assembly, the nucleotide sequences of the 20 annotated unigenes from Illumina sequencing were selected to compare with those of the cDNAs obtained from Sanger sequencing (Table S2). These 20 cDNAs that were previously identified in *M*. *separate* by RT-PCR cloning were obtained from the NCBI nucleotide database. As we expected, the pairwise comparison exhibited a comparable alignment with 95–100% nucleotide identities. The results not only testified to the reliability of the assembly of the transcriptome and transcript annotation but also indicated that it could be useful for further research.

### Functional annotation of unigenes

For functional annotation of the *M*. *separate* head transcriptome, we searched all 46,459 unigene sequences against the non-redundant NCBI protein database using the Blastx tool with a cut-off E-value of 10^−5^. Using this approach, 19,973 unigenes (42.99% of all distinct sequences) returned a Blast hit in the Nr database, 12,703 unigenes (27.34%) had specific matches in the Swiss-Prot database, and 13,461 unigenes had matches in the Pfam database. In total, 22,334 (48.07%) unigenes were annotated in at least one database. Matches to the Nr database indicated that the silkworm *Bombyx* *mori* had the highest share of matches with 46.3%, followed by the butterfly *D*. *plexippus* with 30.0% (Figure S1).

**Figure 1:**
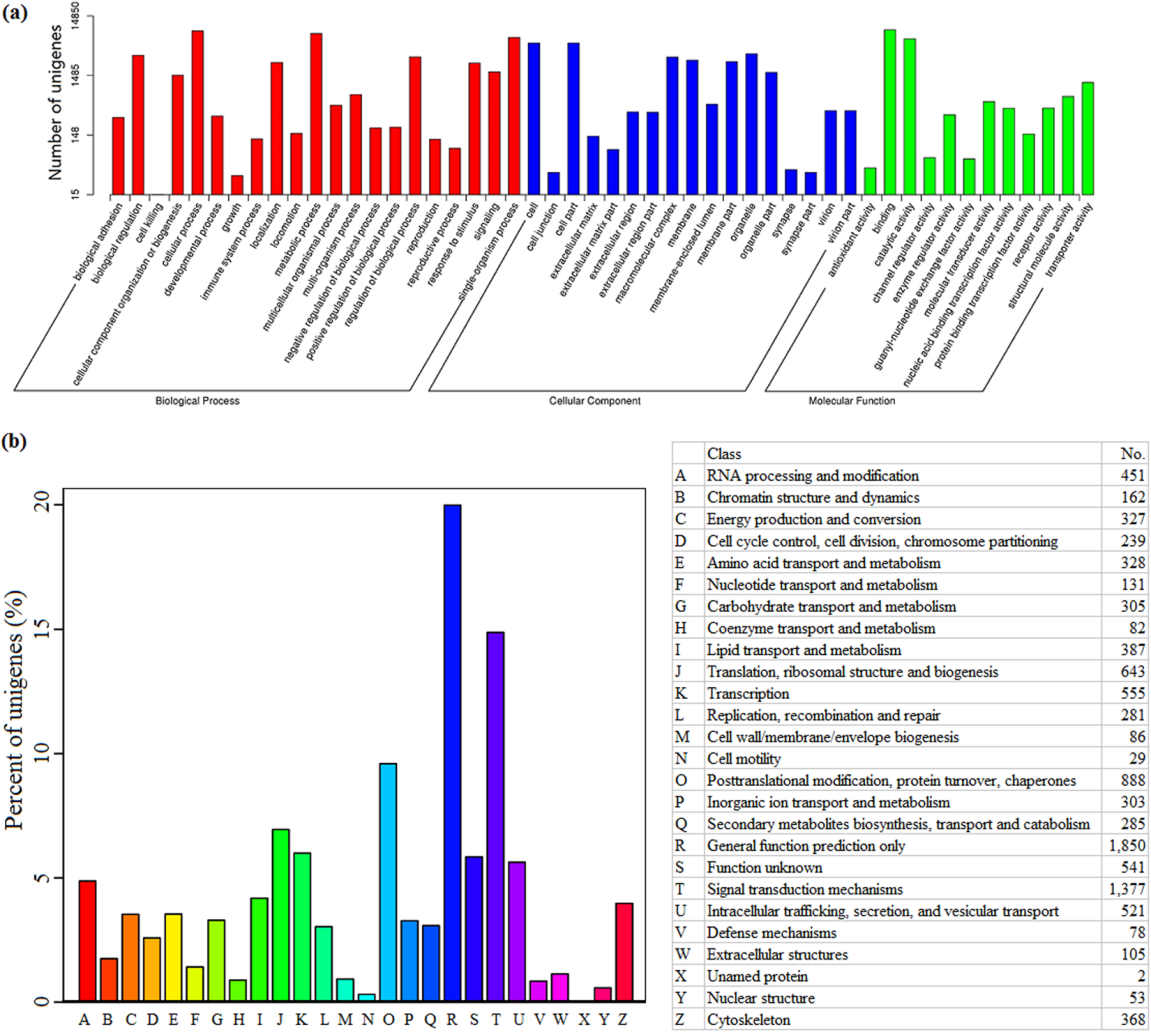
GO classification (**a**) and KOG categories (**b**) for the *M*. *separate* head transcriptome. The number of unigenes is shown on the right column for KOG categories.

Gene Ontology (GO) assignments were further employed to classify the functions of the *M*. *separate* unigenes. A total of 14,850 unigenes could be categorized into 59 functional groups (Fig. 1a). Biological processes, molecular functions, and cellular components were associated with 37,161 unigenes, 18,583 unigenes, and 26,024 unigenes, respectively. Within the biological process category, ‘cellular process’ (8,259 unigenes; 22.22%), ‘metabolic process’ (7,506 unigenes; 20.20%), and ‘single-organism process’ (6,434 unigenes; 17.31%) were the most abundant groups, whereas ‘biological phase’ (12 unigenes), ‘rhythmic process’ (8 unigenes), and ‘hormone secretion’ (5 unigenes) were the least abundant groups. In the cellular component category, the ‘cell’ (5181 unigenes; 19.91%) and ‘cell part’ (5179 unigenes; 19.90%) categories were highly represented, followed by the ‘organelle’ (3424 unigenes; 13.16%), ‘macromolecular complex’ (3030 unigenes; 11.64%), and ‘membrane’ (2687 unigenes; 10.32%) categories, whereas ‘nucleoid’ (2 unigenes), ‘collagen trimer’ (1 unigene), and ‘symplast’ (1 unigene) were the least abundant categories. In terms of molecular functions, ‘binding’ (8,637 unigenes; 46.48%) and ‘catalytic activity’ (6,119 unigenes; 32.93%) were enriched, whereas ‘receptor regulator activity’ (5 unigenes), ‘metallochaperone activity’ (4 unigenes), and ‘translation regulator activity’ (2 unigenes) were the least abundant categories.

The unigenes of *M*. *separate* were also characterized by KOG (euKaryotic Ortholog Groups) to enable conceptualization of its transcripts into potential functional groups. In total, 9,256 unigenes were annotated to 26 KOG categories (Fig. 1b). The KOG classification indicated that except ‘general function prediction’, genes involved in ‘signal transduction mechanisms’ (14.88%), ‘post translational modification, protein turnover, chaperones’ (9.59%), and ‘translation, ribosome structure and biogenesis’ (6.95%) were the most abundant.

**Figure 2:**
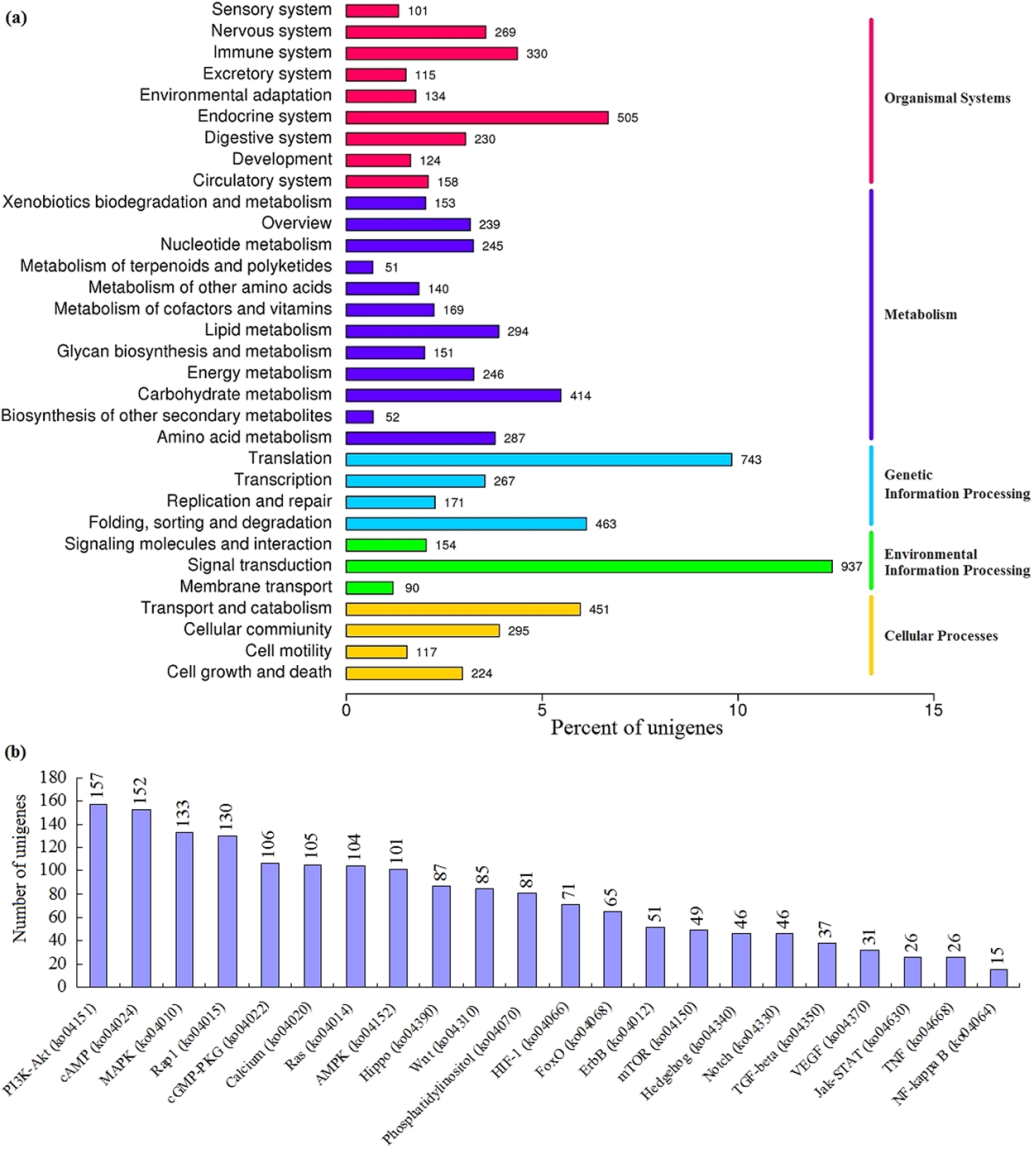
KEGG Orthology (KO) classification (**a**) and third-tier KEGG pathways in the signal transduction category (**b**) for *M*. *separate* head transcriptome. The number of unigenes is shown in the column. P13K-Akt exhibits the highest number of assigned unigenes in the signal transduction category.

Next, the unigenes were mapped to reference canonical pathways in the KEGG database, and 7,552 unigenes were assigned to KEGG Orthology (KO) terms and grouped into 264 pathways. These annotated pathways were clustered into five major categories (Fig. 2a). Among these pathways, ‘signal transduction pathways’ constituted the largest category, which contained 937 unigenes (12.41%), followed by ‘translation’ (743, 9.84%) and the ‘endocrine system’ (505, 6.69%) category. The 937 unigenes in signal transduction were assigned to 22 signalling pathways (Fig. 2b). Of these, the P13K-AKt, cAMP, and MAPK signalling pathways were ranked first to third according to the number of KEGG assignments. In addition, the nervous system (269), environmental adaptation (134), and sensory system (101) categories were well represented.

### Circadian clock genes

**Figure 3:**
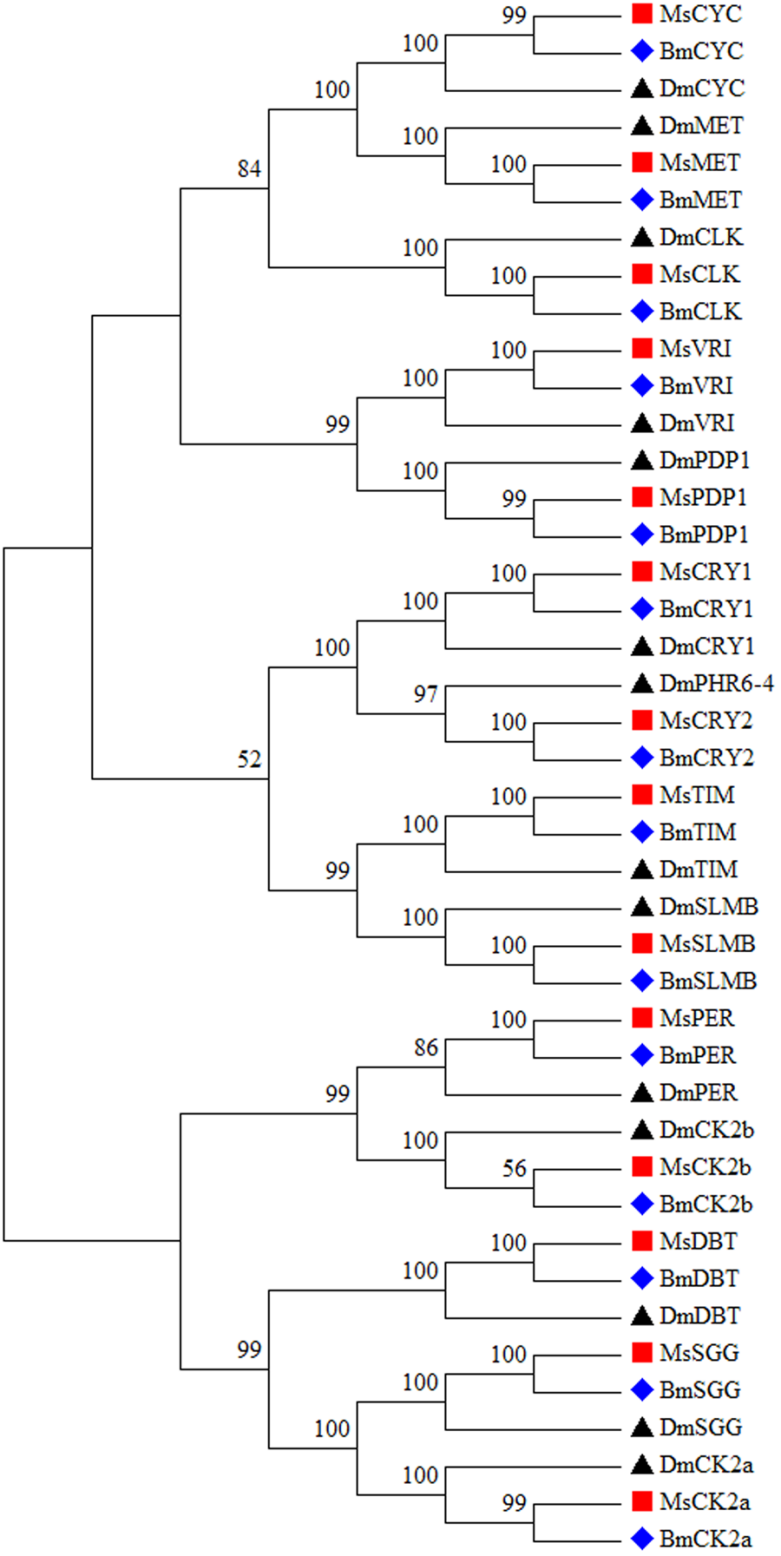
Neighbour-joining tree based on the amino acid sequences of the clock genes. CYC, cycle; MET, methoprene-tolerant; CLK, clock; VRI, vrille; PDP1, PAR-domain protein 1; CRY, cryptochrome; TIM, timeless; SLMB, slimb; PER, period; CK2b, casein kinase 2 beta; DBT, double time; SGG, shaggy; CK2a, casein kinase 2 alpha.

In the transcriptome data, fourteen homologs of the genes involved in the *Drosophila* clock were well represented, including *cryptochrome 1*, *cryptochrome 2*, *cycle*, *clock*, *vrille*, *timeless*, *slimb*, *period*, *double time*, *shaggy*, *PAR-domain protein 1*, *casein kinase 2 alpha*, *casein kinase 2 beta*, and *methoprene-tolerant* (Table S3). Ten of the 14 clock genes were full-length. The Blastx results indicated that the proteins encoded by these genes shared relatively high amino acid identities (65–98%) with lepidopteran species. We further confirmed these genes by comparing them with other known insect clock genes using phylogenetic analysis (Fig. 3). The RPKM values of these genes were also evaluated and, generally, *shaggy* was the most abundant.

### Melanin synthesis genes

**Figure 4:**
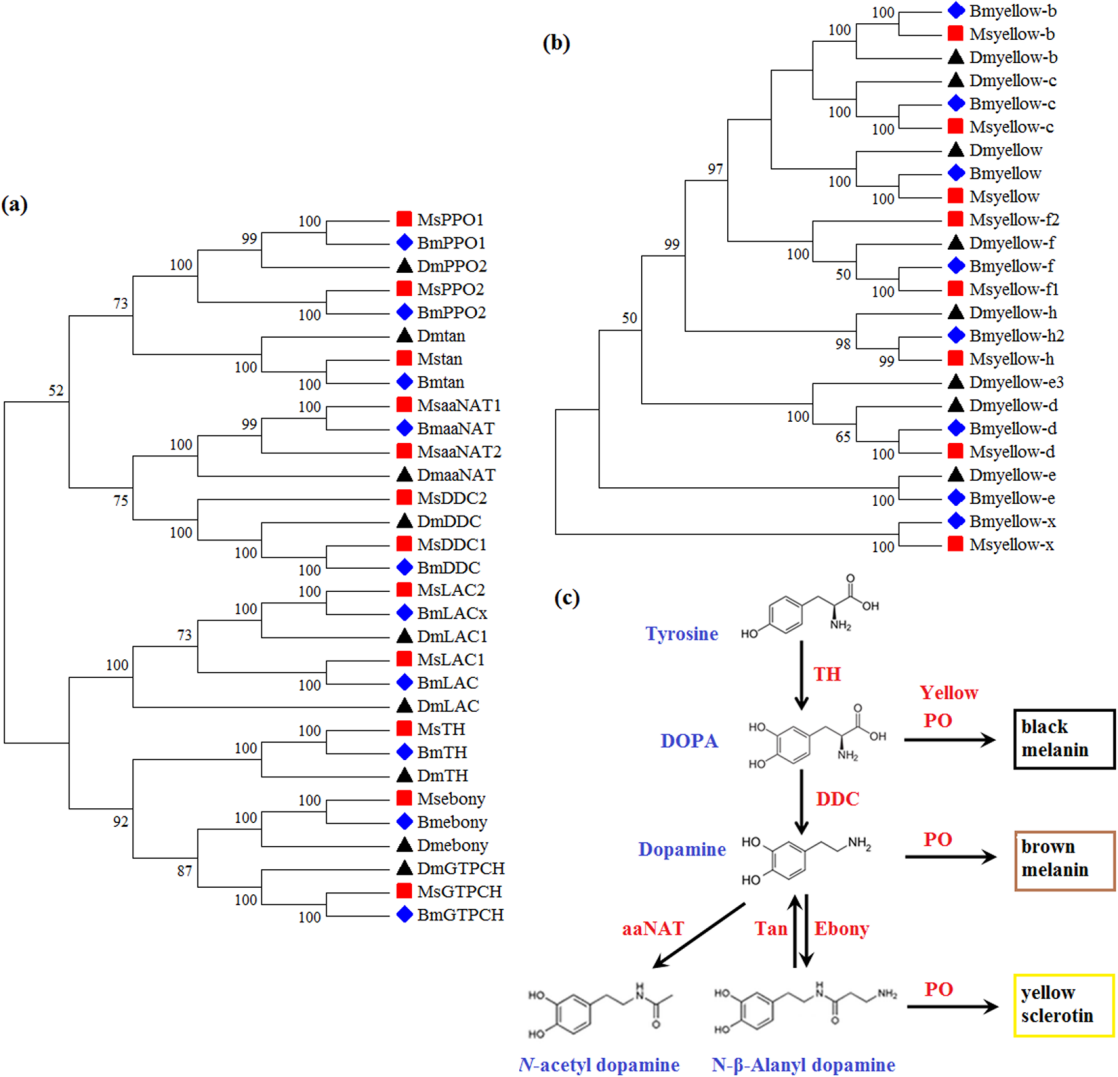
Neighbour-joining tree based on the amino acid sequences of melanin synthesis/related genes (**a**,**b**) and a schematic representation of the melanin synthesis pathway (**c**) found in *M*. *separate*. PPO, prophenoloxidase; tan, NBAD hydrolase; aaNAT, arylalkylamine N-acetyl transferase; DDC, DOPA decarboxylase; LAC, laccase; TH, tyrosine hydroxylase; ebony, NBAD synthase; GTPCH, GTP cyclohydrolase.

In this study, several transcript-encoding enzymes involved in the melanin synthesis pathway were identified in the *M*. *separate* transcriptome, including tyrosine hydroxylase, DOPA decarboxylase, arylalkylamine N-acetyl transferase, NBAD hydrolase, NBAD synthase, GTP cyclohydrolase, prophenoloxidase and laccase (Table S3). Two isoforms are found for genes encoding DOPA decarboxylase, arylalkylamine N-acetyl transferase, and prophenoloxidase. All the genes, except *prophenoloxidase 1*, are full-length. We also obtained all the yellow family genes in the *M*. *separate* transcriptome, excluding *yellow-e*, and the protein of each identified gene contained a conserved major royal jelly protein domain (Table S3). Six of the 8 yellow genes are full-length. The *yellow-x* gene showed the highest expression level in the yellow family genes. All these melanin synthesis genes were confirmed by phylogenetic analysis (Fig. 4a and b). The Blastx results indicated that the proteins encoded by these genes shared relatively high amino acid identities (51–98%) with lepidopteran species. These genes identified in *M*. *separate* represented almost all known enzymes involved in melanin synthesis in *Drosophila*. A brief schematic based on the melanin synthesis pathway in *Drosophila* ^12^ is shown in Fig. 4c.

### Olfactory genes

Due to the high sequence diversity of olfactory genes, their identification has largely only been possible with insects for which genomic data are available. It is very difficult to identify these genes by traditional homology-based methods. Recently, advances in RNA-Seq technology have opened up the possibility for such identifications in non-model organisms^13–15^. With respect to *M*. *separate*, only one pheromone-binding protein and three olfactory receptor genes have been identified. In this study, three non-receptor protein families involved in the detection of volatile substances were identified, including odorant binding proteins (OBPs), chemosensory proteins (CSPs), and sensory neuron membrane proteins (SNMPs) in the *M*. *separate* transcriptome (Table S3). We confirmed these olfactory genes by phylogenetic analyses based on the alignment of protein sequences from four lepidopteran species, including *M*. *separate*, *Spodoptera* *exigua*, *Dendrolimus* *kikuchii*, and *B*. *mori*.

**Figure 5:**
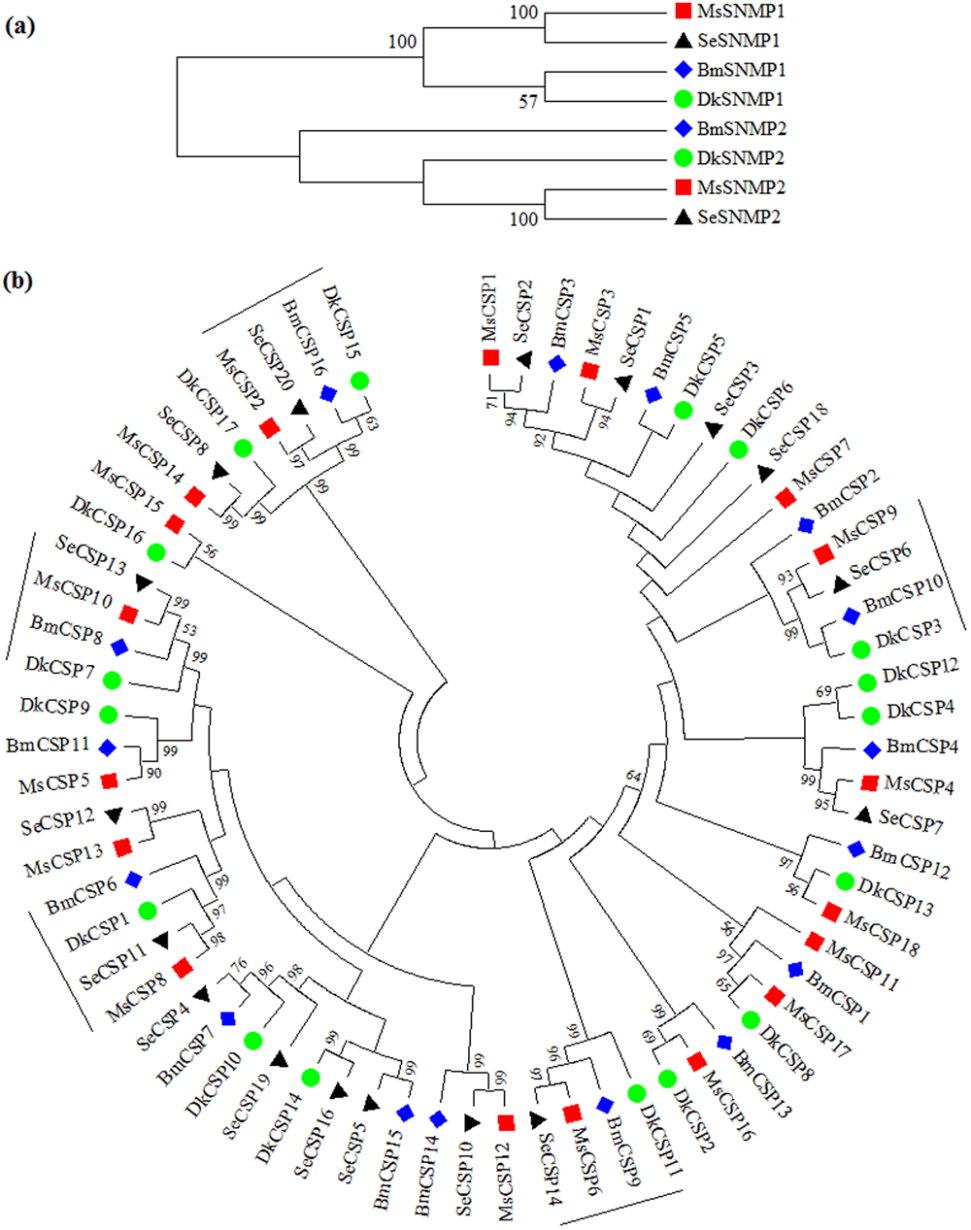
Neighbour-joining tree of candidate SNMP proteins (**a**) and CSP proteins (**b**). Five CSP orthologues (1:1:1:1) among four species are marked.

We identified two SNMPs (SNMP1 and SNMP2) in *M*. *separate*. Both were full-length genes. The SNMPs of *M*. *separate* were grouped with orthologues from other insect species (Fig. 5a) and shared more than 88% sequence identity with lepidopteran species.

A second group of non-receptor proteins is CSPs. We identified 18 transcripts encoding candidate CSPs in the *M*. *separate* transcriptome, a number comparable to 16 CSPs in *B*. *mori* ^16^, 17 in *D*. *kikuchii* ^14^ and *S*. *exigua*. Fifteen of 18 CSPs likely represent full-length proteins. The Blastx results indicated the 17 MsCSPs shared relatively high amino acid identities (59–99%) with lepidopteran CSPs. All the full-length amino acid sequences possessed a signal peptide and highly conserved four-cysteine profile (Figure S2). Although no obvious branches could be defined in the phylogenetic tree, five orthologues (1:1:1:1) could be found among four species (Fig. 5b).

**Figure 6:**
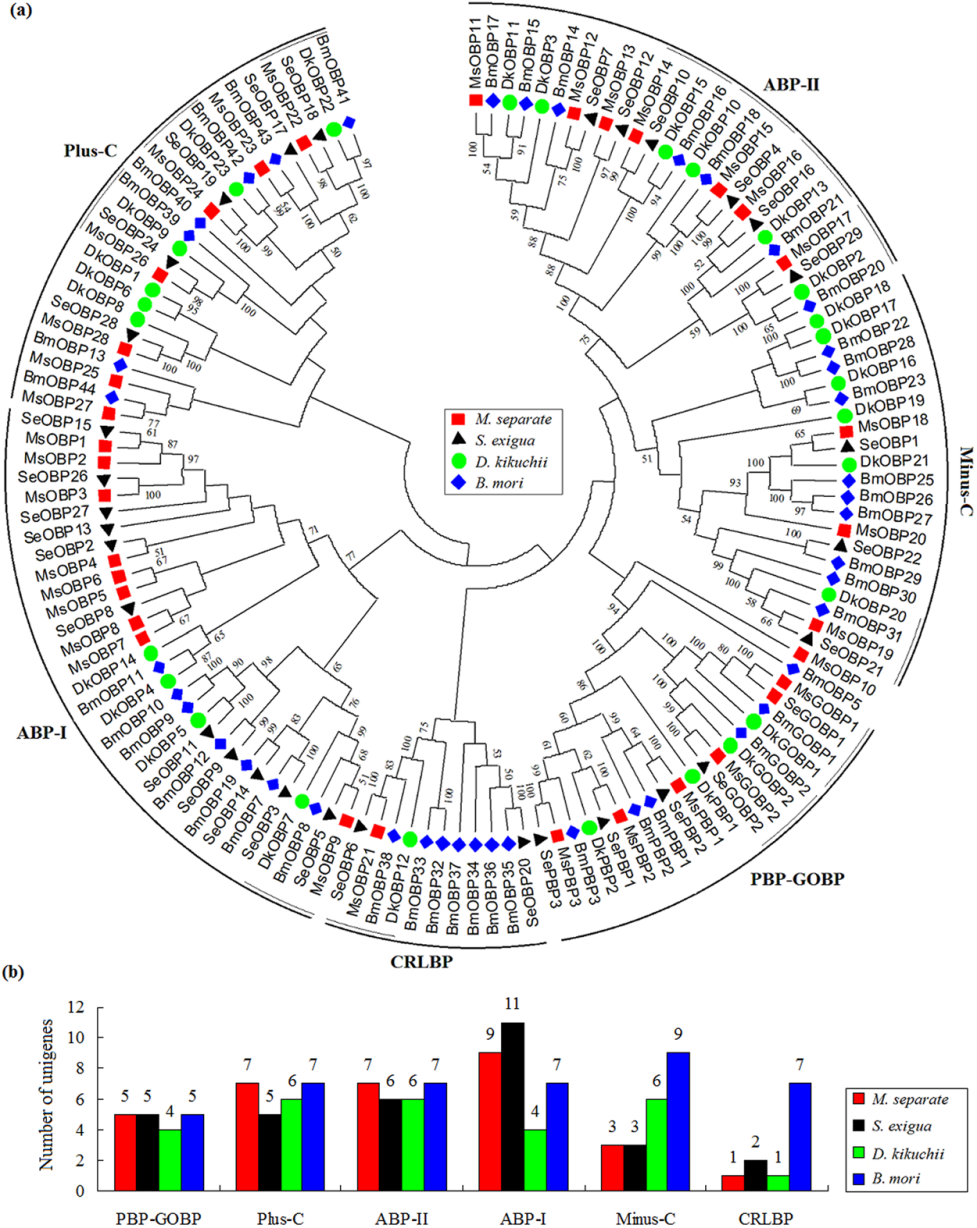
Neighbour-joining tree (**a**) and gene number comparison (**b**) of candidate OBP proteins among four lepidopteran species. Six groups, including PBP-GOBP, CRLBP, ABP-I, ABP-II, Plus-C and Minus-C, are marked on the dendrogram. Twelve orthologues (1:1:1:1) among four lepidopteran species are also marked.

A third class of non-receptor proteins identified in *M*. *separate* is the OBPs. A total of 38 candidate OBP genes were identified through Blastx analyses. A conserved domain search (CD Search) was further performed to confirm the superfamily of the candidate OBPs. Through a CD search, 5 candidates that contained the conserved JHBP domain (cl12117) were excluded, although each of them had a high sequence similarity at the protein level with the odorant binding proteins. Therefore, we identified 33 OBP genes in the *M*. *separate* transcriptome data, a number comparable to the 34 OBPs in *S*. *exigua*. Thirty of the 33 OBPs genes have intact ORFs, and the remaining three (*MsPBP2*, *MsPBP3*, *MsOBP9*) lack the 3’ end. The signal sequences are present at the hydrophobic N-terminus for all 33 OBPs. The Blastx results indicated that the 33 MsOBPs shared relatively high amino acid identities (33–98%) with lepidopteran OBPs. Both MsOBP25 and MsOBP28 have longer protein sequences than others, but each of them exhibits 79% and 69% identities with the OBP9 of *Spodoptera* *litura* (ALD65883) and OBP10 of *Ostrinia furnacalis* (BAV56797) and are associated with a query coverage of 100% and 91%, respectively. In the present study, six subfamilies of OBPs were defined based on their sequence comparison (Figure S2) and phylogenetic analysis (Fig. 6a). Following the description in *B*. *mori* ^16^ and *D*. *melanogaster* ^17^, we also named six subfamilies PBP-GOBP, CRLBP, ABP-I, ABP-II, Plus-C and Minus-C. The spacing pattern of the conserved six-cysteines in the *M*. *separate* OBP family is similar to those in *B*. *mori* and *D*. *melanogaster*. MsOBP18, MsOBP19 and MsOBP20 belong to the Minus-C subfamily, which is missing the conserved cysteines C2 and C5. Seven MsOBPs are defined as the Plus-C subfamily, but four of these do not have the PBP-GOBP motif (cl11600) when blasted in the CD Search (MsOBP22, MsOBP24, MsOBP26, MsOBP27). The conserved C2 and C3 of the four Plus-C OBPs (MsOBP22, MsOBP23, MsOBP24, MsOBP27) are separated by 4 amino acid residues rather than the usual 3 of the other OBPs, as observed in *S.* *litura* OBPs^18^. In the phylogenetic tree, many terminal relationships and four subfamilies (PBP-GOBP, Minus-C, ABP-I and ABP-II) were supported by high bootstrap values. Although the four lepidopteran species covered in this study are closely related, only twelve orthologues (1:1:1:1) could be found among the four species.

### SSR discovery

The identification of microsatellite polymorphisms is helpful for population genetics of this pest. Presently, no SSR markers are available for this species, although a preliminary information analysis of the SSR loci in *M*. *separate* has been reported^19^. In that study, only 400 potential SSR loci were identified in 372 unigenes. As we used 28 individual moths to construct the cDNA library, certain polymorphism levels are expected to be present in the transcriptome data. Here, the 46,459 unigenes assembled in this study were analysed to mine potential SSRs using the MISA package. A total of 5,267 potential SSRs were identified in 4,499 unigene sequences, of which 604 sequences contained more than 1 SSR. Among these SSRs, 228 were represented in compound formation, and 3895 were mononucleotide SSRs. As it is difficult to distinguish true mononucleotide repeats from polyadenylation sites, we did not include mononucleotide repeats in the following analysis. Therefore, 1,372 SSRs of 2–6 bp in unit length were identified (Table 1), which suggested a frequency of about one SSR per 23.35 kb of expressed sequences. Among all SSRs identified, trinucleotide repeats (63.78%) represented the most abundant microsatellite repeat units, followed by dinucleotide repeats (32.87%). Ten types of trinucleotide repeats were observed. Of these, CCG/CGG was the most abundant, followed by ATC/ATG and AAT/TTA. The CG/GC sequence was the most common among the dinucleotide repeat motifs, followed by AC/GT, AT/TA, and AG/CT. The AAAT/TTTA sequence was the most abundant in the tetranucleotide repeats. Most SSRs (48.91%) exhibited a repeat number of 5, and 37.24% of the SSRs had a repeat number of 6. The SSRs predicted in this study could lay a platform for better understanding the molecular ecology of this species. The primer information for the potential SSRs should be sent as a query.

**Table 1:** Repeat numbers and unit length distribution of SSRs in the transcriptome of *M*. *separata* unigenes.

SSR type/Repeats	Repeat numbers							Total	Percent %
	5	6	7	8	9	10	11~24		
Dinucleotide								451	32.9
AC/GT		88	25	17	9	4	5	148	
AG/CT		32	10	5	4	1		52	
AT/TA		60	14	7	10	2		93	
CG/CG		143	15					158	
Trinucleotide								875	63.8
AAC/GTT	21	5	1					27	
AAG/CTT	80	23		2				105	
AAT/TTA	136	17	7	2				162	
ACC/GGT	18	5	2					25	
ACG/CGT	25	9	2	1				37	
ACT/AGT	6	2						8	
AGC/CTG	32	8	3	1			1	45	
AGG/CCT	7	1	1					9	
ATC/ATG	116	43	14	2	1			176	
CCG/CGG	192	69	17	3				281	
Tetranucleotide								43	3.13
AAAC/GTTT	2							2	
AAAG/CTTT	7	1						8	
AAAT/TTTA	17	3						20	
AACC/GGTT	1							1	
AATC/ATTG	3							3	
ACAG/CTGT	2							2	
ACAT/ATGT	1				1			2	
ACCT/AGGT	1			1				2	
ACTC/AGTG	1							1	
ACTG/AGTC		1						1	
AGCG/CGCT	1							1	
Pentanucleotide								2	0.15
AAATC/ATTTG	1							1	
AATCG/ATTCG	1							1	
Hexanucleotide								1	0.07
AGCCTG/AGGCTC		1						1	
Total	671	511	111	41	25	7	6	1372	
Percent %	48.91	37.24	8.09	2.99	1.82	0.51	0.44		100

## Discussion

In the present study, we characterized the head transcriptome data of *M*. *separata* using the Illumina sequencing platform. We sequenced mRNA fragments from the heads of adult moths and assembled the transcriptome into 46,459 unigenes with a mean length of 690 bp. By searching against known nucleotide and protein databases, 22,334 (48.07%) unigenes were successfully annotated. The annotation rate obtained in this study was lower than that of a recent transcriptome study of *M*. *separate* (65.32%) in that a mixture of egg, larva, pupa and adult were used as samples, and the mean length of the unigenes was 551 bp^4^. This, however, is comparable to those of other Noctuidae insects, such as *Helicoverpa armigera* (50.8%)^20^, *H*. *assulta* (54.0%)^20^, *Spodoptera frugiperda* (51.1%)^21^ and *Athetis lepigone* (41.5%)^22^. The number of annotated unigenes in the *M*. *separate* head transcriptome is obviously lower than the pooled whole body transcriptome^4^, indicating that there is a need for using a standard method that incorporates all available tools and data information for annotating *de novo*-assembled transcriptomes in species without genome sequences^23^. The remaining unigenes that failed to generate homologous hits may be non-coding RNAs, new genes, or species-specific sequences. This is a common case for transcriptome analysis of non-model species where no published genome is available. Even in a prothoracic gland transcriptome of *B*. *mori* in which a full genome is available, we also found that 29.31% of the unigenes could not be annotated (unpublished data). In fact, many assembled sequences did not match significantly to the DNA/protein database due to their generally short sequence length or because they represented significantly different genes^24^.

In recent transcriptome work on *M*. *separata*, the butterfly, *D*. *plexippus*, had the highest share of matches^4^. However, our work revealed that the silkworm *B*. *mori* is the species that shows the most Blast hits for *M*. *separata*. According to the morphological characteristics, both *M*. *separata* and *B*. *mori* belong to the moth group, whereas *D*. *plexippus* belongs to butterfly group. Phylogenomics based on the mitochondrial genomes also confirmed that *M*. *separate* is closely related to *B*. *mori* rather than the butterfly^25^.

To our knowledge, this is the first attempt to investigate the head transcriptome of *M*. *separate*. The distribution of the most general GO terms in biological processes, molecular functions, and cellular components in the *M*. *separate* head transcriptome were very similar to those in the head transcriptomes of the South American fruit fly *Anastrepha fraterculus* ^26^ and the subterranean termite *Odontotermes formosanus* ^27^. Functional classification according to the KOG categories also exhibited an overall similarity between *M*. *separate* and *A*. *fraterculus*. The main similarity represented in the head transcriptomes of the two species was the ‘signal transduction mechanism’, while in the whole-body transcriptome of *M*. *separate* ‘post translational modification, protein turnover, chaperones’ represented the largest group^4^. A larger number of transcripts in the head of *M*. *separate* were annotated into the ‘signal transduction mechanisms’ group, demonstrating that signal transduction mechanisms were the most important in the adult head of *M*. *separate*.

To date, no studies have focused on the circadian clock of *M*. *separate*, although two clock genes, *cryptochrome 1* and *2*, have previously been isolated. We hypothesized that the circadian clock might be involved in the induction of oriental armyworm migration, as was found in *D*. *pleippus* ^7^. A nearly complete set of clock genes identified in this study indicated the presence of the clock in the head of *M*. *separate*. The identified genes provide a template for exploring how the circadian clock affects the future migration of the oriental armyworm.

Insect melanism is one of the common polymorphisms in nature. This was also reported in *M*. *separate* ^28^. Adults of the melanic form are almost completely darkened in comparison with those of the typical form, and the inheritance of melanic characteristics follows a Mendelian law. Although the melanic population possesses many developmental and reproductive advantages, it exhibits a reduced migration activity compared with the typical population^29^. To date, knowledge on the molecular mechanism controlling melanism in the oriental armyworm is very limited. Previous studies have indicated that insect melanism is tightly associated with the melanin synthesis pathway^12, 30^. The melanin synthesis genes identified here will contribute to further functional research on the molecular mechanism controlling melanism and influencing migration in the oriental armyworm.

Olfaction is critical for insects because of its use in most key insect behaviours. SNMPs, CSPs and OBPs are three members of the non-receptor olfactory gene families. Among the three olfactory gene families, SNMPs and CSPs are more conserved than OBPs^14, 31^, which was also confirmed by our results. We identified comparable numbers of CSPs with those found in *B*. *mori*, *D*. *kikuchii* and *S*. *exigua*. Generally, SNMPs have two orthologues in insects^32^, and we also found both in *M*. *separate*. The exact functions of OBPs are still unknown, although their most important function is suggested to be involved in the capture and delivery of outside odorants to the odorant receptors. In this study, we identified 33 OBP genes from the *M*. *separate* head transcriptome, of which 32 *MsOBPs*, except *MsPBP1*, are reported here for the first time. The number of *M*. *separate* OBPs identified in this study was comparable with the numbers from the antennal transcriptomes of *Agrotis ipsilon* (33)^33^, *Spodoptera littoralis* (36)^34^, and *S*. *litura* (38)^18^. The topology of OBPs in our phylogenetic tree is largely consistent with the phylogenetic relationship established from six lepidopteran species, including *B*. *mori*, *A*. *ipsilon*, *Helicoverpa armigera* and three *Spodoptera* species^18^. For the OBP numbers, subfamilies of PBP-GOBP, Plus-C and ABP-II are comparable among the four species, whereas each of the Minus-C and CRLBP subfamilies show lineage-specific expansion and diversification in *B*. *mori* (Fig. 7b). The increase of OBP number in the two subfamilies in *B*. *mori* may be related to its long-term artificial selection or different adaptation responses. These genes could be valid targets for further gene function research. Further research on the molecular mechanism of olfaction in *M*. *separate* based on the data in this study will be helpful for the more efficient control of this pest.

## Materials and Methods

### Insects

A *M*. *separate* colony has been reared continuously in the laboratory of the pest group at the Institute of Plant Protection, Chinese Academy of Agricultural Sciences (Beijing, China). The larvae were reared on fresh maize leaves at 25 ± 2 °C and 70 ± 5% relative humidity under a 12:12 light:dark photoperiod. A total of 28 adult heads, excluding eyes, were collected for construction of the cDNA library. Heads were collected in the morning (10), afternoon (9) and evening (9). Then, head samples were immediately frozen in liquid nitrogen and subsequently stored at −80 °C until use.

### RNA isolation and sequencing library preparation

Frozen head samples were shipped to Novogene (Beijing, China) for RNA isolation and library construction. Total RNA isolation was extracted from the heads of *M*. *separata* using TRIzol reagent (Invitrogen, Carlsbad, CA, USA). RNA degradation and contamination was monitored on 1% agarose gels. RNA purity was checked with a NanoPhotometer spectrophotometer (IMPLEN, CA, USA). RNA concentration was measured using a Qubit RNA Assay Kit with a Qubit 2.0 Fluorometer (Life Technologies, CA, USA). The RNA Nano 6000 Assay Kit of the Agilent Bioanalyzer 2100 system (Agilent Technologies, CA, USA) was used to assess the RNA integrity. A total amount of 3 μg of RNA was used as input material for the RNA sample preparations. Sequencing libraries were generated with NEBNext Ultra™ RNA Library Prep Kit for Illumina (NEB, USA). To select cDNA fragments of preferentially 150~200 bp in length, the library fragments were purified with the AMPure XP system (Beckman Coulter, Beverly, USA). The size-selected, adaptor-ligated cDNA fragments were enriched by PCR with Phusion High-Fidelity DNA polymerase, Universal PCR primers and Index (X) Primer. Lastly, PCR products were purified (AMPure XP system), and the quality of the library was assessed on the Agilent Bioanalyzer 2100 system. The clustering of the index-coded samples was performed on a cBot Cluster Generation System using TruSeq PE Cluster Kit v3-cBot-HS (Illumina, CA, USA).

### *de novo* assembly and functional annotation

The cDNA library was sequenced on an Illumina HiSeq 2500 platform, and paired-end reads of 2 × 125 bp in size were generated. The *de novo* assembly pipeline for the *M*. *separate* head transcriptome was outlined in Figure S3. Raw fastq data (raw reads) were first processed through in-house Perl scripts. In this step, clean data (clean reads) were obtained by removing reads containing the adapter, ploy-N and low quality reads from the raw data. At the same time, Q20, Q30, GC-content and the sequence duplication level of the clean data were calculated. The left files (read1 files) from all the libraries/samples were pooled into one big left.fq file, and the right files (read 2 files) into one big right.fq file. Transcriptome assembly was accomplished based on the left.fq and right.fq files using Trinity^35^ with a Kmer_length of 25 and min_kmer_cov set to 2, and all other parameters set to default. Function annotation was performed based on the following databases: Nr (NCBI non-redundant protein sequences), Nt (NCBI non-redundant nucleotide sequences), Pfam (Protein family), KOG/COG (Clusters of Orthologous Groups of proteins), Swiss-Prot (A manually annotated and reviewed protein sequence database), KO (KEGG Ortholog database), and GO (Gene Ontology).

### Sequence analysis

The similarity searches were performed with the NCBI-Blast network server (http://blast.ncbi.nlm.nih.gov/). Putative N-terminal signal peptides of proteins were predicted by the Signal IP 4.1 server (http://www.cbs.dtu.dk/services/SignalP/)^36^. The detection of the conserved protein domains was carried out with a batch CD-search tool in NCBI^37^. The expression abundance of the unigene was calculated based on the reads per kilobase per million mapped reads (RPKM) method^38^.

### Phylogenetic analysis

The accession numbers of sequences used for phylogenetic analysis are listed in Table S4. For phylogenetic analysis of migration-related genes, we included *M*. *separate* (Ms), and two model insects, *B*. *mori* (Bm) and *Drosophila melanogaster* (Dm). For olfactory genes, we included four lepidopteran species, *M*. *separate*, *B*. *mori*, *D. kikuchii* (Dk) and *S. exigua* (Se). Amino acid sequences were aligned with ClustalX 1.83^39^, and unrooted trees were constructed with MEGA6.0^40^ using the neighbour-joining method, with Poisson correction of distances and bootstrap replications set at 1000.

### SSR detection

Picard - tools v1.41 and samtools v0.1.18 were used to sort, remove duplicated reads and merge the bam alignment results. SSRs were identified using MISA (http://pgrc.ipk-gatersleben.de/misa/misa.html), and primers for each SSR were designed using Primer3 (http://primer3.sourceforge.net/releases.php).

## Electronic supplementary material

Characterization of the Adult Head Transcriptome and Identification of Migration and Olfaction Genes in the Oriental Armyworm Mythimna separate
