# Supplementary material for: Characterization of the Adult Head Transcriptome and Identification of Migration and Olfaction Genes in the Oriental Armyworm Mythimna separate
Source: Sci Rep. 2017 May 24;7:2324. doi: 10.1038/s41598-017-02513-6 (PMC5443819; doi:10.1038/s41598-017-02513-6)
Supplement: Supplementary file 1 — Characterization of the Adult Head Transcriptome and Identification of Migration and Olfaction Genes in the Oriental Armyworm Mythimna separate [file 41598_2017_2513_MOESM1_ESM.doc]

**Supplementary information**

**Characterization of the Adult Head Transcriptome and Identification of Migration and Olfaction Genes in the Oriental Armyworm *Mythimna separate***

Hai-Xu Bian^1,†^, Hong-Fang Ma^1,†^, Xi-Xi Zheng^1,†^, Ming-Hui Peng^1,†^, Yu-Ping Li^1,†^, Jun-Fang Su^2^, Huan Wang^1^, Qun Li^1^, Run-Xi Xia^1^, Yan-Qun Liu^1,*^ & Xing-Fu Jiang^3,*^

^1^ Insect Resource Center for Engineering and Technology of Liaoning Province, College of Bioscience and Biotechnology, Shenyang Agricultural University, Shenyang 110866, China.

^2^School of Basic Medicine, Guangzhou University of Chinese Medicine, Guangzhou 510006, China.

^3^State Key laboratory for Biology of Plant Diseases and Insect Pest, Institute of Plant Protection, Chinese Academy of Agricultural Sciences, Beijing 100193, China.

*Author for correspondence

Yan-Qun Liu, PhD. College of Bioscience and Biotechnology, Shenyang Agricultural University, Shenyang 110866, China. Email: [liuyanqun@syau.edu.cn](mailto:liuyanqun@syau.edu.cn).

Xing-Fu Jiang, PhD. State Key laboratory for Biology of Plant Diseases and Insect Pest, Institute of Plant Protection, Chinese Academy of Agricultural Sciences, Beijing 100193, China. Email: [xfjiang@ippcaas.cn](mailto:xfjiang@ippcaas.cn).

**Supplementary Figures**

**Supplementary Figure 1.**

Species distributions from Blastx matches of *M. separate* transcriptome unigenes.

**Supplementary Figure 2.**

Alignment results of MsCSPs (a) and MsOBPs (b).

**Supplementary Figure 3.**

*de novo* assembly pipeline for the *M. separate* head transcriptome

**Supplementary Tables**

**Supplementary Table 1.**

Summary statistics and annotation of *M. separate* head transcriptome.

**Supplementary Table 2.**

Comparison of 20 known genes with those obtained in transcriptome data.

**Supplementary Table 3.**

Candidate migartion and olfactory genes identified in *M. separate*.

**Supplementary Table 4.**

The accession numbers of sequences used for phylogenetic analysis.

**Supplementary Figure 1.**

**
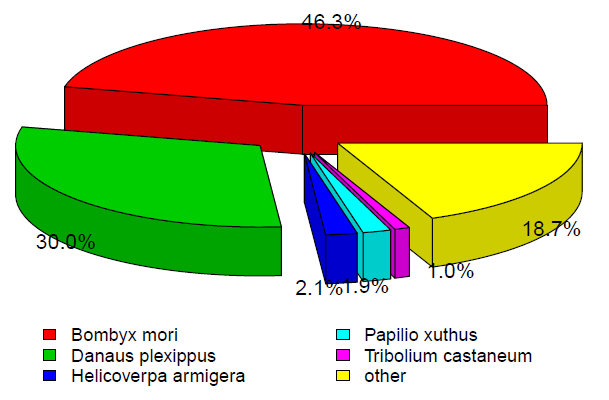
**

**Supplementary Figure 2.**

**(a) Alignment results of MsCSPs**

MsCSP1 -------MKSYIALLVLSVAAMALA---RPEEAKYTDRYDNVNLDEVLSNRRLLVPY 47

MsCSP3 -------MNSLTVLCLFALVALAVA---RPD-GKYTDRYDSVNLDQILSNRRLLVPY 46

MsCSP4 -------MK---FVLLLCVMVAAVV---ADD--KYTDKYDNIDLDEILSNKRLLDAH 42

MsCSP11 --------------LVAVAITLACV---QAAEDKYSSKYDNINLDEVLGNKRLLNGY 40

MsCSP17 -------MN---FLVLSVVVTLAAF---AAADLTYTDRYDHVNVDEILDNRKLLVPY 44

MsCSP9 -------MKLIVAVALLCVVAMAWG----KPASTYTDKWDNINVDEILESQRLLKAY 46

MsCSP18 ------MMMLYSSLAMMLLTYLTIQS-NATETSTYTTKYDGIDLDEILNNERLLTGY 50

MsCSP7 -------MK---TLFILCALVIAVS---ARPEEQYTTEYDNIDIDEILNNDRLFKSY 44

MsCSP16 -------MK---VVLLTLCLALGVLA-----QDKYESANDDFDVSEVLSNPRLLNSY 42

MsCSP5 -------MKADCVLLATLMAVVA--------ADFYSSKYDSFDVQPLLENDRILLSY 42

MsCSP10 -------MKTILVLCVLIAAVCARP------EATYDTRYDNFDVESLVENVRLLKSY 44

MsCSP8 -------MRVLIVLSCLVVLAFA--------AEKYNAKYDNFDVETLISNDRLLKAY 42

MsCSP13 -------MKLLIVLALVAAALARPD------DSHYDEKYDNFNIDEVITNERLLKNY 44

MsCSP6 MVNLFLKMRAVFVLCVLVYVVVGQELNDMGNMPKYDSRYDYLDVDAIFTNKRLVRNY 57

MsCSP12 -------MKTWLLCLCVLTVVVSCY-------SQGPNRYENFNADAIIQNDRILLAY 43

MsCSP2 -------MQIKYALVLCCVAAVSLA-------QTQRPAVSDTADEALVLQGVVEQRQ 43

MsCSP14 -------MQIIILTALCVGLVAGLH-------VQAGPQMTDAQLEQTLADKNTMQRH 43

MsCSP15 -------MKVLIVLTALVAFAAAAA-----LTPEELKMLEAFDFDALFANDEQRKIV 45

MsCSP1 VKCILDQGKCAPDGKELKEHIKEALENECGKCTETQKSGTRRVIGHLINHEDAYWRELTA 107

MsCSP3 IKCMLDQGKCTPDGKELKTHIREALEQDCAKCTKAQRDGTRQVMGHLINHEVDYWNELKA 106

MsCSP4 YKCVMDKGKCTAEGKELKDHLTEAIENGCAKCTENQEKGAQKVIDHLIKNELDMWRELAA 102

MsCSP11 MKCTLDQGPCTAEGKELKYYISDGLKTGCSKCTPRQRKGVKKVMKY-------------- 86

MsCSP17 IKCTLDQGRCTPDGKELKAHIKDAMQTGCAKCTKKQKKAAKKVVKHIRAKEQDYWKQIVN 104

MsCSP9 VDCLMDRGRCTPDGKALKETLPDALENECSKCTEKQKSGSDKVIRHLVNKRPDLWKELST 106

MsCSP18 VNCLMDNGPCTADGKELKKNIPDAIENDCKKCTDRQRDGSDRVMHYLIDHRPDDWVKLEE 110

MsCSP7 FECLVGEGKCTPAGKELKSHMPDALQTECSKCSPKQKEGTKKVMKFLINNKPEQWKRLCA 104

MsCSP16 SKCLLNQGPCTPEVKQVKEKLPEALETRCAKCTDKQKQMGKALAQEVKKNHPDIWKQLVA 102

MsCSP5 TKCFLDEGPCTPDAKDFKKVIPEALETTCGKCSPKQKQLIRMVVKAVIERHPEAWQQLSD 102

MsCSP10 GHCFLGTGPCTPEGSAFKKTIPDALQTGCGKCSPRQRHLIRVVVNGFQTKTPDIWKQLVK 104

MsCSP8 INCFLDKGRCTPEGSDFKKTLPEAIETTCAKCTEKQKGNIRKVIKAIQQKHPKEWDDLVK 102

MsCSP13 AHCLIGDGKCTPEGNEFKKLLPEATKSNCGKCTDKQKVHVAKAIKAIKEKLPTEYETLRS 104

MsCSP6 VDCLINSVRCSPEGKALKRILPEALRTKCVRCTERQKRAAVKVIRRLKNDFPEEWSKLAS 117

MsCSP12 YKCVMDKGPCTRDGKNFKRVLPETLATACGRCNPAQKTIVRKLLLGIRTKSE-------- 95

MsCSP2 LKCALGEAPCDPIGKRLKTLAPLVLRGACPQCTPQETKQIQRTLSYVQRNFPQQWAKIVR 103

MsCSP14 IKCALGEGPCDPVGRRLRTLAPLVLRGACPQCSMQETRQIRRTLAFVQRNYPWEWAKIVR 103

MsCSP15 FDCMLDKGDCGP-YKQLVELSTKTVTTKCADCSPAQKTKYDYVLKVLHDKYEPVYTEFLK 104

MsCSP1 KYDPQRKFTAKYEKELKEIKQ------------------ 128

MsCSP3 KYDPKNLYSTKHEQELRKLKQ------------------ 127

MsCSP4 KYDPTGNWRKKYEDRARAAGIVIPAE------------- 128

MsCSP11 ---------------------------------------

MsCSP17 KYDPGNEYTETYEAFLASPDESK---------------- 127

MsCSP9 KYDPDNIYQDKYKT--QIESAKQ---------------- 127

MsCSP18 KYNSDGSYKMKYLSSKKTEDSKETNGTKSEEETKNSSKE 149

MsCSP7 KYDPEGKYASKYEKELKEVSQ------------------ 125

MsCSP16 MYDPQGKYQQAWQDFLKE--------------------- 120

MsCSP5 KFDKDRKFKDSFDKFLAEED------------------- 122

MsCSP10 KEDPNGEFKETFTRFLKASD------------------- 124

MsCSP8 KNDPSGKNRANFDKFIQGSR------------------- 122

MsCSP13 QIDPEGAHAEDINKYVAKYAP------------------ 125

MsCSP6 RWDPT---------------------------------- 122

MsCSP12 ---------------------------------------

MsCSP2 QYAG----------------------------------- 107

MsCSP14 QYG------------------------------------ 106

MsCSP15 KANAKKE-------------------------------- 111

**(b) Alignment results of MsOBPs**

MsOBP1 1 ------------------MSKFTCLVFFIVAASISKAY--ASEEEKAAFREAVKPIIEEC
MsOBP2 1 ------------------MSKFTCLVLCVVAVSISRAY--ASEEDKAAFRAAIQPIVDEC
MsOBP3 1 ------------------MSKFTCIVFCIVAASLTKVSHAVTEEEKAAFREVMAPIIDEC
MsOBP4 1 ------------------MFKLAYLLFCAVAVSLSGIVR-AGEEDAEAFREAIKPFITEC
MsOBP7 1 ------------------MYKFTCFVFYILYAVFTQAE---SDSSDSGSDEVFDKLSHEC
MsOBP8 1 ------------------MFKSTSLILYAVAVSLSNAN---DD----GSKE-FVSMVDEC
MsOBP5 1 ------------------MSKFSCLAFCVVVVSLNSVLAE----DGPANEGDVLDIVFEC
MsOBP6 1 ------------------MSEFMS-VLCAIVMSLNSVYT-----DAPLGLGDINTILLDC
MsOBP9 1 -----------------MFNVYFCVFVCGVLSLNIKASS------LDDLKLKYVEVIIEC
MsOBP11 1 ------------MNTSNFQSIFCIICIVSLFFSYSHAMT------RQQLKNSGKLMKKSC
MsOBP13 1 ----------MLYSGSVFLLSFMLIMLNSSFIS---AMT------REQVKSSGKMIKKTC
MsOBP12 1 ------------MD----RKGLCLLIVAMFLATGSDAMS------RQQLKNSGKVLKKNC
MsOBP15 1 -------------------MLLIEIVKFLILVAMCEAMT------MKQIRNTGKMMRKSC
MsOBP14 1 ------------------MNQSYLLLLIAACVEISYGMT------RAQVKKTMTIIKNQC
MsOBP16 1 -----------------MFTGTLPVVLCLVAAAYGGKEK---PVFSDEIKEIIQTVHDEC
MsOBP17 1 -----------------MTMWFRALAMLVAGLAAAQAIE-----MDEDMAELARMVRESC
MsOBP18 1 ------------------MKSFVVFCLVLVVGVYANVTL------PPTQQEKAQKLAAEC
MsOBP19 1 ------------------MKTFLVLAACILLAQGLTDEQ----------KEKLKKHNTEC
MsOBP20 1 ------------------MKLFVVLCIVLVTEIYAAYVP------LPPDHTDSN--LEEC
MsGOBP1 1 --------------------MTPTTLVLALGLAAALADVN----VMKDVTLGFGQALDKC
MsGOBP2 1 --------------------MTSKCGLLLAVMAAVAGSVMGTAEVMSHVTAHFGKALEEC
MsPBP1 1 -------------MVLHRSATMSARLALVVIASLFI-AVECSQEIMKNLAINFAKPLEDC
MsPBP2 1 ------------------MADSRMRLACLVCVIFVASSAMASKELLTKMSSGFTKVVDQC
MsPBP3 1 ------------------MGRNCIFFALVLMAVGVK-EIAPSKDAMKYITSGFVKVLEEC
MsOBP10 1 -----------------MIKFSVVCLYFVVVAVHFWNVKCMTKDQEQEIIKAMKPLAEEC
MsOBP21 1 MTKVLLATVLIVITFALTRAASTQMKDAMPKEPMTTTTMANQDSSIDSTDIDVIAVMNAC
MsOBP25 1 -------------------MCLINYHVLILCLILVESYALNCRSSGGPKEAELKNIYKKC
MsOBP28 1 --------------------MVGVVVPTVLLALLPAWVASSGEGNIKLLENEVAIALKAC
MsOBP26 1 -----------MVRKIGGLLCCLCVFGISLSDSAISADSESRCRNPPTAPQKIERVITLC
MsOBP27 1 -------------------------MYRLVILSIVAVSALADEMGMRECGRMFHPHSVRC
MsOBP23 1 ------------------MFKWLIIVALVAASYGDPISE------SRDNKSATLKPLSVC
MsOBP22 1 ------------------MFKFCVFLAFCVAASYGAPGG-------GTYCGETPSVIYQC
MsOBP24 1 ------------MIRSCLVLAAVFQVLFGQESGPDPRDGFRQPVPHYCLSPPPGTDLHKC

MsOBP1 41 SKEHGVG-----------------------------------------------------
MsOBP2 41 SKEHGVS-----------------------------------------------------
MsOBP3 43 SGEHGVS-----------------------------------------------------
MsOBP4 42 AKEHGIS-----------------------------------------------------
MsOBP7 40 MEKFGVT-----------------------------------------------------
MsOBP8 35 ARLNGHT-----------------------------------------------------
MsOBP5 39 AKENEVK-----------------------------------------------------
MsOBP6 37 AKENKVT-----------------------------------------------------
MsOBP9 38 SNDYPIT-----------------------------------------------------
MsOBP11 43 MPKNDVT-----------------------------------------------------
MsOBP13 42 SVKNNLS-----------------------------------------------------
MsOBP12 39 MNKNQVT-----------------------------------------------------
MsOBP15 36 QPKNNVE-----------------------------------------------------
MsOBP14 37 MPKNSVT-----------------------------------------------------
MsOBP16 41 VGKTGVA-----------------------------------------------------
MsOBP17 39 AAETGAD-----------------------------------------------------
MsOBP18 37 VKESGVS-----------------------------------------------------
MsOBP19 33 LAETKVD-----------------------------------------------------
MsOBP20 35 RKTSEFT-----------------------------------------------------
MsGOBP1 37 RQESDLT-----------------------------------------------------
MsGOBP2 41 REESGLS-----------------------------------------------------
MsPBP1 47 RKEMDLP-----------------------------------------------------
MsPBP2 43 KNELNVG-----------------------------------------------------
MsPBP3 42 KHELNMN-----------------------------------------------------
MsOBP10 44 ASYCGLK-----------------------------------------------------
MsOBP21 61 NESFRIE-----------------------------------------------------
MsOBP25 42 LKMQEGKNSSRGNS----EQDYKEPRGQIQRSDWERGRTTGSKENKNGRDDRMSGKDRKG
MsOBP28 41 TYPEETTASKDGTSKERQRRSDDYDGSPRIDNNMKEGNRYSHERRNNDSGDQMMVLNATD
MsOBP26 50 QDEIKLSILR--------------------------------------------------
MsOBP27 36 CKKTSELKDKFMLS----------------------------------------------
MsOBP23 37 CDIPELG-----------------------------------------------------
MsOBP22 36 LNSPKVIS----------------------------------------------------
MsOBP24 49 CPIPKLFP----------------------------------------------------

MsOBP1 48 ------------------------------------------------------------
MsOBP2 48 ------------------------------------------------------------
MsOBP3 50 ------------------------------------------------------------
MsOBP4 49 ------------------------------------------------------------
MsOBP7 47 ------------------------------------------------------------
MsOBP8 42 ------------------------------------------------------------
MsOBP5 46 ------------------------------------------------------------
MsOBP6 44 ------------------------------------------------------------
MsOBP9 45 ------------------------------------------------------------
MsOBP11 50 ------------------------------------------------------------
MsOBP13 49 ------------------------------------------------------------
MsOBP12 46 ------------------------------------------------------------
MsOBP15 43 ------------------------------------------------------------
MsOBP14 44 ------------------------------------------------------------
MsOBP16 48 ------------------------------------------------------------
MsOBP17 46 ------------------------------------------------------------
MsOBP18 44 ------------------------------------------------------------
MsOBP19 40 ------------------------------------------------------------
MsOBP20 42 ------------------------------------------------------------
MsGOBP1 44 ------------------------------------------------------------
MsGOBP2 48 ------------------------------------------------------------
MsPBP1 54 ------------------------------------------------------------
MsPBP2 50 ------------------------------------------------------------
MsPBP3 49 ------------------------------------------------------------
MsOBP10 51 ------------------------------------------------------------
MsOBP21 68 ------------------------------------------------------------
MsOBP25 98 GSSMRDRDDMMGRTDDRMDRNDDRNNRNDDRMSSNNDRSGGRGRMGGNNNRNDMSRGRDD
MsOBP28 101 YDYEGYGTGNMG------------------------------------------------
MsOBP26 60 ------------------------------------------------------------
MsOBP27 50 ------------------------------------------------------------
MsOBP23 44 ------------------------------------------------------------
MsOBP22 44 ------------------------------------------------------------
MsOBP24 57 ------------------------------------------------------------

MsOBP1 48 ------------------------------------------------------------
MsOBP2 48 ------------------------------------------------------------
MsOBP3 50 ------------------------------------------------------------
MsOBP4 49 ------------------------------------------------------------
MsOBP7 47 ------------------------------------------------------------
MsOBP8 42 ------------------------------------------------------------
MsOBP5 46 ------------------------------------------------------------
MsOBP6 44 ------------------------------------------------------------
MsOBP9 45 ------------------------------------------------------------
MsOBP11 50 ------------------------------------------------------------
MsOBP13 49 ------------------------------------------------------------
MsOBP12 46 ------------------------------------------------------------
MsOBP15 43 ------------------------------------------------------------
MsOBP14 44 ------------------------------------------------------------
MsOBP16 48 ------------------------------------------------------------
MsOBP17 46 ------------------------------------------------------------
MsOBP18 44 ------------------------------------------------------------
MsOBP19 40 ------------------------------------------------------------
MsOBP20 42 ------------------------------------------------------------
MsGOBP1 44 ------------------------------------------------------------
MsGOBP2 48 ------------------------------------------------------------
MsPBP1 54 ------------------------------------------------------------
MsPBP2 50 ------------------------------------------------------------
MsPBP3 49 ------------------------------------------------------------
MsOBP10 51 ------------------------------------------------------------
MsOBP21 68 ------------------------------------------------------------
MsOBP25 158 RFGNYNGKEDFPQSNEYGGHEMPGQGQYNNYYSTTPAPRRYKRERRPENSGQRSQYNPNN
MsOBP28 113 ---------------------------------------------------EKLLTSIPR
MsOBP26 60 ----------------------------------------------------------EA
MsOBP27 50 ------------------------------------------------------------
MsOBP23 44 ------------------------------------------------------------
MsOBP22 44 ------------------------------------------------------------
MsOBP24 57 ------------------------------------------------------------

MsOBP1 48 ------IDELKAA-KAAASADG--------IDNCFLGCVFKKAEVINAKGEFDLDNALTK
MsOBP2 48 ------SDDIESA-KTAGSADN--------IKPCFLGCVLKKAEILNAKGEYDSDKALTK
MsOBP3 50 ------KADIQAA-KEAGSADG--------IKPCFLGCVMKKTETLDDKGLFDAETALSK
MsOBP4 49 ------WEDIAKA-KETHTVSS--------LKPCFVGCIFKKFEIINDKGEYDLEANLDK
MsOBP7 47 ------EDDLNGV-VKTSDVTN--------IDSCYWGCYFTKMGVLNDKGQFDLNNFQTT
MsOBP8 42 ------MSELSEV-MSNGDVSV--------MKPCFWGCAFTKTGFLNDKGQYDVDSGLIG
MsOBP5 46 ------ASEILAV-MTSRDVTL--------VNPCLWSCCLKKGGFIDDKGQYVLNPGLTY
MsOBP6 44 ------NDQLRTV-VTSQDTKS--------VNSCFFACLFKRSKIMNDKGEFDVKTGLTY
MsOBP9 45 ------VADMTEL-RKKIMPDS-------EPIRCLFACVYKKTGMMNEKGELSVDGVNEM
MsOBP11 50 ------EDEVGDI-EKGKFIEN-------RNVMCYVACIYTMTQVVK-NNKLSYEAVIKQ
MsOBP13 49 ------EDQVKDV-DKGNFIEE-------KNFMCYVACVYKMGQTIK-GNTINHDMMLKQ
MsOBP12 46 ------EDQIGTI-DKGNFVED-------KKVMCYIACIFEMTNVIK-NGKLNYDASIRQ
MsOBP15 43 ------DDKIDPI-AEGVFIDE-------KEVKCYMACIMKMANTIK-NGKLNFDAAIKQ
MsOBP14 44 ------EDQVKNI-EQGDFNED-------PNIMCYVACVYKSLQVVK-NDKLDVGLISKQ
MsOBP16 48 ------EEDITNC-ENGIFKED-------TKLKCYMFCLMEEASLVDDDGTVDYDMLVSL
MsOBP17 46 ------VALVEQV-NAGADLMP------DAKLACYMKCTMETAGMMS-DGEVDIEAVLAL
MsOBP18 44 ------TEVLAEA-KKGHIVED-------ENLKKFTFCFFKKAGIVDSDGKLNVEVATAK
MsOBP19 40 ------EALVNKL-KTGDYKTES------EPLKKYALCMLMKSELMTKEGKFKKDVALAK
MsOBP20 42 ------DDNLNKM-KTNPFVEDG---G--EIFKKFIKCYLEKTGAITEDGKLNVDEALPK
MsGOBP1 44 ------EEKMEEF-FHFWRDDFK---FEHRELGCAIQCMSRHFNLLTDSSRMHHDNTEQF
MsGOBP2 48 ------AEILEEF-QHFWREDFE---VVHRELGCAIICMSNKFSLLQDDSRMHHVNMHDY
MsPBP1 54 ------DSVLTDF-NNFWKEGYE---FTNRQTGCAILCLSSKLELLDPEMKLHHGRAQEF
MsPBP2 50 ------EHIMQDM-YNFWREEYA---LVNRDLGCMVMCMAAKLDLIGDDQKMHHGKAEEF
MsPBP3 49 ------DQIMADL-YHFWKLDYA---LLSRDTGCAIICMSKKLDLMDASGRMHHGNAQEF
MsOBP10 51 ------DEDLKKY-QGGDDMN-----------PCFKKCMMQKLGLLDQEGKYDKATLHET
MsOBP21 68 ------MSYIQAM-NESGSFLDE---T-DKTPKCFIRCVFTNVGIVSEDGKQFNPARAAF
MsOBP25 218 HKITGYEDSFRSD-EKNTTENSS---KETDNNACALHCFLENLEMTAEDGMPDRYLVTHA
MsOBP28 122 PASPNLHNNINNN-NTSRTRRSEPLLNKPDSDQCLSQCVFANLQVVDSRGIPREAELWNK
MsOBP26 62 LDVIKEEHTMPAQ-RRRDKREVPFTHDEKRIAGCLLQCVYRKVKAVDGYGFPTLEGLVGL
MsOBP27 50 -------EDLKEC-FQMRGNPVT---------CENEVCIAKKKGFATDDDKLDYTKLEEV
MsOBP23 44 -----DPKHLAKC-SNPKLP----------GPCNDIQCVFEESGFLTDVNTLNKEAYKNH
MsOBP22 44 -------AVPAKC-AKYDDE------------CERLTCVFRESKWLD-GTAVDKAKVLAH
MsOBP24 57 ------DGDMERCGIEKASVDQSKNPPKPRIPCKESICLMQNANMLLANHSVDYEKLRTF

MsOBP1 93 LKGFVSNED-HFAKFEDI----GKKCASV-------NEKPVSDGDAGCERAALLTACFLE
MsOBP2 93 LKKFVPDET-KYAKYAEI----GKKCESV-------NEKAVSDGEAGCERGALLTACFLE
MsOBP3 95 LRTFVKSDE-DFAKFEEI----GKACMSV-------NEKSVSDGEAGCERAKLVLACFLE
MsOBP4 94 IKIFVKNED-LLTQLRDI----MKKCVSV-------NDESVSDGNAGCERAMLLAKCFAE
MsOBP7 92 MKKLMKDDE-DYDNLEKL----VKKCEPV-------KDETVTDGEAGCERGTLFAVCFVK
MsOBP8 87 VKKYMKDPL-GLEKLEQM----ARQCESV-------NDKVVSDGNAGCERGMLAAKCFLE
MsOBP5 91 VKNIVKSDQ-FYTFIEKS----AKQCESV-------KDKAG----SECELGALLAACIVE
MsOBP6 89 VRQVLPGRP-ETVVAESI----IKECESV-------KNTAVNDGEAGCERAALLVACLLE
MsOBP9 91 SRKYLA------------------------------------------------------
MsOBP11 95 VDIMFPAE------MRDAVKAAATFCKDT-----------TKKYKDLCEASYWTAKCMYD
MsOBP13 94 VEMMFPTE------MKAPVKAAIEHCRPV-----------AKKYKDVCEAAYWTAKCTYE
MsOBP12 91 IDLMYPPD------LKEGAKAAVDKCKDV-----------QKKYKDICEASFYVAKCMYE
MsOBP15 88 ADLLLPDE------VKEPAKEAILACKKA-----------ADGHKDICDASFHVTKCIYN
MsOBP14 89 IDALYPPE------LKEPTKKAVALCINS-----------QDNYNDLCSRVFHGAKCLYE
MsOBP16 94 IPDEYYER----------TTKMIFSCKH-----------LDTPDKDKCQRAFEVHRCSYG
MsOBP17 92 LPPELAAH----------KAPSLRACGT-------------VHGADHCDTAWKTQKCWQA
MsOBP18 90 LPPGVDK---------EDAKKVLEGCKS-------------KTGKDTADTVFEIFKCYHK
MsOBP19 87 VPNAADKP-----NVEKL----IDSCLAN-KGN------------TPHQTAWNYVKCYHE
MsOBP20 90 LGPNFAKK------IFEH-------CKT------HVETKGEEFVVVPTTTASDYSECFRQ
MsGOBP1 94 IQAFPNGE----VLARQM-VSLIHGCEK----------QFDHEE-DHCWRILHVAECFKQ
MsGOBP2 98 VKSFPNGE----ILSGKL-VELIHNCEK----------KFDSMT-DDCDRVVKVAACFKV
MsPBP1 104 AQKHGADE----AMAKQL-VDMLHSCMQ----------TTPDDANDPCLKTLKVVTCFKT
MsPBP2 100 AKSHGADD----ALAKQL-VGLIHECET----------THAGVE-DACSRTLEVAKCFRT
MsPBP3 99 ALKHGAGD----DVASKI-VTIIHDCEK----------KFERDD-DECLRVLEVAKCFR-
MsOBP10 93 MSQYGEDKE-KAQKIEDQ----IDSCFMA------NADNNGDDEEAIKKRVDVMFNCIKE
MsOBP21 117 IFAGERNGK-PMDDIGDM----TAACAAD-------RQE-----TCPCERSYQFLRCLMS
MsOBP25 274 ITKDVKDED-LRDFLQES----IEECFQI---------LDNENTEDKCEFSKNLLICLSE
MsOBP28 181 VQSAVTSQQ-SRSALHDQ----IRACFEE-------LQSEAEDN--GCSYSNKLERCLML
MsOBP26 121 YSDGVNERG-YFMAVLEA----SRECLMKNHDKF--SRTVPMDNGRNCDISFDIFECISD
MsOBP27 93 MTKEIDDK----DLLADM----IKNCVNGDLEKYGPPDFCEFMKMRHCISMQMLNHCPDW
MsOBP23 88 LKQWEENHAGWSVAVDKA----IKDCVDSD-PRQHLNYPCKAYDVFTCTGIAMLKKCPAA
MsOBP22 83 LDQYERDHAEWGPAVQFA----KTACLGP--------ELKAQGVFLNCPAYDV-THCILS
MsOBP24 111 VDIWADSNPEFTEAILEA----KKACAKDGGP--SGPPVCEQDRIFYCLTSNVLWNCKLR

MsOBP1 141 HK-GEMPLNF--------------------------------------------
MsOBP2 141 NR-ADIL-----------------------------------------------
MsOBP3 143 HK-ADIPF----------------------------------------------
MsOBP4 142 LK-SEILI----------------------------------------------
MsOBP7 140 ND-GDFI-----------------------------------------------
MsOBP8 135 KDNGQIVPSAL-------------------------------------------
MsOBP5 135 QMMKM-------------------------------------------------
MsOBP6 137 QTTKKARKPK--------------------------------------------
MsOBP9 ------------------------------------------------------
MsOBP11 138 YDAENFVFP---------------------------------------------
MsOBP13 137 FDPANFMFP---------------------------------------------
MsOBP12 134 FNPADFIFA---------------------------------------------
MsOBP15 131 QNPGIFYFP---------------------------------------------
MsOBP14 132 KDPACFIFP---------------------------------------------
MsOBP16 133 KDPDLYFLF---------------------------------------------
MsOBP17 129 ANKADYFLI---------------------------------------------
MsOBP18 128 GTKTHILLAGL-------------------------------------------
MsOBP19 125 KDPKHAIFV---------------------------------------------
MsOBP20 131 GVSNYIWNAKQEGFEPFTYEWQK-------------------------------
MsGOBP1 138 ACVQHGVAPTMEMMMTEFIMEAEAR-----------------------------
MsGOBP2 142 DAKAAGIAP--EVAMIEAVMEKY-------------------------------
MsPBP1 149 KIHELKWAPSMDLIVGEVLAEV--------------------------------
MsPBP2 144 KIHELKW-----------------------------------------------
MsPBP3 ------------------------------------------------------
MsOBP10 142 LKE---------------------------------------------------
MsOBP21 160 MEIEKYEKS---------------------------------------------
MsOBP25 320 KGRANCDDWKDDLKF---------------------------------------
MsOBP28 227 RFSDRQVDGKATTKKPTSTEQS--------------------------------
MsOBP26 174 RIGEYCGTAGL-------------------------------------------
MsOBP27 145 DDAGECSKLKGAVADCVKLFA---------------------------------
MsOBP23 143 AWKC--------------------------------------------------
MsOBP22 130 SFIKHATPTQWSSSASCSYPHAYAAACPVCPSDCFSAQVPIGSCNACYLQPRTP
MsOBP24 165 DFEDCRVLKAHMDECRPYYWKK----REEDEANAPTS-----------------

**Supplementary Figure 3.**

*M. separate* **adults**


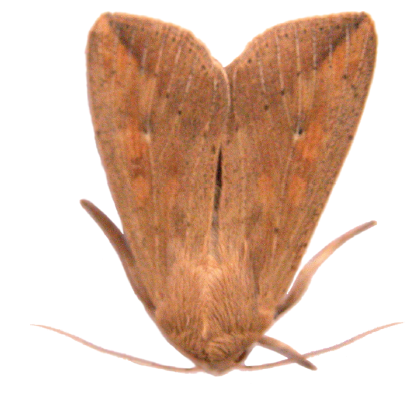


**Heads**

**Illumina HiSeq 2500**

**Paired-ends sequencing**

**27,208,038 raw reads**

**Trimmed**

**26,128,167 clean reads**

***De novo* assembly**

***Trinity***

**46,459 unigenes**

**Blastx**

**Functional annotation**

**（Nr, KOG, KO, GO）**

**SSR discovery**

***MISA***

**Circadian clock genes**

**Melanin synthesis genes**

**Non-receptor olfactory genes**

**Supplementary Table 1.**

| **Clean data** | **Number** |
| --- | --- |
| Clean reads | 26128167 |
| Clean bases | 3.27 G |
| Q20 | 0.9594 |
| GC content | 0.4602 |
| **Transcripts** | **Number** |
| Min length | 201 |
| Mean length | 783 |
| N50 | 1343 |
| Total Nucleotides | 49708735 |
| **Unigenes** | **Number** |
| 200-500 bp | 29222 |
| 500-1 kb | 8498 |
| 1k-2k bp | 5674 |
| >2k bp | 3065 |
| Min length | 201 |
| Mean length | 690 |
| Max length | 13245 |
| N50 | 1153 |
| Total | 46459 |
| Total Nucleotides | 32041796 |
| **Annotation in database** | **Number of unigenes** |
| Nr | 19973 |
| Nt | 6006 |
| KEGG | 7552 |
| Swiss-Prot | 12703 |
| PFAM | 13461 |
| GO | 14850 |
| KOG | 9256 |
| Annotated in all databases | 2552 |
| Annotated in at least one database | 22334 |
| Total unigenes | 46459 |

**Supplementary Table 2.**

| Genes | Known genes in NCBI | | | Transcriptomic data | | Identities |
| --- | --- | --- | --- | --- | --- | --- |
|  | Accession no. | Length (bp) | Complete | Unigene ID | Length (bp) |  |
| adipokinetic hormone 1 precursor | KP979739 | 449 | Yes | c26248_g1 | 454 | 100 |
| juvenile hormone epoxide hydrolase | KM926340 | 1632 | Yes | c29442_g1 | 1504 | 96 |
| juvenile hormone acid methyltransferase | KM926339 | 1730 | Yes | c26750_g1 | 1655 | 96 |
| vacuolar ATP synthase subunit H | KC683729 | 1807 | Yes | c29936_g1 | 1990 | 99 |
| vitellogenin protein | KF501044 | 5699 | Yes | c32394_g1 | 5635 | 98 |
| UV opsin | KF539458 | 1140 | Yes | c30579_g1 | 3153 | 98 |
| blue opsin | KF539428 | 1149 | Yes | c28839_g1 | 1617 | 97 |
| long-wavelength opsin | KF539446 | 1146 | Yes | c22414_g1 | 1810 | 99 |
| olfactory receptor 2 | AB263111 | 1422 | Yes | c31350_g2 | 2867 | 97 |
| olfactory receptor 3 | AB263205 | 1275 | Yes | c25819_g1 | 1560 | 97 |
| tyrosine hydroxylase | AB274834 | 2273 | Yes | c17613_g1 | 2280 | 99 |
| allatotropin | DQ208707 | 411 | Yes | c24989_g1 | 1486 | 100 |
| pheromone binding protein | AB263112 | 513 | Yes | c26255_g1 | 1003 | 97 |
| cyclophilin A | HM113489 | 826 | Yes | c17951_g1 | 804 | 99 |
| glyceraldehyde-3-phosphate dehydrogenase | HM055756 | 1317 | Yes | c24931_g1 | 1359 | 99 |
| allatostatin neuropeptide precursor | JQ669383 | 902 | Yes | c26533_g1 | 1211 | 96 |
| octopamine receptor | JF502068 | 1397 | Yes | c21896_g2 | 1880 | 97 |
| inhibitor of apoptosis protein | AB778567 | 1642 | Yes | c20809_g1 | 1836 | 99 |
| dopa decarboxylase | AB072300 | 3230 | Yes | c31937_g1 | 3270 | 95 |
| vacuolar V-type H(+)-ATPase B subunit | KF896233 | 2188 | Yes | c28260_g1 | 2340 | 97 |

**>**c26248_g1

GACTTCTGGATCACATATCCACCACTCTTCCGTCTACCAAGTATCAAGATTAACACAACAACCACTCTTCCATCTATCAAGTATCAAGACCACTACAACACACTCCAAGATGAACAAGATTTTCTTCGTGCTTCTCTTCGTGGCTTGCTTCTGCCTCTTCGCCGAAGCACAGCTGACGTTCACTTCCAGCTGGGGCGGCGGGAAGCGCTCGGGCGTTGCCCCTATCTCCTGCAAGAATGAAGAAGCTGTCTCCACGATCTTCAAGTTGATTCAGAACGAAGCTGAAAGGTTCATCATCTGCCAGCAGAAACCTTGAAGATTAACGACGAATGATGATTTTCTGATGACCTGAAGACGATACTACAATGTTATAATGGAGAATTTGTTTAAGATATTGTTGTTTATTTATATTTTTTCTTGAATTTACAATAAAATTATGATTAACTGAAAAAAA

>c29442_g1

TTTTTTTTCAAAACCATAAAAAAAAAACTTTAATAACCCCTAACCAAATCACAGTTCAGTCTTCTGATTAGTCTGATGCCACTCTTTAAACGCTTTCACCGCCTTGAACACATCTGCAGCGAAGACCTGAGGCAGTTCGAACGCCAGGAAATGCCCTCCATCATCCAAGACTGTGGCGTTTATCAAGTTCTTGTACTTCCTGTTCAAGAGGCTTGGAGGTTGATACATCAGCTCATTCTTCGCTTGAAGGGCCCAAGTCGGGACCTGCGTGGTGATCTGGTCCAAATTATATTCCCTTATTTTGTTCGAGAAGTTTTCGGCGTAGAACCTCATGGAAGTAGTAATAGAGTTAGTAGACCAGTAGATCATTAGGTTGTCAATGAGTTGGTCCTTAGTAAACCTTAAAGCCAGTCCACCGTCAGCTTTGCCCTTGTTGTCTTTATTAGTCCATGTGGAGAATTTCTCCAGGATGTATGCTAAGAGACCAGCTGGAGAATCATTCAAAGGAACACCAACAGTATCAGGTTTAGTGGCCTGGATGTGCATGTAGCCGAATTCTTCCATCACGTAGGCAAAGTAAGTCGACAATGGGTACATTCTGCTTGCTAGATGTTCCTCGACTATGAGGGATGGGAAGAAAGCGCCGATGAACCATCTTATTGTTGAGCAGGTGTGCTGAGACAGAAGCATATTGGAGTGATGTCCCAGGATGTCTTCAGGGAAGATGGTGGACATAACCGACACGATACCTGCGCCCCAGTCACCTCCCTGAACGTAGAACTTCTTGTGGCCCAAACGGTTCATCAGAGTTCTGAAGATGACCGCTGTTTGAGGCATACCCAGGCCGGGTCTAACTGCAGGGTCAGAGAATCCATATCCAGGTATACTGGGAATGATCAGCTCAAACACGAAGTTGTAACCTGGCGTCTGTTTCGTGAGCAGAGGGATGGCCTCGTAGAATTCTCTCACAGAACCTGGCCAGCCGTGTATCATGAGGAGCGGAACCGTCTGCACGTCTTTGGGAACCTGAGGCTTAATATGTATGAAATGAATATCCAGTCCTTGGATATTGGTCTTGAAGTGAGGGAACTTGTTGAGGAAGGCCTCCCTCTCAACGAAGTTGTACTTATCAGACCAGTAGTTCAGCCAGCTGTCGATCTGAGCGGTGTTGAACCCGTACTCGAAGGCGACACCTTCGAGAGGTGGTGTGAACTTACGGTGGTTCTTTAGACGGAATTTGAGGTCTTTAATCATCGCTTCATCGAATTTGACTTTGAAGGGTCTGATGCTGTTGTCAGTCTTTCCTTTAAGCGCTGTTGGCCCCCACCACTCTTCACTGTCGAACTTGGGTATGGTTTGCTTGCAGCAGCCGTTGAAGTAATACCATACTGCCACGCCCAAGGCGGCCACCAACACCACTTTTACCAGGAAACCCATGATGATGTTACCACGTCTGCCCGTGTTCACACAACACACTGGACTCCGGTCATGTAATCATTTGTTT

>c26750_g1

ATTGAATATTGCCGTTACCCCTTACAACTATTATTCTAAAAATCTATTTAATATAAATAAATAACAACTAATATAAAGATTATAATTATATAAAATTTAAATACCACCTATTTACATAAAATGACGATAGATAATATTATCATCACCATCTAAAAAAAAGAATCACATCCTTATTTACTAGCCTTAGACACGTATCAATCGCCACCACCATAAAACAGAAAATCATAGAATCAGCACAGAAATACAATCAGAGATCAACATGATTTTTTTCGTTATTACGATTATTTTGAAACTCTAATTAATTAATTGACTTGAGCTTCCGAATATGCGAACCAAGTTAATAACTTGTTTTTTTTGAAGATTGTAATATCATGGTCCACCATGATATTACAATCTTATTTTCTACCAAAACAAGAGAAGACTACGAGTATATAAATGTGCTACGTGATGGAATTGGCTCCTTTCAGACAATATTCTTTTTCAGCTTTTAGGTACTTTTAGCAATAATAATGTAAACAAAATGATTATACTCTGTTGCTATCCGGCTCGAAGGACCATGTTTATCACTATGGATATAATATATACAACATTACTTCTCCTATAGGCCAATTTTCAAGCATGTAAAATAAATAGACAGGAAGTAATCAAAGTAGGTAAACAAATGCACTGAAAGTACAGAGTTTTCGGTTCCAGCATAGATGTTCAGACCTGCTTGTTTAATTCTTCCAGTAGTTTATCCTGTGAGGCTGGTTTGCGAGCATACACCACTAGTAGACGATAGCGGAACTGAACGCTTGTTTCATAATTATTGTTGAACTTGGTCATAATCTGCAGTTCTTTTAGTATGTCCAAGTAGTCCGACATGAAATCATCGAACTTATCTTCAGGGATTTTGAACGGATTCACCGCTGTGAAGGCACTTCGCAACACATGTAAGTCTTCAAACACGTACACTGAGTTCTTACACTGCACATCGTAGTCAACGAAACCAATCTCTTCCAGCATTTTCTCGACTTCTTTTTCCGGGTCCTTAGAGTCGTGATAGGGCGACACGTATCGGTCGACGTTCTGCAGCCACTTGCTCCACTTGTTGTTGCGCGCCAGCACCCTGTAGACGTCGAACACAGGCGCGCCCGCGAGGAAGATCATGAAGCACTCCCCGTCTTCGTTCAGCAGGTTGTAGATATTGGTGAATGCTCGTTTTTGATTTATAATCCAGTGCAAAGCGTAGAATGAAAACACGTGGTCGAATTTTCCCTTCATCCCTTCAGGTAGGTCCCCTTCTATGTCGAGAACGGTGAAGGAAGTCTGTTCATTGCAGTGGTGATTATTGGCGAAGTTCACCATCTTCTCACTGATGTCGCAGCCGAGGAGTTTGAAGTCAGTGGGGATGTACTTCTTCAGCATGTTGGTGACGCTTCCATCTCCGCAGCCGATGTCTAAGATATTGTTATTGTTTTTCTTCCACTTCATTTTCGACGCGTTTTCTTCTAAACACAAAAGAGCATCCCTCTTCTGAAGGCTGTTGCATTTTTCGTATAAAACAGCGTTATTCATGATTTTTTTTTTGTTGCAGGGATTTTCAGAGGATTTTTGTGTTTATGATTTCTGTTTAGTATTTTTGGGAGCGCACGCGTGGTGTGGGAGCGCTGTTT

>c29936_g1

TTTTAACATTATGATGAACTTTATTCACAATACTGATTGCTGATGTAGACAGTTATTACATCACAAACCACTAAAACTCTATTAATTTCAAATTATTGAATACATAATATCTTTTTTAATAATCACATTCATATTATTTGAAATCGGTAATGTAATAAACCCTTAAAGGAATGTGTACTTACACCAAACATTCTATAAATTTAGTGTTACTTTTATTGTTTAACGTAGCTACATAGATACACTCAATTTACATTTTTATATGATTTAACAGAGAATACAGATATTAGTGATGTCAGAACGGAACAAAGGCACATTATTTACATTATAATTTATGTAATGCAGCTATAGATCTGTAACAAGCCTGGTTTCCTTTAAGACAACAACATAACAACAAATTAAATATTTCATTTATTTTATAAATACAACAATTTTAGGCCTTAGCTCCGACTACAGTGCCCGCCTGCTTGTCGATTTGTTCTTTTTCCAGTTGCTTGCCAAGGTACTCCCAGTTGTGAACCATCAGTTTCTGGACGGCGAGGAGGGCCTCGTATCGCACGTTGGGGTCTTCGTGGCTGAGGAGGTGCATCACGCGCTGCTTGCCGCCGAGCTGCTCGATGATGTGCTTGCCCCTGGGGTAGTGGCGCACGTACTCGCCGACGTCGTAGCAGGCCACGGCCAGCACGACGGGGTCCTTGCTCTTCTCCAGCAGGTGCACCAGCGTGCGCAGCAGCTCCTGGCCGCGCTCGTTCAGGCGGATCGCGTTCTCGCGCCAGAACTTGGCCGACTTGTGCACCGGAGACCACTCCAGACGGCCGCTCTTGACTTCGGTGGCGTACTGGTCGAACGAGCTGAGGTCTTGCACAGATGTCTGCAGACGCTCATTGAGGAAGTCCACGTCATTCATGATGTCTTCATCGTCGGAACGCTTCTGCTCCAGGATCGAAAGCTGCTTCAGGACTTTGCACTGAACCATGGCGATGCAGTGCTCCTTCGAGACCTGTTGGTTTTCAGGCTTTTCGATAAGGTTCCTGAAGACAGCGAGCACGATGCGCGTGACCTTCTCCTTGACGGAGTCGCTGAGGATGTCGGCGAGGATGGGGATAGCGTTGAACTTGTTCATCTTCTCGGCCAGCAGAGGGTTGAAAGTCAGCACCCACAGGCAGAACGTCAGCTGGTATTGCACCTGGAAGTTGACCCTGGAGGCCAAGATAGCCAGCAGCGTGGAGATGCCGTCGACGGTGAGGAACGCGAAGCGGTACTCATCGACGCGCAGCATCATCTGCAGGCAGCGCGCCACAGACTGCACGTATTCGTTGTTATTCAACTTAAGTTGGTCCTTGAGCCAGGAGAGGTAGAACAGTAGGTCACTCCTCTCCATCACAGTGGTATGCCAGCAGGCCAACTTCGCAATAATACGAGCTGTCATGTGCTGAACAAACTCATCCTGACGGTTCAGCAAATTCAGGAATGGCTGCCAAGCATTTCCAGTCCTACCTTCACGGAAGATCTTGACCCTATTCTTGTCTTCAGAAAGGATGTCATCAATCATAACAAGCAGGTACTGGATAGTGTTGTCCTTGCTGATGTGTGTTAGCATGTTCAAGAAGACCTCGGCACACAGCTCCGGGTTCTTGTCAGGTAAATCTTTATGTTGACCACGGTCCAAATTCACAATAAAGTCGTGGTCACGTTGCGTGATCATCTGCGACTGCAGATAAGATGACCAGGTGATGTTTTCTTGACGAATTTCACTGGCACGGATTTGCAACGCGCTGGTGGCAGCAATCATATCGATTTTTTCGTCACCCAGCGGCGGCATAAGCTGACTAACATTTCCATCTCCTATATTAGCCATTTTGGCTACAGAATTGATAATCACACTTGTTATCCCCAAAGAAGAATCGAAAAACTTTATATTTAGGAAAAACTCAGAATGAAGAGACGAAGACAGCCTCAGCTGAGAGGTCTTGTGACTACGAAGAGTGACGTT

>c32394_g1

TTTCACTTGCTCTTTACAAACTATTCTACTGCAATAATAATACATTATAGTCTTTATACATAAATACAAAGAGGTACATAAAATACTTATATGAAATAAATATTTTTATACATAAATAAATGTCATAGTAATAATTACATAATACGACAATAAATTTCGAAATATATAAATATTATATTGCCAAAGTAGATATGAACACATAGTTTATTATTAATAAAATATTCGTGGTCCAAGTTCATGCCATTATTTTCTACTAATATCTAAGTAGTTAATTGAATTAGGTGGTAGGTGGAAGGGTTCTTACGCCTTGCACGACGTGGGTACTCTGTACTGTTCTACTCTCGGAACTCCATCAACCTGAGGGTTCTGGCCCTGGTGAATCTGGCTTCTGAAAGATCTGAACTGCTCGTCAGTCTTTGGCCGGCAGATGACCTGAGCGGCTTGGACCTTGTAGCCTTCTCCATGGCAATGAGACGGGCAAGACGGCAGAGGGATAGTGGTAATGCAGATCTCCCCATGGTTCTCATGATACTGGATTTGCTGCTGCACCTGGCACTGTCCGCGAACCGTCAAACCGTGGCTCCTGGTCCTGTAAACATTCTGGGAGTCCCAGTCCTGGTCTCTCTGCATGATGGCTCTGTTCCATTCTTCATCGGAGCGGAGAATAGCGGTGTATTTAGGAATTCGCTGGTACGCCTTTTGCTGAGCTTCCATCTTAAGGCCTTGTGTCTTAGGGTCGCTGTCTTCGACGTCAAGGGCAAAGGATGCACCATAGTGTTCAGGCAGGTCCACCAAACCATGAGGAGTCTGGTAGTCGTTGCGTGGCTCACCAGTGTTCTGGCCGCAAATGCCTCTGGTGGTGGAACGGTATTCTTGGGTGGTAAGCACCAGGCGCTGACCGTCGTACATGAGGCGGAGACGTCCGTCGCGGATGTTCAACATCAGGATACCTTCGGCTTGGGTGTAGTACTGCAGAAGCGGGACCTCAGCAACATCATCCCAGTAGATGGTCAAGTCTCCTTCAGAGATCTTCTTGCTGTTGGTCTTTACTTCAACGATAGCTTTGTGAGACTTTGACGGTAAAATCTCGATTTCCAGGTCCTTGCCAGTTTCGGTTTTGTAAGAGATGTAGATCTGCTGCTGCAGCTGGGTGGGTCTCCTCGCGAGGATGACCAGCTCGTCCCACTTGCCGCGGGCCTTGTTGTACTCCTCCTGCATCACCACGTGCCAGGAACGGGACAGCTCGTAGTCGTAGGTGCGGTTGCTGAAGGTCCTCACTTTGGTGCCGTCAATGGTACAGAAAGGCTGGTACTGGTAGCTGGTCAGGTAGTTGACGATGCGCTCAACCGTGCTGAAGGGCGTGTAGACCGACACAATGTAGGGAGTGATCATGGGAATCGGCAAGTTCTCGACACGAACTAAACCGGATGGTGAAGTCATCTCGAAACGAAGGGTGTTCTCGCCGTAGAAAGTCTCAACCTCCAACTGCAGTTTACCATCAGCAACCCTCTTCAATGGGTTCACTTCCGTGTTCCAGCTCAGTTGCTTCAGAATCTGGTTCACTTGAGCAGCCCAGTACATGTACATGGGGCTCATGTTCTTGTAGGTCACTGACGCCTTGAAGTTATCAGGGGTATGGGCCATAATCAGCATCTTGAGAGAAGTAGCCTGGTATAGGTTGCCATTAGCAATATCCTGCTGCACCAACTTAGCCAAAGGATGGTTCTTCAGGTATTCGGTGAACTTCTGAGAGCGTTCAGTGTTTCCTTGGATGTGAATATTTCCATTCTGACCAAACTTGATGTCGGCTTCGTAAGTCATCTTCATGTCCTTCTGCAGAGCTTCCAAGAAGTTCAGTGGCAATATTTCGGGCTTTGTAACCTTGACGACAGCGTTGATCTGTTCGTTACCCAGCTTGACTGAGTTTCTTCCAGCGAAGAGGACATATTGGATTTTGCGGTCGATCGGGCTGTTAGCGATAGCGGCAGTCAGCACGTATTCCTGTTTCAGGGGTCCCTCGAATGTGGCGCTAACATCAACAACTTGTACTTTAGCATTGTTGATTCCAGATGCAACACGCTTGACCATCTCCTCACGGCGGACTCCGCTGTTGGGCGTCACATCGTTCCTGTTGTCAGCTTGTCCCAGCTCTCCGCTTTGTTTCTGGTTGAAGAATGTATCGTGAGCAGCAGTAAAGGTGACAACCTTGTTCTGCGACTGTTTTCCAAGATATCTGAAGTTGTAATGCGTCAGAGCGATGTCACGGGCACCTAATAAGTCACCAATGTTGTACAGGGCCTGAACCAGGTTTCCGGCGTTTCTGAAGTCCGTGGAGTAGGAGTATCCTTGCAGTTGGAAGACAGTGCCAGCTTGTTGGCCGAACTTCATGTCAGTGGACAGTACCTTCCTTGAGCGTTCTACTATTTTGGTGGCAGGGTCCTGAGAGTAGGGCACTAAAGTATCCTTCTTCTGGTAAGTAGTGAACGGCCAAACGCTGTAATGAGCGATGGTGTAGTCCTGGTCAGGGCGCAGAGGTTCCACCTTCATTTTAGCTAGTCCAGATTCCATCTGGAAGCTCAGCTTGACAGGTACGTTAATCTGGTATTTCTTGACGACTCCAGCAATAGCCAGCTGGTTAGTAAGGGTGTCCATGAAACCAACGGTTCCATCGATGTTTCTGGCAAGCGTAAGTTGGAGTTCCTTATCCATGAAAGAGGACAGATTGTTGCGGTTCTTGGGGTCACGGTCGATCTTGCCCTTAGCCTTGCTCTGGATGTGCATAACGACAGGCTCCTTGTATTTGTAGATGAAAGGCATACCTGAGGCGATGGGGAACATAACGGAAACCTGGTTGGAGTTGAACACTTTGGTGTAGTGCTTCTCAACACCCTGTTCTACCTCCTTCATGTACTCCATGATGTCTTGTACCAGCCTGATAAGGTCTCCTTCGTCGAAACTGTAGAATCTCTCCTGGTTGAAAATGTCGTAGAAGAAAGAGCCCTCGAGTGGCCTTTGAGATTCACGCTTGATGTTCAGCATTTCCGTAATTCTTTGGGCATTGAATTTGTGGTTGGCTTCAGACCTCTGAGGCTCAAACAGCATTGTCTTGATGAAATCAACGATTTCCTGCACATCGGAGAATGAAGCGCCAATCATGTTTTCAAGGGCCCTTCCTCCGAGTTTGTTCATCCAGGAGTGTCTCTGGTACTGAGGCAGAACACTGCTTTCACCACCAATGTAAGATGCTACTTGGAAGTTCTCCAGTTCTCCATCATTAACATAGTTGTTGATGTAGAATTTGTTAGAGTAGTGCATACCAAGGTTCTCTCGTGTTACGATCTCCTTGACAGACTGAGCTGCCCTTGACAGGTGCCAAAAGCGAGGTTCCTTCAAGCTGGCAGCGGTTAAGATGCCATTCTTGAGTACAGCGCGAACCTGGATACTGGGATCATCGTGGGTCATCTGGGCCATAACTTGCATCATCTCAGCGGTCGGGTGAGCCATGAATAAGTTCAGGATAGCAGCCACTCGCACTTCGTACGGCTCGGCAGTGTTCCTCATGATGCTGTAGAGCACAGCACGCACGTATTTATCCTTCTGGTTCGAGAGATGGCGAAGATTTACAATCATTTGAACGCGGAGGTGTGTGGACACTTCAATACGACCCTCCAGGTAAGGAGAGAATACGTATAAGATCTCAGGGTGTCCTAAGTTGCCAATAGCCTTAATGTACACCAGAGCCCTGCTCCATTCTTTAGCCTCAATAGACTGTTTCAACAATTCGGAGAGACGAGGAAGGATATTGTCCAGCACGAATCTGTCGTGTCTGCGGGCAAGACGGCCATACATGTGAGTGGGGTAGTAAGAGTGAGCAGTTTCGTTGTTGACTTGACCCATGCGAATGAATTCCGTTGCAGCGATCAAAGCACTAGTGTTAAGGCGTTCCTGCTGTTGGACCTCTGTGCTCATGGCAAGCTCGAAGAACTGAGCCATAATTTCCTTGGTGGGGTAACGAAGGGTACGTGCCAAAGTGGACAGCACCTGAGCGGCTTCCTCACCTTGTAACTTCTTGCTCACAATCCAAATCTTAATTTGCATAAAGGCCGGGGGTGTGCCAGCTTGTACGACAACATCACGGAAGATCATCCACATATCAGTTTTAACACTGTTGTTTGAATTTCTGCCGACTTCAATGCCACGGCTGATCTGGGCGAGCTGTTCAGTGCTCATGGAAGCAACAATGCGGACGAGGATGTTAAACTTGGATAAAAAGTCAGCTTTAGGCATGTTGTTGGGGTTTTGCAGTTGTTGAGCAATGTCCTGGAGCAGCTTCTGGACGTTCAACACGTTTTGTTTCTTATCACCACGCGGTAGAGCACTCATGTACAGAGCAGCGTATGCTGGCTCGTTCATCCTGGGAACGTCATCGTTGATGTAAGCTGATGAAGAGTCACTGGAACTCGACGACCTCCAGTCCTTGTTGAACATTCTCTCTTGTTGAACATCAGAACGGCGCACTCTGCTTCTCAGACCTTCCAATTGCACATTTATGTGCTCATGGGATTCAGCAGACTCGCCTGACTTGGAAGACTGGTCGTGGTAAGCCATTTGTTTGGTGCTCATAGCGTACAGAAGAGTGTGGATTTGACGGCTGCCTTCAGGTTTTTGCCATTCGGCACCACTATCTTGTTCAACGGACATTAAGTTCAAGCTTACGTAGCTGTGTACCTGCGCTTTCTGCTGGCCATATAAGTGGGGGTGCACGGTCACGAAACTCGTGGTTTCAGCCTTGTAGATAGGACCCTGCTTACCAGCAAGAATGCGGGAGGTGGCAGAGTGCGAGATGAATTGCTTCTCTTCATTGCTGTGTGCGGTTCCTGTCCATTCAAAACCTTCGGGTACTCCGAAGTGGTAAGCGACACGGTGGTGGCAGTGGCCGTAGTTCTTGCTCTTGGTGATCTCCATGGGGTCTTCTTCTGAAACGAACAACGGCAGCTCTCGTCGCCATTCAGCGGCCACAGGAGAGACTGTGTACATGGTTTCGCAGTCACCAGTGACGTCAGCCTCCATCTTTTTGAAAAGACCCTGCTGGCGTTCCCTGTCGAAGTTATTCGGTAGGTTGCGCACATGGTGGTAAGCAGATAAGTCGACTTGCAGCGCGCTGATCAGACCCTTCAGCAAGTTCTCGTGAGAGAGCTGGAAGACTGATGGCAGATTGAGGCCGAGGACGCGACCACCTTCAACGGAGATCTCGAAAGGTTTGTCCAAGTTCTGCACAGTTTCGTACTTGAGGTCCGAAGGGATGGCCATGTTGTTGGGCAGCTGCTGGTGGAACTGGGCCCGTTGGGGGTTTTCCAGCCTAGCCTGAAGGCGGCCTGGGGACACGACACGGAGGATGAACTTGGCCCTGAAGGCGTTGCCAGTGCTGGCGCCTTCCTGAAGCCGGGCCAGGGTGTGGGTGTTAACGTCATAGCGGTATAGTCTTCCAGTTTGCCAGGGCCATTGGTTATCCAACTGCTGTTCGCTCAGTCTCCCTGAGGAGACGACGGCGATAAACGCCGCCAATACCAACAACTTCATATCTAAGTCCTGAGTGCTGCTGGTACTTGATGGTTACTGGAGAAGTGTACTTCTC

>c30579_g1

TCTTCAGGTAAATCATATTCTTCCGGGCTCAGTTTATCACTAAAGCTAGAGACGTAAACATGTTCACACTACTTGTGTTGTGCGTCAGCTGTGCCGCGTTGAGTGGAGCTCTGAAGTTGGTAGATGATATCCCTTTACAGCTAACTGGGTTAAATGGAGTTCAGAAGATTGCTGATGATAACCCGGATTTACCTGAAGAATATTCAGAGTGTAATGAACCCAATGAAGAGTTAGATCATTGCGTCCAGCCGTGTGGGGGTGAAGATTCTTGTTATGATCGTTATTCGTACGCCTGCCTAGCCGTAGTCCAATCTTGCAGACCCGCGTGCGTATGTAAGGAAGGTTTCATCAGAAAGACTCTGGGTCGTGAATGTATACCAAAAGAAGAATGTGGGGGTCCCTGTGAGATTTACACACAGTATACAGACTGCAAACAAAGCTCCAAGGAATCCTGCTCTTCAATCACGAATCCACCAAAAGCCGATGAAGCCTGCAAACCAGGATGCACTTGCTGGGACGGATATCATAGAGAAAATGACACTGAATTATGTGAACATCGTTGTCGTTGTCCTGGAATGAGAGAGTCGCCAGATTGCGAGTCTTGGAGAAGAGTTTATGAGTAGGGCTGCCATCACCTAAATCGCCGACGCCGGACAAAAACTTCAAAAACCCCGGACATTTGGATCAAAATTCAGTTTTTTCCCCGGACACCCAGAAAAAATTATTAAAATAAAAATAAAATATATTATTTAATATAATCATGATTTTTGGTTTTTAGATTTATCGCTTATCTTTTAATTAATCGGGAATGCCCCTGACACTAAAAGCGTCCGGGTTTCACTCGGACACTTTTTGACAACCCCGCCCGGACGCTCCCCGGACGGGGCTCTAAACTAGGACAAATCCGGGGAAACCCGGACGGATGGTAGCCCTAGGTACACCGATAATACGTTTTATATAATATTTTATATAATATATTAAACATCCGCATCTGCATCTGCATCCGCGGATGTGAGCCTTCAAAACTCCGCATCCGCATTCGCATCCGCGGATGTGAAAAAATCAGCATCCACAACATCCCTGGTTTCCATGTTTGATGCATCGAATTAAAAACCTTAATAGGTAAATAGGATGTTTGATGTATCGTGTGCCAAATTGAAATGTTCGACTGGAGGTGTCATCTGCTATCCTTTCACACCCCTAAACACTCATGTCCTGTGGATTTTTTTCATCATTTTTTTTTTGGTTTATATAAAAAGGACTCAAATTGTCCATTCAAATACGAATATTGAAAAATGTATAAGGTGTAGGCGTGGTGACACCTTGCAGGAGTGGCGGGCGGTTGCCTCTTTCTGCGAAGCAGTAATGCTACCGAAGAAGGTGGCCCCGTGGCTCTGGAATGAAGGAAGCCAAAAGAAGTTGCGAGAATAGGTAATCACTATTTTCCAGTCATACCGCATGAGCACCGCTTAGCGTTCGAATTCAGATAGGTACTTTATTTTCATAATAAAATAAGATCGTTACAAAATAAAATAAACAATATCTTTGACTTCATCATTTTGTATTGCATGTAAATAATCGAGTTATATTTTTATAATAATATAAAGTTAATAATAAGTCATATTCCAGTCCGTTAAACAAGCCGTTGTCGTCGTATCGAAGATCTTATCCATGTTAAATATATTATGGAATAGGGTAGAGCTCTGAGCAAAAAGGTATTAAATTAACAACATTAGAGTAGACTATCATTATGTCCCAAAGGTGTCCTCGTCAAGATTTAGACAGCTTGAAATCTTTATCTATACACAACACTGTCTCGACACCTAATGGATTATGGTTTTCATAAATAAGCACAAAAATAATACTACTTAGTTGTCCTTTCCTCTTGCCTAGCAGTATGATTCTCATTTTGAGCTTAAATTAAAATAGTGTAGAGGATGGTAGGCAGTGTTTAGGCAGGTGCTGCGGGTGGAGCGCTGGACACGGTGTTGCTGGTGGCAGTCGACACGCTGTCATCCGGCTCTTGGATCTGCAACCACGGCATGCGACGCTGCAGCTCTTGTCTGTACTTCGGATGACTGATTGCATAAACCCAGGGATCTACGCAGGCCACAGCTTTGCATGCTACGGCTGGAATCATTGTTACCCCAGGCGTAAGAAGTTGTTGGTTTCCAAAAGCGCCTATTAGCGCCATGACCCCGTAAGGAGTCCATGACGCTACGAACAAGAAGCACACAGTGAGAGCGGCCTTGGCTATCCGGATCTCCGCTGATTCTGCGCTCGCATTTTGGTTAGCTCGAAGTGATTCCACATTCATCTTTTTGGCTTGTTCCCTCAAAGCAGCTTCATGTGCAAACACCTGTTTCACGATTCCACTGTAGAAATATATGATCATCGACATGGGGAAGACGTAACTGCAGGCGAAAATGCACGCGACGAACAATTTAGTGTCGAAGGTATTCGTCAAGTAATCGAACGTGCAAGACGTCAAATAACCCTCAGGAACATATCTTCCCCAGATTTTGAAGAGCGGTAGTAGGGCCCATGGTGTACTGTAGATCCAGACAAATGCTATCATCAGTAGAACTTTGCCGCGCGACAATCGCCCGTCCAAAGGACGAGTGATTGTAGAATGCCTGTCATAAGCGATACAGGCATTGGTCATGCCTGCGCCGATGCCACTATACGCGCCCATCAACGCGAACAGTTGACAGCCGAAAGTTCCGCTGGCGAAACCTCGCATCGCACTATTGTATATAAATATAGGGGCTTTTGCCATCATTATGAAATCTAAAATAGCTAACTGTAGAATTAAAAGGTTGCTCGACGTTCTTAAACTCTTTGTCGTTGAAAATATAAAAATAACTAGTCCATTGCCCACTAAAGCGGCTGCAGTAAAAAAGATGTAAAGCAGCGCAAGCGCCGTGTGCGCGCTGGCCGGGGGCGCCGGGAACGACAGCCAGTGCTCCGGCACCGCGGCCAGCTCGTCGCCCGTCAGGCCCTCGCCCAGCATCTCCACGCCACTCGGCCCTCCCGACTTGAAAGGTGCAAAGTGTGCATCATAGTAGGAATTTTCTGTTTGATTATACATGTTGAATGAATGTTGGGAAGGAGTGAGCAGTCCTCGGTAGTGTGCCTGACTGCCCACTGCAC

>c28839_g1

CGACTGGATGTCGGACTACTCACTTCGTCATAAAATATTTAACTTCATAAATAAAACCTTTGTTCCATTCAACCCATGAAACAATAGTAAAACAAGTATAATTTCCCGCCATTTTGTTCAAAAACGCGCACAAGATGGCGACGAACTACACGGAAGATATCGGCCCCATGGCTTATCCACTAAAAATGGTGTCCAAGGAGGTGGTCGAGCACATGCTAGGCTGGAACATCCCCGAGGAGCATCAGGACCTGGTTCACGACCACTGGAGGAACTTCCCAGCTGTCAACAAGTACTATCACTACGTCCTGGCACTTATCTACACCATGCTGATGGTCACATCCTTCTTGGGTAATGGCATTGTCATCTGGATTTTCGGCACCTCAAAATCGCTGCGCAGTCCAAGCAACATGTTCGTCATCAACCTGGCGGTGTTCGACATGATGATGATGCTGGAGATGCCGCTCCTCATCATGAACTCCTTCTACCAGCGCATGGTGGGCTACCAGCTCGGCTGTGACATATACGCCGTGTTGGGCTCCCTGTCTGGAATCGGAGGGGCTATCACGAATGCTGTTATTGCTTTTGATAGATACAAGACCATCTCCTGCCCACTGGACGGAAGAATAAACAGAGTGCAAGCGTTCATACTGATTGCCTTCACATGGTTCTGGGCGCTTCCCTTCACCATCCTGCCTGCCCTCAAGGTCTGGGGACGATTTGTACCTGAGGGCTTCCTGACCACCTGCTCCTTCGACTACTTCACTGACGACCAGGATACGAAGGTGTTCGTCGTCTGCATCTTCGTGTGGAGCTACGCCATCCCCATGACTCTCATCTGCTACTTCTACTCGCAACTGTTTGGTGCTGTACGCATGCACGAACGCATGCTCTCAGACCAGGCTAAGAAGATGAACGTGAAGTCGCTAGCCGCCAACAAGGAGGACGCCAGCCGCAGCGTCGAGATCAGGATCGCGAAGGTCGCCTTCACTATCTTCTTCCTCTTCGTCTGTGCGTGGACTCCGTATGCCTTCGTGACCATGACCGGCGCTTTTGGTGATAGGAACCTCTTGACTCCCATAGCGACGATGGTGCCTGCTGTCTGCTGCAAAGTAGTTTCCTGCATAGACCCATGGGTGTACGCCATTAATCATCCCAGATACAGGGCGGAACTCCAGAAGCGTCTCCCCTGGATGGGAGTCCGCGAATCTGACCCCGACTCCGTATCAACGACCACCAGCGTCGCCACCGCGCAGTCTACTGCCCAACCCGCTGCAGAAGCCTAAACCCTCACAGGAATACCCACCAATATTGCTACAATGAGAATTTGCACGACGCCATGTTTGGACGCCATCTTGGCATTTATTAGATTGTGTCAAACAGTGACAGTTGATTTGAGAGATTCGTTTACGCGTTCGATTTTTGAATTTATTTGAAGTTTGATGTGGTTTTGTTTGCGCAATATTTAATTAGCACCTAATTCTAGTTTGATTATGGTTAAAGGTTTAATATTATGACTAACTTTCTATTCTATATGTTATCTAATATGAACAATTTTAAAGGAACATGTGTTAAGAAGAGTAATAAGTCAAGTTTTTTATTTATTTATTTAAACTTTAT

>c22414_g1

CAAAAATACTGAAGAAAATCGTTGGACGAGTAGCCTCCCCAGCCACGGCAGCCCCGCTGCACCACGGGACTGGTTATCTACATTAAAGACTGGAGTAGTTCGTCAAACGATTTTCTTCAGTATTTTTGTAAAACCATCTCGAAGCTTTCCTCTCCCAATATTAAGTGAAGCCAACAACAGCGAAGATGTCTCTGAGTCTGGATCCCGGGCCCGGCATAGCTGCCCTGCAAGCATGGGGCGGCCAGGTAGCGGCGTATGGCGCCTCCAACCAGACCGTCGTGGACAAGGTGCCGCCTGACATGCTTCATATGGTCGATCCTCACTGGTACCAGTTTCCTCCGATGAACCCGCTTTGGCACGGACTCTTGGGATTCACAATCGGCTGTCTTGGATTCATCTCTATCACTGGCAACGGAATGGTCATCTATATTTTTATGTCAACGAAGAGTCTTAAAACTCCATCAAACTTGCTCGTGGTAAATCTCGCTTTCTCCGACTTCCTCATGATGTGCTGTATGTCTCCGGCTATGGTGGTGAATTGTTATAACGAAACATGGGTTTGGGGTCCTCTAGCATGTGAACTCTACGCTTGTGCTGGCTCGCTATTTGGATGTGCATCGATTTGGACCATGACAATGATAGCCTTCGACCGCTACAATGTAATCGTGAAAGGTATCGCCGCCAAGCCCATGACCAACAATGGGGCTCTTCTGCGCATACTCGGCATCTGGGTGTTCTCACTTGCATGGACTCTTGCGCCATTCTTCGGCTGGAATCGATATGTGCCCGAAGGAAACATGACTGCTTGTGGCACAGACTACTTGTCCAAAGATTGGTTCAGCCGCAGCTACATCCTGATCTACTCCGTCTTCGTATACTTCATGCCTCTCCTGCTTATCATCTATTCCTACTTCTTTATTGTCCAGGCCGTAGCAGCTCACGAGAAGGGAATGAGGGAACAAGCTAAGAAAATGAACGTAGCTTCCCTCAGGTCATCAGAAGCAGCTAACACCAGCGCTGAGTGCAAATTGGCAAAGGTAGCGTTAATGACCATTTCACTGTGGTTCATGGCGTGGACTCCGTACCTCGTGATCAACTACACTGGTGTTTTCGAAAGCGCACCCATCAGCCCTCTCGCTACTATCTGGGGCTCACTCTTTGCTAAGGCTAACGCTGTCTACAATCCTATTGTATACGGCATCAGCCACCCGAAGTACCGCGCTGCTCTGTACCAGAGGTTCCCGTCGCTGTCGTGCCAGGCGTCGCCCGACGAGAGCGGCTCCGTGGCGTCCGGCGCCACCGCCGTCTCCGAGGAGAAGCCCGCCGCCTGAGCGTCTCGCCTCTGCCTTACGACCGACACTTCCACGACTCGGCCTCGCTACTCTATGGACATACGATTCTGCCAAAGCGATGTTATTTATTATTTTTATACGCAAACATTTACATTTGTACAAAGCTATTTAAACTTGAACACAGAGCGTGTGCTGGTTGCAAAATATTCAGGTTGTCCACCTTACAAATCGGCACTTGCGTATTTTGAGTTCACGTTTTGGGTATCGAGTTGAACGTTGCACACTTTTCTTATACCTTACATTTGCTTTAAATATCGGTCGACGACATATAAAAAAATACAAAAAAAATACTAATCAGCAGTATGTCGCCAGGCCGCCCCTTAATCCATGAGACTGCTCCTCTTAGATATAGGCAATGCTATAGATATCTTTACGGTGAAACAGGCTCTGTATCTTGGACTAATAAAGTTAAGCAAGCAATGTGGTTAATGATCACAATAAAAAAAAAAAAAAAAAAA

>c31350_g2

TGAACTTGTGAAAGTAACACGCGCTCCTGTAATTTTTTCGAAAAATGGGATCAATATTTTTGCAATTTTAATTGAAGGATATTTTTTGTTTTTGGAAAGGATATATTCGAAAATAATTCAAGATGATGACCAAAGTGAAGGCCCAGGGCCTTGTGTCAGACTTGATGCCCAACATCAAGCTGATGCAGGCAGCCGGGCACTTCCTCTTCAACTATCATTCAGAAAATGCTGGCATGTCGAACCTTCTCCGCAAGATCTACGCGAGTACCCACGCCATCCTCATCATCGTTCACTTCGCGTGCATGGGCATCAATATGGCGCAGTACTCCGATGAGGTCAACGAGCTGACGGCCAACACCATCACCGTCCTGTTCTTCACCCATACCATCATCAAGCTTGGATTCTTTGCGCTGAACTCGAAGAGTTTCTACAGGACTCTAGCAGTATGGAACCAGTCAAACAGCCACCCTCTGTTCACGGAGTCCGATGCCCGCTACCATCAGATCGCGCTCACCAAGATGAGGAGACTCCTTTACTTCATCTGCGGAATGACCTGCCTGTCTGTTGTCACTTGGATTACTCTAACGTTCTTCGGTGAATCAGTCCGCATGATTACTAGCAAGGAAACCAACGAGACCTTGACGGAGGTAGTCCCTCGCCTGCCTCTGAAAGCCTGGTACCCGTTCAACGCTATGAGTGGCACCATGTACATTGTCGCCTTCGCTTTTCAGGTCTACTGGCTCCTCTTCTCAATGGCCATAGCCAACCTCATGGACGTAATGTTCTGTTCCTGGCTGATCTTCGCGTGTGAACAGCTGCAGCATCTGAAGGCTATCATGAAGCCTCTCATGGAGCTCAGTGCCTCCCTGGATACTTATAGGCCGAATACTGCTGAACTGTTTAGAGCTTCTTCAACTGAAAAGTCAGAAAAGATCCCCGACGCAGTTGACATGGACATTCGCGGCATATACTCCACGCAGCAGGACTTCGGGATGACCCTGCGAGGAGCTGGAGGTAGACTGCAGAACTTTGGGCAGCAGAACGCGAATCCCAACGGGCTGACTCCGAAACAAGAGATGCTGGCCAGGTCTGCTATCAAGTACTGGGTTGAGAGGCACAAGCACGTTGTGAGGTTAGTGGCTTCCATTGGCGACACGTACGGTACTGCTCTACTGTTCCACATGTTGGTGTCCACGATCACTCTCACGCTGCTGGCCTACCAAGCTACGAAGATAAACGGGATCAACGTGTATGCTTTCAGCACGATTGGCTATCTCAGCTACACTCTTGGACAAGTGTTCCACTTCTGCATTTTTGGTAATCGACTTATTGAAGAGAGCTCATCAGTAATGGAAGCCGCCTACTCTTGCCAGTGGTACGATGGCTCTGAAGAGGCGAAGACCTTCGTGCAGATCGTGTGCCAGCAGTGCCAGAAGGCTATGAGCATCTCCGGAGCCAAGTTCTTCACCGTCTCACTTGATTTGTTTGCTTCGGTGCTAGGTGCAGTAGTAACCTACTTCATGGTGTTGGTGCAACTGAAATAAAATGGATACAACAAGCATCGCTATAGTATTACTTACAAAATTTATGTAAATTATAAACAAAATACATCCTAGAATTTCATAACACCTAAGTTACAGCGTTAGCTAATATACAGTAAATTATTTCATTTATATAGGTATTCTTAAAAAATGCATAAAATGCACCACTTGAAAGTACAAAGTAATCAAATCAAAAATAGATGCATAGGCAAATTTACATCCCATTTATGGAGTCCAGGCAGGTTGGCGTGATGTCGCAATATTTTCAATCTTTGTAGATAAAAAAGTGATCATTTTTGATGTATAAGTATTAAAAGTATTTGATTATAGTTAGATTTACACTATGTAGGTTTTTTAAATCTCAATATATGCAAGTAGAAATACAAATATCAATATATTATTTATATTATAAACCTGACTAGGTACCTACATATATTTTATTTCTTTATAAGTGTATCAAAAATAACATTTCCGAATTCCAATATGGTCGCAATGACAGATTTTCTCCACTAGTATCAAAATTTACTATTACAGTTCTAGCGTGGAGCGAGACAAACATTTACTGAGTAAAAGCCACCTTTGCACTTCAGTTGCCTTTCAAAAATACCAGCGGCAGGACACATCAAAGACGATAACATCTCTTTATCTGTTTAACAATTGAATAGTTGTTACTTTCATCGCTAGTGCAGGTTAAATTTTCTAAGAATAAATTGTTATCTAAAGTGCTTTAGTTTTGGATTTCAGGCCTTCAACAGACTACCCGTAAGAAACCGGAATCCTTAGAATCTGTCACTAGTCTTGCCAAGTGGTGTCCTGTTCTTATAGTACTGGGTTATAGAGGTCAGATAAGTAGTCACTCCGTGTATAAATTACTGGAATTCAGGCGCATCACGATTCTGGATAGACTAGAAGTCGACCCCAACATAGTTGGATAATGGCTTGGTAGGTACATTTATTGTTATCGAAAGAAGTAAAAACAATGTCATTATATAACGAACATTAAAATATTTATTTAGTCATAAAAATGACATGATTATCGGAATATATTAATTGAAAAAAGAAACTACTTAATTTGTAAGTGCCTCATTAAAATTAAAGTAAATACGTAGCGAGCTTTTAAAAATTAGAAGTGATTATATAGATACAAATTATGAAGAAACTTATTTTTGTGATAGTTGATTTAGGTAATAAGATTTTATAAAAATAATCGTACCGGTAGTGTTTAATATTTTATCATCAGCACTTAATTTTAAAATGCAGTTACAAAATGTTTTATGATGTTTTAATGTGTAACTATTTTTATATTTTTATTTAAATTTTAAGTAA

>c25819_g1

AGATAGGTTATTGGGTTCGGCCATTAAACCGTAACAAAAACAAAATCCAATGCAATTTAAATTTAATTCTTTATTGAAATTAAACTTCGTGAATTTTAAACAAGCTAGATCCATGTAGGTATTAAATAAAAAACAAAGTGAATAGTGCAATATCAGAATTACCCGTTGATATATATTTTTTTATTCTTCTTCCTCTGCTACTGTCTGCAGCATCACGAAATATGAGAATGATGTTTTTAGAATCGCGATCATAGTCTGCACCCCCACAGTGAGCATGCTCATCGCCTTCAAGTTCATGGACACCTGGGACTGTATCAGCATGACCAGCACCATCTTGCTATCCTTGGTCTCCATGTACTCCCAAGGTACACAATATGCGGCGTCTATTAACTTGTCGTTCGATGATCCCAGCAGCTCGAATATAATGCTCAACTGGATCAGCTGTTGGAAGATCACCACCGTCAATGGTCCGTAACGCAACAGCGCCTTCTTGTCCATTTGGGAGCACTCTAACATCAGAAGGCACTCGCTCACTTGATGGAACAGGAAGTATATGAGAAGCACTGCTCCGAAACTGTCCGACATCCGCCTTTGGAAATCAATAATAACACTGTGGTAGTGAAGGTTCTCCCGCAATCTATTGTGAATATCTATCTGCTCCTGCTCATTATACTTGTGATCCTTTATCTCGCCATCCTTCGACTCGATCAGCAACGAAGCAGGTTTCGGAAAATTCTGCAGATTGTACTGTAAGATCCGCATGTGGCCCCAAAGGTGGAATACCAACAACGATATAGTTAGGTCTACAACACAGAAGTAGGATGAACAGGTACATGACATGTACCAGTTGAAAGTGAACAAAGCCAAGTAACCTCTCAAATTGGTAGTGTAGTCGAAAGGCAAGGAGTAGAACACGGAATGATCGAAGGTAGCATTTGGAGGCTTTACTTCTCGGTACATCCCATCTGTGTAGTTGTTGTACATCGGCGTCAGGTTGAACAACAATATACCGAGACACATCATTAGGAGGAGGTACATCGTAAAGAAATGAGATATTTTGTGCACTAGTATGTGGGTCTGGTAAGAGTAATCTGATTTATTTCTGTAATTAAAAAGGTGCATTTCATTCAGAAAATAGAAGAGAATGTCATTGTATTTTTTACTCAACACCATCAGTGCCCTCGAAAAAGCAATGGTATTCATAAAAACAGTAATGTACGTGTGACCCATCTCAAAAGACGACAGTTTCCCAGTATTGTTCCTTAGGTAGATGATGCCCGGGATGAGACAGCATATCTTTATAAAGACCATATAGTATCTATGGATCCGAGATTCATTTGGGGTCTTTGGCCAAGCGTCTAAGATCCACATACTCTTGCGCAGTATTTGCATATAAGGTATGTCTTCTGTTTTCTCCACCCCTTCTAGTTCACTAGCATCGGATACTAATTTCATGATGAAAGATTAAAATGCAAGATCTGCTTGTGTCCTTTCCTTCAGTAAGAATGTTTAGAAATAACGAAACTAAAGTATCTACTGCGCAAACCATGCCGCTATCCA

>c17613_g1

TTTTTTTCCACGCAAACGCATATTTATTTTTATGATTCATTTTTATATATTTTTGGTGCTCCATTTTTTATTTATTTGTGTTTCATTAAATTATAAATTAATACTAATAATAGTAAAGGTCTCGGTAAACGCACACAGGCTCCGTAAATGGGCAATCGGGAGTTCCGTGCAGTTTCCGAGAGCCGGGCAATGAGCTGGCATAATAAATAATACAATAAGGTACAAAAAGGAATTCAACGAAGTACCATCGGATGGTTTTCACAATCGCTCACACATACGCTCCACACACGCCAACAACAGTCTTACATCTATTATATCACTAGCTATACAGAATGCGGCGAGGCCGCGTGCAATGAACCATGGAAGTGTAGTACGGGCAGGCTGTTTACTCGAACTGCGAGTCCTTGAGCTTCTTGACGGCGTTGGTGAGGTGCAGCATCTCCGTGTTGAGCTGCCAGATGAGCGTCTCCAGCTTGTCCACGGAGTCCAGCACCTCCACGCGCTCCGTGTGCGGGTTGAAGCGCACCTCGAAGGGTCGCGACATGGTCGACACCCAGCGTCTGAATTTATCTTTGGCATCTTCGAAACTTTCAGCTACATAATAGATAGGTTGGTACTCCTGGTCCTGGTAGGGCTGCACCGAGGTGGACGCGGGCTCGAAGGGCCGGAGCTCGGGCTTGTCGCTGAGCGCGTGCAGCAGCTCGCCGATGGAGGACAGCAGCGCGGCGCCGTACGCCTTCAGTTGCTGGTTCTCCTTGCAGAGACCGAACTCCACGGTGAACCAGTACACGGTAGAGAGCTTTTCGATTTCAGCGTCCGAAGCGCCAAGAGATGCGAGACCGATCTCCTGCGAGAACTGGGCGAAGCTGAGGTCGGCCAAGAGGGGGATGTGTCCGAGGAGCTCGTGGATACAATCGGGTTCAGGAGTGTGGAAAGGCGAGTTGGCGTGACGAACGTATTGGGTAGATTGGAAGACTCGGAAGGCCAGAGATGCGAGGAAGTCGCGGGCGGTGAGCAGACCCGCGGCGGGGCGGAGCGTGAAGCCGGTGTGCTTGCGCAGGAAGTTGCTCACGTCCTCCAGCTGAGGGATGTGCTGCGGCACGAAGATGTTGGCGGCCTGCAGCTTGCCGAAGGCGACCTTGTACTCCTTGCAGGCGTGCTTGGGCATCAGGTCCAGCACGGTGTTGAACACTCGCTGCCAGGTCGCGTTCTCAGACTCCTTGTAGGTAATAGACGGGATCGGGTCTCCGTACTTGTAACCGAACGCGATCTCGGCAATTTGCTTCCTACGTTCTCTGTAATCCTTGTCGGCGAAACCGGGATGGTTCATATCCAATTCGGGTTCATATTTGGTCATCAGATGATTGCAGTTATCAAGATCCGAAGCGTGGCGAGGGAACCACGGGGTTTTGCTTGAGATGTTGTTCTCGGAAACGAGGTTAACTCCAGCGAAAGAGGTTGACTGACGAAGAGATCTGATAAGTTGCAATAGGTTGATGCGAGACATGCTCACTTTGACGAGAGCATCAAACTGGACACCGGTGAGTTGGGAGGGACGAGTTTCGAGATGCTGGACGCATCCCTTGTAGTTGTCGATGGTCTTGAGGATGCGCGCCAGAGAGCCCATTCCATCTCGCATGCGCAGCAAGAGCGCGGCTTGTTGGATCGCCTGCTCTGCCTCGGGAGATTCACTTGCAGCGTTCTGCAGGATCACTTCTTCCTCGGTCAGAGTATAGTCGTCGTCCGTTTTCCCAGCATCATCACCGATATCAGCATCAGCGAGATGACCGTTCTTAGTTTCATCTTGTTGGGTTCCATCTTCGACAGTCGGGGAGTTATCACCATTGCCAATATGAATGCCGTCTTGGATAAAATCGGAATCCAAGCCAGAGTCATTGGCGCGGGCGCGGGCTTCTTCTAAAACGCTCTGTTTGGTCTGTTTGACCACTAGTGTCTCGAACCTGGCATCGTCTACAAGCGAACGGCGACGAGACGGATAACCGTTCTCGATGCTGTAGGACTTCTTGATGGCGAACATCTCGCGGTTCTTCTGGGCAGCTGCGACGGCCATGGCTTCTTTCTGTTCTTCTCACGACACACCACTGGTATTGAAGGGCCTGAACGAGGGGTTCACTGCGCGGTTAGCTAGCGTATTGTCTGCGAGAGCTTTTTGTCTCGACCACGGGCTTCTGTGCCTGTCTGTCTGCGACTCGAGCTGTCGTATTACTATAGCTAACTAAACTTTGGATTGCAAACAGACCAATGCATGATGACCGTTCG

>c24989_g1

GCCATTTGTGTACAACGTTCCCAGCGCGCAACACGCGCCTTCTCCACCAGTGTAACAGTGCACTACTCGAACAGTTTCGGACAACATAAACTTGCAGCAATGAATTTCTCAATGCACCTGGCAGTAGTGGTGGCAGCGGCGGCTTGCCTCTGCGTGGTGGCGGCGGCGCCCGAGGGTAGACTGACCCGCACCAAGCAGCAGCGCCCCACGCGCGGCTTCAAGAACGTCGAGATGATGACCGCCCGCGGCTTCGGCAAGCGGGACAGGCCACACACCAGGGCCGAGCTGTACGGTTTGGACAACTTCTGGGAGATGCTGGAGTCTGCTCCTGAGAGGGAAGGACAGGAAACTAACGACGAGAAGACTTTGGAAAGCATTCCCCTGGACTGGTTTGTAAACGAGATGCTGAACAACCCAGACTTCGCGCGATCCGTGGTTCACAAGTTCATCGACCTCAATCAGGACGGCATGCTATCATCGGAGGAACTACTAAGGAATGTCGTTTAAACACATATTTAGTTAACTTATAACTGGAGAGACCTATTATTTGAAATCTGTTACTGCATGCAAAATATTTATATATTATAATATATTACGTATACTTGTATAGTCTATTTATTATTATTCAGTTACAGAACTTGTAACGTTACTGTGATAAATTATTACCTTCGCATTTCAAAAATTGAAAAAAAAAAACTATTTCATTTCTGTTAAAAGATAATTTTAAGACGGTACTGTTGTTTGGATTTTACGATTGTTGTAAATAATTTAAATTATAATGTCGATGGCATAAGAATTGTCTGTCCATAGAGATACTCTTTATATCTAAAAATGTAGATTAATATTCGTTATCGTAACTGAAAATTTCCTTTCTTGAGTGTGCCGCATTTAGAAATGAAACACGTAACAATTAAAATAGTTATTTACTTCCTGATATTATCTTGGTAAGCACATTGCGTGAGACAATCTGTCAGGTATTATGATAAAATAGTTTGACTTCAACCAAGCCATATAAGGAAGTAACAAATTATCTAAAACCACAAAACAGTTACCAATCAAGGTGTTGTAAGATCAGAGTAATTTAAGAGGTACAAGACTAAAAATGTATTTTAATCTTAGACATTATTTAAACTCTCCGACTACATGTTTCAATATCGTCTCGTTTTAAATGTGATTTTCGATTTATATAAGGGGAAAGGCGAATCTAACGATCTATTAATGTATGTTCTACGATAACTTAGGCTGGGTCATACTGTATCCTATTCATGTCAATTTCATAGATGAAATTATAGGAAGAAACTGTAGAGTCTGTCCTCATTTAGTCACTCTACATACATACATGTAATTTATATTGTAATGGATATAATAAAAATATTTTTAGGCGGTAAAATATTCGAAAACTGAATGTTTGGATACCCATCATTTGATTAGTTTGTTGTTAATTACCACTGTCGACGTTATGGTTGAAATAAAATAAATATATCGG

>c26255_g1

CGGGGCTCAATGGTGCTCCATCGATCGGCCACCATGTCGGCACGCCTGGCGCTGGTGGTGATCGCCAGTCTCTTCATCGCGGTGGAGTGTTCGCAAGAAATCATGAAGAATTTGGCCATAAACTTCGCGAAGCCTTTGGAAGACTGCAGGAAGGAGATGGACCTCCCAGACTCGGTGCTCACAGACTTCAACAACTTCTGGAAGGAGGGCTACGAGTTCACGAACAGACAAACCGGCTGCGCCATCCTCTGCCTCTCCTCCAAGCTGGAGCTGCTCGACCCGGAGATGAAGCTGCATCACGGGAGAGCCCAGGAGTTTGCGCAGAAACATGGCGCTGACGAAGCCATGGCGAAGCAGCTGGTGGACATGCTCCACAGCTGCATGCAGACTACGCCGGACGACGCCAACGACCCGTGCCTGAAGACCCTGAAGGTCGTCACCTGCTTCAAGACCAAGATCCACGAGCTCAAGTGGGCGCCCAGTATGGACCTCATCGTGGGAGAGGTCTTGGCTGAAGTTTAGAGGAACTCCTTACTTTTTTGTCTTCCTAGTACTGTCTTATATCTGTCTCTCTGTTTCTCCTTTATGCTGTGTTTTCTTTTTTGTTCGCGAAATGTATACATTATGCTTACTACTTGATGTTGCTTTTGCGTCTTTCAGAAAACCACTCTTATTTTGATTGTTAATTAAATAAATTTCTATCAGAATATACTCCAAAACTATCCTCAAAAATGTATAAACGTGCTGTCATTATCATCATCACTTCAGCCAAAGAAGTCCACTGCTGGACATGGCATAACGTGCTGTATTCTATGTAAGCTCCTTGTGTTTCTATCTCAACGACCAGGCGTGTTGAATGGGCCAGTTAGTGTTAAGTATCGTGTGAAGAATCTCCCGCCTCGCCAGCGTCTCCAGGGGGTGGGTACTACTGTAAGCTGCAACTACTTGATGTCATATTATCTATTAAAGAAATGTACATCTGAAAAAAAAAAAAAAAAAAAAA

>c17951_g1

TTTTTTTTTTCAAGTTATATAAGTTAATTTATTATGAATAAACAATGCACAGAAATAAATGCAATCCCTAGTTTGCAACCTAAAACATAGCAATAAGTTTTATCATAATTGGAATGAACCATATTCAATTCTCTTGAAGACTGGGAAGGTATTACCTGGGAGGACATAGTTTTAAACTTAAAAAATATTGTTTATACTTTTATAATTATTACAGAATTAAGTTAGGAGAGTTGACCACAGTCGGAGATGATGATTTTCTTTGAGGGCTTGCCAGACTGGGATCCGAAGGCTTCAACTTGCTTGACAACATCCATGCCTTCAACGACGGTACCAAACACAACGTGCCTGCCGTCCAACCAGGAGGTCTTCACGGTGGTGATGAAGAACTGGGATCCATTGGTGTTGGGCCCAGCATTGGCCATCGACAGGACACCAGGGCCGGTGTGCTTCAGGACGAAGTTCTCGTCGCCGAACTTTTCGCCGTAGATGGACTTGCCACCAGTGCCATTGTGGTTTGTGAAGTCACCTCCTTGCAACATGAAGTTGGGGATCACTCGGTGGAAGGTGGAGCCCTTGTAACCGAAGCCCTTCTCGCCGGTGCACAGGGCGCGGAAGTTTTCACAAGTCTTAGGGGTCACATCTGTTCTGAGCTCAACTACGATTCTTCCTAACGCTGATCCATCAGCGTTGACGTCGAAATAAACTCGTGGTAAAGCCATTTTGCTTTATTATGTAGAGTCTCGGACGAATTAGCTTTTATTCACCACAATGACGATGCGTCTCCTGTCGATGCCGGGCGGAAAC

>c24931_g1

GGAAATTGTCTATTATACACCGCCCGGTCCTGCCTTGTCCTTCAGCTGACATTTTAAAACTCCATCTCGTGGAGAGCATACGTGGAGTTACTTATTTACTTGTTTCTACATAAATTTATTCCGACATGTCCAAAATCGGTATCAACGGTTTCGGCCGCATTGGGCGTCTGGTCCTCCGCGCTGCCGTCGAGAAGGGCGCTACAGTCGTTGCCATCAACGACCCCTTCATCGGCCTTGACTACATGGTCTACCTCTTCAAGTACGACTCCACCCACGGTCGCTTCAAGGGCACCGTTGACATCCAAGATGGCTTCCTCGTCGTCAACGGCAACAAAATCTCCGTCTTCTCCGAGAGGGACCCCAAGGCTATCCCATGGGGCAAGGCTGGCGCTGAATACGTCGTCGAATCCACTGGTGTCTTCACAACCATTGAGAAGGCTTCAGCCCATTTAGAGGGTGGTGCCAAGAAGGTCATCATCTCTGCCCCCAGTGCTGATGCACCCATGTTCGTGTGCGGAGTCAACCTTGAGGCTTATGACCCATCTTACAAGGTCATCTCCAACGCTTCCTGCACAACCAACTGCCTCGCGCCCCTCGCTAAAGTAATCCACGACAACTTCGAGATTATTGAAGGTCTGATGACCACCGTGCACGCCACCACCGCTACCCAGAAGACCGTGGACGGACCTTCCGGAAAGCTGTGGCGTGATGGACGTGGTGCTCAGCAGAATATCATTCCCGCCTCCACCGGCGCTGCCAAGGCCGTCGGCAAAGTCATTCCTGCACTGAACGGAAAGCTGACCGGTATGGCTTTCCGTGTGCCCGTTGCCAACGTGTCCGTTGTGGACCTGACTGTCCGCCTGGGTAAGGCTGCCAGCTACGATGCTATCAAACAGAAGGTTAAGGAGGCAGCCAACGGTCCCCTGAAGGGTATCTTGGACTACACTGATGAGCAGGTTGTGTCATCCGATTTCATTGGTGACAACCACTCGTCCATCTTCGATGCTGCTGCCGGTATCTCTCTGAACGACAACTTCGTGAAGCTCATCAGCTGGTACGACAACGAGTTCGGCTACTCCAACCGCGTCATCGATCTCATCAAGTACATCCAGACCAAGGATTAAATGTTAGATTGCGTAGAGATAAATGTTGTTATGAATGTTTAAGAATATGATTTTGTCGACTAGATCGACTTTTATAATTTAAGCTTAAGTATAGCGGACAAATCTAAATTATTATTTAACCACTTTGATAGTGGTTTATTACACGTATAAATTATCACATTTATATATTTAATGCAGTATCGCAATTAAATCAGAACTCAATAAAAGTTACGAAATTAAAAAAAAAAAAAAAAAA

>c26533_g1

ACAATAGATTTTATATAATATGTATAAATAGTACTTATAATAATGTAGAGATTATAATATAGGTAAATATATAGAGACAGGTCGTAAGCAAAACAATATTTCAGACTCATATATTTAAATAAATTGTTCAGAAGAAGACAGTTCAAATGAAATGTATATTAATATATTATCCTAAAATATATTTGCCACGACTTAGGTAAACATTATACGCTATGACTAGACACAATCTACTTAGGTGGGTAATTAATTAAAAGTAGTAAATTAATGTCGTAAACACTGTCACCGAGTGAAGGGATAATCCATAAATACGTTTAGTAGTTTTTTTTTCTTAGCCCTGCCTACATTTTATTTAGTACTTTAGAAACGTCATTCAAAGTCGCTTTTAAACCATTTTGACAAATGTTTTATTTTAGATAAATAAGTATTGATTATTAGTACCTTAGCTGACAAAAAAGTGGTTACAATATGTATTTACATTGAATGTAGGTATACCCATATAATTAATTTTGGAATGGTCAATGACCTCATTATGAAACGCCCATTTTATGATAGCTAATTTGACAATGTTTGATGACGTTGGCGTTTCACAATGTGGCCATTGAGGTGTTAGGTTGCTACTTTATTGAAAAAATGTTGAAATGAGTTGAGTGCTACACAACTAGGTCATAGGGTCGATGCGTCGTCGAGTCCGTGGCGTTCACTTGCGGAAGCAGGAGATGGGGTTGAAGTAGCACTGGCGGAAGCGGACCTGGCGCTTGTCGGCGCGCCACTGCCGCGCCAGGCGCCCGTCCAGCGCGCGCAGGCCCCAGCCGCGCGGCTCCGCCTGCGGCCACGAGCGGCTCACTCTGCCCATCCTCTCCTCAGCATCGAGTTGTAGCAGCAGCTTGCGCAGGGCCGCCGTGTTGATGGTGTCCCAGGGGCCCGCCATCTCCATGTCTCCGTCCGGATGCGCCACCAGAGTGTTGTCAGCCTGCTCGTCCTCCGCCTCCATAGGTGCGGCACGAATTGTCACAAACAGCATAGCTAAGGTGGCGGCGACGATGGCCAAGTACACGTTGCACACGTTCGTTTTCATTGTGATCTTCAAACTTTAATCAACACGTAGAAATAATTTCAGCTGTTAACGGCTGGAAGGTTTTCGTGAAACTAGTAACGATCACTTTTTCAAGTCGGTATGAGAGGCTTCAGTCTGCTTCCGTGGTCGCCCCCGA

>c21896_g2

CAGGGCTCTGGGTCTTGTCGTTTGTGATCTGCTTTCCGCCGCTGGTCGGTTGGAAGGATAAGAAGCCTGAAGAAGCAGACATGAAAGATGGCTGGGTCCCCAACCCGCCCTGCGAATGGACCTGTGAGCTGACGAACGATGCTGGTTACGTCGTCTACTCAGCTTTAGGCTCCTTCTACATCCCCATGTTCGTCATGCTGTTCTTCTACTGGAGGATATACAAAGCTGCTGTGAGGACTACTAAAGCGATCAATCAGGGTTTTAGGACTACGAAAGGTAAAGGGGGCATGGGTAGTCGCTTTGACGACAACCGCCTGACCCTCAGGATACACCGGGGCCGAGGGTCCAACCGTCCCCACGGGTCTCCACTGTCTAATGCTTCCAACCACTCCACCAGCACTTCACTGAGTGCTTCTCCAGAACGATTAAGAAGACACTCAAGCGCAAGACGAGCACACGAAAAAGTAAAGATCTCCGTGTCCTACCCCTCATCAGAACAGATCTGTCCTGCCCACGAGAACTCCAGGTCTTCTAGCAGATCCCCCAGCCCTTCCCTGTACGCAGTCCACTACGAGAGAGATGGGAGAGAGTTGACTGAAAGCAGGTTGAGGGTGCGACCCTCACATCATCTGGCTCCAGGCCCGTTGTATGATGAATACGATGATAAGCCGAGAACTACTCGGCGGATGGGGAAGAGGAATATTAAGGCGCAGGTGAAACGCTTCCGCATGGAGACGAAAGCAGCGAAGACCCTCGGCATCATAGTAGGAGGCTTCGTGTTCTGTTGGCTGCCCTTCTTCAGCGTGTACGTCGTGCGGGCCTTCTGCGGAGACTGCGTCAGCCCCATCGTGTTCTCTGTACTCTTCTGGCTGGGGTACTGCAACTCGGCGATAAATCCGCTGATTTATGCGCTGTTCTCTAAGGACTTTAGATTCGCATTCAAGCGCATAATCTGCAAGTGTTTCTGCGGCGGCGGAGGGCCGAGACGAGAGTCGGACGGCGAGGGTTCCCGGCGCCAGAACAACCGGCCCACTCACTCCCACTCCTTGGAGGAACATGACGCGAACAACCACACTACTTCTACCAGTACTACTTCTGCGGCTGACAGGTGACGACAAGCAATGCGACGGCAGAACCCACAGTTCCAGTCGACCGTCGCATGCGTTTAGCGAATAAAGTTATAATAACCATTGAAAACAGTACAACAATAATACAGTCTATAAAACAATTTTTTATCTGTATATTGTTATTAAACTCGTCCTACTGTAGAAAGTCAACCTAACTGTTTAATATAGGTAATGTGTGTATAAAATATGTCGTTGTAACATAAAAATAACGTAGTTATTTACCGTTGTAACGGATGAATAGCTTAATCACGAGCTATTTTAAAACTATAAATGTGCGTATTTCAGTGAATGTTTTTTAAAAGTGAATTTATTTATTGAAAAGACTGTCAGAACTTAATTGTACCTAATATTACGTAATAAATGATGTCAGATAAACAATATGTTGTTATAAAGAGGAACTATAAAGTGTTAAGATAGAGAAATAAGTAGTCATAATTAGACGATGTGGTGTGATATTCACTGGATATTTCGATGTTCATGTTTGTACACGCTCTAAGTCTGCTCTAGTAACTCCAAATTTAAAACGCTTATAACTGATACAATGTTGTACTTATCCTAGTTACTTTATCCTCACTCAACTCTAAAGAGTACATTAGCTGTGTATTATGCCTAATGACTATGAATAATGATGTACCTTTTCCATAAGTAGATAAGCTAGAGGCCCTTTAAGGCTGAGATTTTGGACATTGTCGTTGTTTCTCTCTGTTATTTATTTCCCGTTAATGTCCTTTTCACCAATGTCATAGATCGGTT

>c20809_g1

GATTAATTTACGCATTTTTATATTTAATATGCTTTGTTCAAACAAATAAACTGATTATTAGTTTCAATATTAATGTATATCTACGATTATGACATCTTATCTGGCAACACTGACACCGAGGCAGTGAAGCGAGCCGCACAGCGAGCGTGTGCGAAGAATAGTTTTAGTCCGATTCCCGAACTCTGTTGGTGTATGTGTGACGCATTCTTTACGTACAGCTACTATAAGGCAGTCGAGCGAGCGATCGATCGTGAACGTCGTCCGTTTCGTTAGTCGCGGGTTTCGCACTCATGTTGGAGAGTTGTGTTGTTTGTTGTCAATCCGTTTTACGTTTGACAAGTGAGAGTGAGAACTGCTTGTAGTACCTAGTCAAAAACAATCAATTTGATGTGGTCGTGTTCATTGCCTTGTGTAAAAACAGCACTATCTGGATTACAAATGGAAAAAGGAAATGGACCAATCCAAATCCATCCCACCTCTGCATTGCCGAGGTTCAAGCTCGCGGCTCCGCGCGACGCAGCTAAGCTAAAACCCCTGGCCAGTCTTGTGCTACCGACGTCCAACTACGACTCAAATGCCGGCTCTCCGGCTTCGTCGCCTTCATCATCGTCTTCTTCCTCTACGTTCTCCGTCGACAAGACCGACAACCACGACTTTAGCTTTGGCACGGAATCAGTTGATATGCGCACAGAAGATGCACGAATTAAAACGTTCGAAAAATGGCCTGTCACTTTCCTTTCTGGAGATGAACTCGCGCGAAATGGATTTTATTACCTCGGCCGTGGTGACGAAGTACGCTGCGCATTCTGCAAGGTTGAAATTATGAAATGGGTTGAAGGCGACGATCCTGCTAAGGACCATCAGCGATGGGCACCCCAATGCCCATTCGTTCGAAAATTGGGCAGCAGCGCCAGTTCAGAGACGAGTAGCAACGGGCGCGACGAGTGCGGTGCCCGCGCTGCCACCAGCAGCACTACGCCCCCAAGAATGACCGGCCCCGTGCACCCTCGCTACGCCTCCGAAACCGCAAGACTGCGCAGTTTCCAAGACTGGCCGAGATGCATGAAACAGAAACCTGAAGAGCTCGCAGAGGCTGGCTTCTTCTACACCGGTCAAGGTGACAAGACTAAATGCTTTTACTGCGACGGAGGCTTGAAAGACTGGGAGAACGATGACGTGCCCTGGGAGCAGCACGCGCGTTGGTTCGACCGCTGCGCATACGTGCAGCTGGTGAAGGGACGCGAGTACGTCCAGAAGGTGATGACGGAGGCGTGCGCAGTGCCCGCAGCTGAAGCCGGGCGCGACGTAGCGCCCGTGCGGACCAGCACCTCCAGCTCGCCGCTCGACTCGCCCGAGAACTCCGTGGACGACTCCAAGTTGTGTAAAATTTGTTACGCGGAGGAGCGCAACGTGTGCTTCGTACCGTGCGGACACGTGGTGGCGTGTGCCAAGTGCGCGCTGGCGGCTGACAAATGCCCAATGTGCCGCAGGACGTTTCAGAACGCAGTGCGATTATATTTCTCTTGAGAAGAGCTGCCTAATCGAAGACTCGATTCTAGTCAGGGACGGCCCGACAGAGCTGGTGTTTGAACCCTCTAGCTCCTATGTCACGTTTCCACAGGCGGAGAACCCTGTTGACATTTATCGTCTGGTAGATACGATAGGAACTCGCTGGTGTCAAGATAGACGAATACGCTTTTACAGTCCATTGAGATTGAGTCCTCAAGTGGGTTTTAGTCAGTACGAGTCTGACACGCCCTCTCGCTCCATCCTAAAGCGGGAGAAGTCATTTGATGATTTCCCACTTAAAACAAAAAAAAAAAAACGAGTACGAGA

>c31937_g1

GGGCGTAGTCCGAGCCAGGCAGTGATTTAGGCACACCGCGTATCCGTGACCACACAGTACCGCGTAAATTCACGGACATTGTCCGAAAAAAAATTAGTAATAATTAAGTAGGTGACGCTGTGTTTTTGTTGAACGTCTACTGTTTTTGTGTTTCAAACCTTTTATCCTGATACATTTGCAACCACCATGTCGGATCAGGATTTAAAACTCAAATTCGAAACGATGGAGGCCGGAGATTTTAAAGATTTCGCGAAGGCGATGACGGACTACATTGCTGAATATTTGGAAAATATCCGAGACAGGCAAGTAGTCCCATCGGTAAAACCAGGGTACTTGCGTCCGCTGGTGCCAGAACAAGCTCCCGAGAAGGCGGAGCCCTGGACGGCGGTGATGGCGGACATCGAGCGCGTGGTCATGTCCGGGGTGACGCACTGGCACTCGCCGCGCTTCCACGCGTACTTCCCCACCGCCAACTCCTACCCCGCTATAGTGGCTGACATGCTCAGCGGGGCCATCGCCTGCATTGGATTTACCTGGATTGCAAGCCCTGCGTGCACAGAGTTAGAGGTGGTGATGCTGGACTGGTTGGGCCAGATGTTGGGCCTGCCCGAGTCGTTCCTGGCCCGTTCAGGAGGAGAGGCCGGCGGCGTGATCCAAGGCACCGCCAGCGAGGCCACGCTGGTCGCGCTGCTCGGAGCCAAGAACCGCACCATGTTACGCGTTAAGGAACAACATCCTGAATGGACTGACACCGATATTCTGTCGAAACTCGTCGGTTACTGCAACAAGCAAGCCCATTCTTCTGTGGAACGAGCTGGTCTCTTGGGCGGTGTGAAACTTCGCGCTTTGCAGCCAGATGGAAAGCGAAGACTTCGTGGGGACACTCTTAAGGATGCCATTGAAGAAGACGTTCGCAACGGCCTTATCCCCTTCTATGTCGTGGCTACTTTAGGAACCACATCCTCATGCGCGTTCGACAATTTAGAAGAAATTGGCGAAGTGTGCTCTTCGAAGAACATTTGGCTGCACGTTGACGCAGCCTACGCCGGCTCCGCATTCATCTGTCCTGAATATAGATACCTCATGAAGGGCGTCGATAAAGCCGATTCCTTTAACTTTAATCCGCACAAATGGATGCTGGTTAACTTCGATTGTTCCGCCATGTGGCTCAAGGAACCGCGGTGGATCATCGATGCTTTCAACGTTGACCCATTATATTTGAAACACGATCAGCAGGGATCAGCCCCTGACTACCGTCACTGGCAAATCCCATTAGGCCGTCGTTTCAGAGCGCTCAAGTTGTGGTTTGTCTTACGTCTGTACGGCGTTGAAAATCTCCAGAAACACATCAGAAAACACATTGCATTGGCTCATCTATTTGAGAGACTTTGCAGCGCTGACGAACGGTTCGAAATTTACGAAGAAGTTACAATGGGACTTGTCTGCTTCAGACTGAAGGGCGGTAACGAACAGAATGAAGAATTACTCAGGCGTATAAATGGTAGAGGAAAAATTCATTTAGTCCCATCTAAAATTGATGACACTTATTTCCTCAGAGTAGCCATCTGCTCACGCTTCAGCGAAGAAAGTGACATTCACATATCTTGGGAGGAAGTAAAGGCTTCTGCTGACGAAATACTTAAAGCCCGTTAAAACTATTATGCATTTAAATATTCAGCAATAATTTAGAAGTTATTATAAGCGATTGCTACCAACTTCAAATAAATAAAATTATTCGATTTCAGCTATAAGCATAAAATTGTACCTATGTGTGTATGCTCGTCCTCACATTCAAGTGAGACGGGAAGGGTTGTACTTACATACGCGTTTTTAAATGTATCGTATGGACAGACATTAAGATGCTGACAAACCTGACCATTCTATGTAACAGAACGACGATCATTCGTCGTCTCTACACCGACGATAACGCTCCTGTTTTGTATTAAGAATGATCGTAATTACGAACGGCAATAAAATGAATGTTCAGGTTTTGTCATCTGTTTAAATATAAACGTCTGCATTAATTGGACAGCTCTCACTCAAGTAATGGGTTACTAATTAATGCAGCCGTCTCTCTAGCTACATGATAAATACAGTCAGGGAAATAAATTGTTTATCACCTTATAGTAGTTGGACACTTAGCACCAATTATGCGAAGTGCGACAATCTCATCCTTCTAAAAGTTTGGATTTTCCTAAATATTTTATTAAAAGTGTAAACAATTAATTTCCAGATCTGTATCGATAAATATTTGGAAGTAATTGAAAAATGTATCTATGATATAAACGTAGACTTTGTTGTATTAGAGGCACACTATAACTATACCATAGCTGTGATAGAATGTGTTTTTTTAATGAATTGCCGTTAAAGACAATAAAAGTAGAAAATTAATATCTATTAATATTAGGTATAATCTATAATATTATGACGTACGTGTCATTGAATACTACTTGCAGGTACCTCTAACTTCAGGGGATTACTGCATAGCCAAACTTGCCGTTAATGTTTTTTTAAGTAGATATCATTACTAGGACTACTAACATATCATATGCTAGGTGCATATGATATGTTCTTTACATCGTATCTTTTTTTTTAAATAATTAATATTTAAAAGAATATTAGAACACATTTCCTAAAGGTAAGAAAATGAAAATTGTTTACTAAAAAAACCAGAGAAGTAAAGTACGTATCGAATAATCTCATGTACTTATATTTTAGTGAATTTCAAGTGTTAACAGATTTTAGCTGCTGACCGTACCTACGTCGCCTATTGAGTCTTGACTTGTGATTGTGTTAATTTATTATTGGATTAGACCAACGCGCTTAATATTGCCTACCCCTATTTTTTAAATAATTTAGGATTTACGTACAAATTGTGTCTTGTTCATAAGAAACAATATTGGTTGATCTTTGAACTAAAATCTTCTGCAAGCGTATAAAAACTCTTTGGTAAGATCTATTACCAGATTTTATTTGTTGTTTCTTTCTTTCTTTCAAAAAAGTTCTGAATCACCAAATTACAGGTACTTTGAACCTTAACTACTTTTGCTCAGCCAATGACTCTTCGTCGTTAACCCGCATTCCTAATAATTCTAGACATATTTACCTACTATTATATTTTAATTTATGTATATTATAGGTACCTACCCTAATTTAAATTGTCGATGTGTAATGTTTACCTATCTTAAATCTATGTGTCTATTTAAAATATAGCTGTTTGTTATTTATTATATTTAAAGCGTAATAAAGAATATCATAAAACAATAAAAA

>c28260_g1

AGCAACATGTCCTGGCTGTGTCCCGAGACTTCAGTTCCCAGCCCCGTCTGACCTACAAGACGGTGTCGGGTGTGAACGGACCCCTGGTCATCTTGGACGAGGTGAAGTTCCCCAAGTTCTCTGAGATCGTGCAGCTGAGACTTGCTGATGGCACCCTCCGTTCCGGTCAGGTGCTGGAGGTCAGCGGCTCCAAGGCCGTGGTCCAGGTGTTCGAGGGTACCTCGGGTATCGACGCCAAGAACACTCTCTGCGAGTTTACCGGCGATATCTTGCGTACCCCGGTGTCTGAAGACATGTTGGGTCGTGTATTCAACGGATCCGGCAAGCCCATCGACAAGGGACCCCCCATCCTGGCCGAGGCTTTCCTGGACATCCAGGGCCAGCCCATCAACCCCTGGTCCCGTATCTACCCTGAGGAGATGATCCAGACTGGTATCTCTGCCATTGACGTGATGAACTCCATCGCTCGTGGTCAGAAGATCCCCATCTTCTCCGCGGCCGGTCTGCCCCACAACGAAATTGCCGCTCAGATCTGTAGACAAGCTGGTCTTGTCAAGATGTCCGGCAAATCCGTGATCGACGACCACGAGGACAACTTCGCCATCGTATTCGCCGCTATGGGTGTGAACATGGAAACCGCCCGGTTCTTCAAGCAGGACTTCGAGGAGAACGGCTCTATGGAGAACGTGTGCCTGTTCTTGAACCTGGCCAACGATCCTACCATTGAGAGAATTATCACTCCACGTCTGGCTCTTACTGCTGCTGAGTTCTTGGCTTATCAGTGTGAGAAACACGTGTTGGTCATCTTGACTGACATGTCCTCATACGCCGAGGCTCTGCGTGAGGTGTCGGCCGCCCGTGAGGAGGTACCCGGACGACGTGGTTTCCCAGGTTACATGTACACCGATTTGGCCACGATCTACGAGCGCGCCGGACGTGTAGAAGGCAGGAACGGCTCCATCACCCAGATCCCCATTCTTACTATGCCCAACGACGACATCACCCATCCTATTCCTGACTTGACGGGTTATATTACTGAGGGACAGATCTACGTAGACCGTCAGCTGCACAACAGACAGATCTACCCGCCAGTGAACGTGCTGCCGTCGCTGTCGCGTCTCATGAAGTCTGCCATCGGCGAGGGCATGACCCGCAAGGACCACTCCGACGTGTCTAACCAGCTGTACGCTTGCTACGCCATCGGTAAGGACGTGCAGGCGATGAAGGCCGTCGTCGGAGAGGAGGCGCTGACGCCCGACGATCTGCTCTACCTCGAGTTCCTCACCAAGTTCGAGAGGAACTTCATCTCCCAGGGTAACTACGAGAACCGCACAGTGTTCGAGTCCCTGGACATCGGCTGGCAGCTGCTGCGTATCTTCCCCAAGGAGATGCTCAAGCGTATCCCCGCCTCCATCCTCGCCGAGTTCTACCCCAGAGACTCGCGCCACTAAGTGGACCGCCAGAGGTCGCACGCGCTACTGCACGGACAGCAACAGAGGGCAATCCGGGACAATACACGACAACTCTAATCTGACTATCCTTGCCAGGACTATGAAGCAATACATCGTAATGCCACTAATTGTTCTAAACATTAATAAATTAGATAATTCTATAACACAATTAGAGGCAATTAAGTCCACAATTGCTTCAATACCTCTGTCAAATATAGTCAGATAAAAATTGCCAGAAAAATTGCTCATTCAAGCGCTACACAAGATCTTCCTGCGAGAAATATCCAACGAATGCGACCAGTCAGCACTGCGACCATGATATTCATGTAAGATAAAAATACTGTTTAATAGTAAGTTGTTAATAGTTTATTATATAATGTATTACATACATGTATTATGATCATGTGCAAAATTATATAAACGCGTTTAAATCATAAGTAAACAGCCCGTAATGCTGAACAGACTTGTGTATATATTTTCATCGTTAATATCATAGAATGTACTGAGCAGTTAAATTATTATATTTATAAATAGATGCCATTTTTCTTTATTTTTTTGGTGCTTCGAACACAGTATAAACGATATATAAAGCTAAAGGCAAAAAAAAAAAAACGTTATTTTGTGTGCACAAATTGTTGACATGTGTTCAAATTATAAATGTAATAATAATCGAAATCTGTTGTAAATCAAGATACTATTTAATGTGTGATATCGCTTGGAACTTTGCCGTTTGAGATGTGACATATAATATGTTGATGTAAATAAACCATAGCCCAGTAACGACACGTTACATTCCAAATTATGTTTTCCTGTTCACTCCAGCTAGATTCTGTATTCAGCTAATTAAAGTGAATTAGGATACTGTTGTTTTCAATGGAAATAAAATATA

**Supplementary Table 3.**

**Table 3a. Candidate migration genes identified in *M. separate*.**

| Gene | Unigene ID | ORF (aa) | Best blastx match | | | Identities | Complete | RPKM/  FPKM |
| --- | --- | --- | --- | --- | --- | --- | --- | --- |
|  |  |  | Nr Description | Species | ID |  |  |  |
| *per* | c31643_g2 | 1192 | Period | Helicoverpa armigera | AKC42321 | 77% | Yes | 31.38 |
| *clk* | c30631_g2 | 346 | CLOCK | Helicoverpa armigera | AKC42319 | 89% |  | 7.35 |
| *cyc* | c31074_g1 | 697 | Cycle | Papilio xuthus | XP_013162322 | 65% | Yes | 12.96 |
| *vri* | c29769_g1 | 366 | vrille | Danaus plexippus | AAT86041 | 85% | Yes | 60.2 |
| *tim* | c32116_g1 | 1043 | timeless | Spodoptera exigua | AEJ38225 | 84% | Yes | 36.24 |
| *cry1* | c31435_g1/g2 | 528 | cryptochrome 1 | Mythimna separata | AFR54426 | 100% | Yes | 12.21 |
| *cry2* | c31387_g4 | 591 | cryptochrome 2 | Mythimna separata | AFR54427 | 99% |  | 13.6 |
| *sgg* | c28955_g1 | 435 | shaggy | Danaus plexippus | ABU49716 | 98% | Yes | 94.69 |
| *pdp1* | c21770_g1 | 263 | PAR-domain protein 1 | Ostrinia furnacalis | AGR44476 | 91% | Yes | 30.37 |
| *ck2a* | c17691_g1 | 351 | casein kinase II alpha | Spodoptera frugiperda | O76484 | 100% | Yes | 35.77 |
| *ck2b* | c25884_g1 | 225 | Casein kinase II beta | Spodoptera frugiperda | O76485 | 94% | Yes | 90.11 |
| *met* | c40384_g1 | 126 | methoprene-tolerant homolog-2 | Bombyx mori | BAJ05086 | 71% |  | 1.04 |
| *slmb* | c39876_g1 | 186 | Slimb | Danaus plexippus | ABV22506 | 96% |  | 1.6 |
| *dbt* | c31342_g1 | 346 | double-time | Spodoptera exigua | AEJ38223 | 99% | Yes | 9.7 |

**Table 3b. Candidate melanin synthesis related genes identified in *M. separate*.**

| Gene | Unigene ID | ORF  (aa) | Signal  Peptide | Best blastx match | | | Identities | Complete | RPKM/  FPKM |
| --- | --- | --- | --- | --- | --- | --- | --- | --- | --- |
|  |  |  |  | Nr Description | Species | ID |  |  |  |
| *TH* | c17613_g1 | 561 |  | tyrosine hydroxylase | Mythimna separata | BAF32573 | 99% | Yes |  |
| *DDC1* | c31937_g1 | 488 |  | dopa decarboxylase | Mythimna separata | BAB68549 | 100% | Yes |  |
| *DDC2* | c26899_g1 | 500 |  | Dopa decarboxylase 2 | Operophtera brumata | KOB58186 | 75% | Yes |  |
| *ebony* | c31839_g1 | 862 |  | Ebony | Chilo suppressalis | AKL78853 | 78% | Yes |  |
| *tan* | c30840_g3 | 392 |  | tan | Bombyx mori | NP_001170882 | 77% | Yes |  |
| *aaNAT1* | c31939_g1 | 259 |  | arylalkylamine N-acetyltransferase | Antheraea pernyi | ABD17803.1 | 64% | Yes |  |
| *aaNAT2* | c29401_g2 | 223 |  | dopamine N-acetyltransferase-like | Bombyx mori | NP_001296534 | 56% | Yes |  |
| *GTPCH* | c27740_g1 | 291 |  | GTP cyclohydrolase I isoform B | Bombyx mori | NP_001138797 | 96% | Yes |  |
| *PPO1* | c10055_g1/ c39766_g1 | 200 |  | prophenoloxidase 1 | Mythimna separata | BAM76811 | 100% |  |  |
| *PPO2* | c30210_g2 | 693 |  | prophenoloxidase 2 | Mythimna separata | BAM76812 | 99% | Yes |  |
| *LAC1* | c31746_g1 | 623 |  | laccase 2 | Papilio xuthus | KPI91648 | 76% | Yes |  |
| *LAC2* | c32155_g1 | 811 |  | laccase 4 | Papilio xuthus | KPI97763 | 71% | Yes |  |
| *yellow-c* | c30567_g2 | 426 | - | yellow-c | Bombyx mori | AFC87785 | 74% |  | 61.59 |
| *yellow-f1* | c31466_g1/g2 | 359 | 1-20 | yellow-fa | Bombyx mori | AFC87787 | 63% | Yes | 56.4 |
| *yellow-f2* | c29411_g1 | 424 | 1-18 | yellow-f4 | Heliconius melpomene | ADX87356 | 51% | Yes | 13.22 |
| *yellow-d* | c30885_g1 | 449 | 1-24 | yellow-d | Bombyx mori | AFC87786 | 74% | Yes | 53.84 |
| *yellow-h* | c20824_g2/g1 | 424 | No | yellow-h3 | Heliconius numata | ADD60453 | 83% | Yes | 4.99 |
| *yellow-x* | c28958_g1 | 446 | 1-14 | yellow-x | Operophtera brumata | KOB66859 | 81% | Yes | 232.29 |
| *yellow-b* | c31930_g1 | 458 | 1-18 | yellow-f | Bombyx mori | ALL54586 | 81% | Yes | 34.71 |
| *yellow* | c37756_g1 | 174 |  | yellow | Heliconius melpomene | ADX87342 | 82% |  | 2.66 |

**Table 3c. Candidate olfactory genes identified in *M. separate*.**

| Gene | Unigene ID | ORF  (aa) | Signal  Peptide | Best blastx match |  |  | Identities | Complete | Conserved domains | Group | RPKM/  FPKM |
| --- | --- | --- | --- | --- | --- | --- | --- | --- | --- | --- | --- |
|  |  |  |  | Name | Acc. No. | Species |  |  |  |  |  |
| MsSNMP1 | c31246_g1 | 525 |  | sensory neuron membrane protein 1 | AGN48098 | Spodoptera litura | 89 | Yes | CD36 |  | 45.38 |
| MsSNMP2 | c27191_g1 | 520 |  | sensory neuron membrane protein 2 | AGN48099 | Spodoptera litura | 88 | Yes | CD36 |  | 175.63 |
|  |  |  |  |  |  |  |  | Yes |  |  |  |
| MsCSP1 | c28555_g2 | 128 | 1-18 | chemosensory protein 3 | AGR39573 | Agrotis ipsilon | 85 | Yes | PBP |  | 9145.83 |
| MsCSP2 | c25454_g1/g2 | 107 | 1-18 | chemosensory protein 5 | AGR39575 | Agrotis ipsilon | 88 | Yes | PBP |  | 11 |
| MsCSP3 | c28555_g3 | 127 | 1-18 | chemosensory protein 1 | AND82443 | Athetis dissimilis | 82 | Yes | PBP |  | 425.83 |
| MsCSP4 | c25813_g1 | 128 | 1-16 | chemosensory protein | AAF71289 | Mamestra brassicae | 81 | Yes | PBP |  | 14745.7 |
| MsCSP5 | c26967_g1 | 122 | 1-17 | chemosensory protein 6 | AEX07267 | Helicoverpa armigera | 84 | Yes | PBP |  | 491.06 |
| MsCSP6 | c30240_g1 | 122 | 1-23 | chemosensory protein 14 | AKT26490 | Spodoptera exigua | 86 |  | PBP |  | 24.86 |
| MsCSP7 | c17971_g1 | 125 | 1-16 | chemosensory protein | AGY49266 | Sesamia inferens | 60 | Yes | PBP |  | 3517.73 |
| MsCSP8 | c25444_g1 | 122 | 1-16 | chemosensory protein 10 | AND82452 | Athetis dissimilis | 98 | Yes | PBP |  | 91.66 |
| MsCSP9 | c27600_g1 | 127 | 1-18 | chemosensory protein 6 | AGR39576 | Agrotis ipsilon | 99 | Yes | PBP |  | 3241.2 |
| MsCSP10 | c27994_g1 | 124 | 1-16 | chemosensory protein 2 | AND82444 | Athetis dissimilis | 86 | Yes | PBP |  | 20.9 |
| MsCSP11 | c3142_g1 | 86 | ? | chemosensory protein | AOG12895 | Eogystia hippophaecolus | 59 | Yes | PBP |  | 0.71 |
| MsCSP12 | c3874_g1 | 95 | 1-19 | chemosensory protein 3 | AND82445 | Athetis dissimilis | 94 |  | PBP |  | 3.09 |
| MsCSP13 | c21517_g1 | 125 | 1-15 | chemosensory protein 12 | AKT26488 | Spodoptera exigua | 70 | Yes | PBP |  | 18.98 |
| MsCSP14 | c45234_g1 | 106 | 1-16 | chemosensory protein 5 | AND82447 | Athetis dissimilis | 91 | Yes | PBP |  | 7.68 |
| MsCSP15 | c25484_g1 | 111 | 1-18 | sensory appendage protein-like | AAK14793 | Mamestra brassicae | 73 | Yes | PBP |  | 5141.73 |
| MsCSP16 | c25284_g1 | 120 | 1-16 | chemosensory protein 2 | AEX07265 | Helicoverpa armigera | 88 | Yes | PBP |  | 23442.37 |
| MsCSP17 | c24984_g1 | 127 | 1-18 | chemosensory protein 8 | AND82450 | Athetis dissimilis | 81 | Yes | PBP |  | 676.56 |
| MsCSP18 | c27178_g2 | 149 | 1-22 | chemosensory protein | AGY49270 | Sesamia inferens | 92 | Yes | PBP |  | 1045.38 |
|  |  |  |  |  |  |  |  |  |  |  |  |
| MsGOBP1 | c27221_g2/g1 | 162 | 1-18 | general odorant binding protein 1 | AGS36742 | Sesamia inferens | 88 | Yes | PBP_GOBP | PBP-GOBP | 1558.68 |
| MsGOBP2 | c22940_g1 | 162 | 1-21 | general odorant binding protein 2 | AIS72932 | Spodoptera litura | 93 | Yes | PBP_GOBP | PBP-GOBP | 10963.07 |
| MsPBP1 | c26255_g1 | 170 | 1-27 | pheromone binding protein | BAG71416 | Mythimna separata | 98 | Yes | PBP_GOBP | PBP-GOBP | 20192.14 |
| MsPBP2 | c23411_g1 | 149 | 1-23 | pheromone binding protein 1 | AAC05702 | Mamestra brassicae | 85 |  | PBP_GOBP | PBP-GOBP | 3332.51 |
| MsPBP3 | c18728_g1 | 140 | 1-20 | pheromone binding protein 3 | AFM36758 | Agrotis ipsilon | 86 |  | PBP_GOBP | PBP-GOBP | 3742.95 |
| MsOBP1 | c23590_g1 | 149 | 1-21 | odorant binding protein 5 | AEB54581 | Helicoverpa armigera | 75 | Yes | PBP_GOBP | ABP-I | 379.22 |
| MsOBP2 | c23590_g2 | 146 | 1-21 | pheromone binding protein 4 | AAL66739 | Mamestra brassicae | 84 | Yes | PBP_GOBP | ABP-I | 1897.94 |
| MsOBP3 | c20537_g1 | 149 | 1-22 | odorant binding protein 26 | AKT26503 | Spodoptera exigua | 78 | Yes | PBP_GOBP | ABP-I | 4.38 |
| MsOBP4 | c10494_g1 | 148 | 1-22 | odorant binding protein | AAR28762 | Spodoptera frugiperda | 55 | Yes | PBP_GOBP | ABP-I | 5.92 |
| MsOBP5 | c20205_g1 | 139 | 1-21 | odorant binding protein 13 | AGP03459 | Spodoptera exigua | 39 | Yes | PBP_GOBP | ABP-I | 126.9 |
| MsOBP6 | c25084_g1 | 146 | 1-20 | odorant binding protein 3 | AEB54582 | Helicoverpa armigera | 38 | Yes | PBP_GOBP | ABP-I | 27.17 |
| MsOBP7 | c26404_g1 | 145 | 1-21 | pheromone binding protein 4 | AAL66739 | Mamestra brassicae | 34 | Yes | PBP_GOBP | ABP-I | 72.45 |
| MsOBP8 | c21768_g1 | 145 | 1-19 | odorant binding protein 2 | AGR39565 | Agrotis ipsilon | 40 | Yes | PBP_GOBP | ABP-I | 60.39 |
| MsOBP9 | c24094_g1 | 96 | 1-22 | odorant binding protein 6 | ALZ45421 | Athetis dissimilis | 71 |  | PBP_GOBP | ABP-I | 49.75 |
| MsOBP10 | c23134_g1 | 144 | 1-23 | odorant binding protein 9 | AEB54592 | Helicoverpa armigera | 49 | Yes | PBP_GOBP | ? | 1410.06 |
| MsOBP11 | c23549_g1 | 145 | 1-25 | odorant binding protein 4 | ALZ45420 | Athetis dissimilis | 86 | Yes | PBP_GOBP | ABP-II | 357.23 |
| MsOBP12 | c24343_g1 | 142 | 1-21 | odorant binding protein 2 | AEB54586 | Helicoverpa armigera | 87 | Yes | PBP_GOBP | ABP-II | 2033.5 |
| MsOBP13 | c25075_g1 | 145 | 1-24 | odorant binding protein 12 | ALZ45424 | Athetis dissimilis | 82 | Yes | PBP_GOBP | ABP-II | 85.14 |
| MsOBP14 | c23825_g1 | 140 | 1-19 | odorant binding protein 10 | ALZ45423 | Athetis dissimilis | 72 | Yes | PBP_GOBP | ABP-II | 61.86 |
| MsOBP15 | c24885_g1 | 139 | 1-18 | odorant binding protein 8 | AEB54589 | Helicoverpa armigera | 87 | Yes | PBP_GOBP | ABP-II | 239.47 |
| MsOBP16 | c21911_g1 | 141 | 1-18 | odorant binding protein 13 | AEB54588 | Helicoverpa armigera | 89 | Yes | PBP_GOBP | ABP-II | 3644.63 |
| MsOBP17 | c17143_g1 | 137 | 1-20 | antennal binding protein X | CAA05508 | Heliothis virescens | 89 | Yes | PBP_GOBP | ABP-II | 11662.1 |
| MsOBP18 | c29119_g1 | 138 | 1-17 | odorant binding protein 5 | AGR39568 | Agrotis ipsilon | 72 | Yes | PBP_GOBP | Minus-C | 397.92 |
| MsOBP19 | c25074_g1 | 133 | 1-16 | odorant binding protein 9 | AGH70105 | Spodoptera exigua | 90 | Yes | PBP_GOBP | Minus-C | 530.24 |
| MsOBP20 | c27420_g1 | 153 | 1-17 | antennal binding protein 7 | ADO95155 | Antheraea yamamai | 33 | Yes | PBP_GOBP | Minus-C | 248.51 |
| MsOBP21 | c25764_g1 | 168 | 1-20 | odorant binding protein 4 | AKI87965 | Spodoptera litura | 74 | Yes | PBP_GOBP | CRLBP | 2368.19 |
| MsOBP22 | c26026_g1 | 183 | 1-17 | odorant binding protein 18 | AKT26496 | Spodoptera exigua | 61 | Yes | No hits | Plus-C | 296.72 |
| MsOBP23 | c23916_g1 | 146 | 1-16 | odorant binding protein 16 | AFI57165 | Helicoverpa armigera | 66 | Yes | PBP_GOBP | Plus-C | 32.14 |
| MsOBP24 | c25874_g1 | 197 | 1-17 | odorant binding protein 19 | AGC92793 | Helicoverpa assulta | 60 | Yes | No hits | Plus-C | 118.81 |
| MsOBP25 | c26853_g1 | 334 | 1-20 | odorant binding protein 9 | ALD65883 | Spodoptera litura | 79 | Yes | PBP_GOBP | Plus-C | 32.11 |
| MsOBP26 | c29915_g1 | 184 | 1-20 | odorant binding protein 10 | ALD65884 | Spodoptera litura | 98 | Yes | No hits | Plus-C | 9.3 |
| MsOBP27 | c24197_g1 | 164 | 1-16 | odorant binding protein 16 | ALD65890 | Spodoptera litura | 57 | Yes | No hits | Plus-C | 957.35 |
| MsOBP28 | c27900_g1 | 248 | 1-22 | odorant binding protein 1 | AKI87962 | Spodoptera litura | 85 | Yes | PBP_GOBP | Plus-C | 209.7 |
|  | c22587_g1 | 261 | 1-20 | odorant binding protein 3 | ALT31633 | Cnaphalocrocis medinalis | 38 | Yes | JHBP |  | 117.63 |
|  | c30753_g1 | 252 | 1-19 | odorant binding protein 23 | AKT26500 | Spodoptera exigua | 82 | Yes | JHBP |  | 192.08 |
|  | c28009_g1 | 236 | 1-18 | odorant binding protein 7 | ALT31637 | Cnaphalocrocis medinalis | 36 | Yes | JHBP |  | 92.23 |
|  | c27577_g1 | 238 | 1-19 | odorant binding protein | AOG12855 | Eogystia hippophaecolus | 56 | Yes | JHBP |  | 273.41 |
|  | c29527_g1 | 237 | 1-19 | odorant binding protein 25 | AKT26502 | Spodoptera exigua | 62 | Yes | JHBP |  | 1069.56 |

>MsPER Period c31643_g2

MDNLDDSENNAKISDSAYSNSCSNSQSRRSHSSKSTHSGSNSSGSSGYGGKPSTSGSSNNLLPPKKEKEPKKKKLLPQIETLVPDVVVEEVRAPEPAPTFDAPKEEKKLDVIPALPPAQTENGVENMDICVPELSTLQDEVVSGALSQVIPASSFVSGRALQASYCSVQQSKRYQVIGRYEMRCESSEALRCKDGFSCVISMHDGVVMYATSTLTTTLGYPKDMWIGRSFIDFVHPRDRNTFASQITNGLAVPKNANGTQEKVQSPANAVSTMVCRVRRYRGLTTGFGVKERVVGFMPFLLKLTFKNISDEEGKVIYLVIQATPFFSAFKTPNEVVTKAIPFVIRHAANGHIEYVDPESVPYLGFLPQDLADTDALLLYHPEDLLYLRTVYETIVKEGGLPRSKSYRMVTQNGDYIRLETEWSSFINPWSKKLEFVIGKHHILEGPTNPDVFEAPEPEKPTKGCDEEKNKAKMLRENIIRTMNEALTKPAEIAKQQMSQRCQDLASFMESLMEEPPKTDDELRLEIQYPDHSYYERDSVMLGGISPHHDYYDSKSSTETPLSYNQLNYNETLQRYFDSHQPITYDNFGTATGEDIFAPKERPSKYAPCLSPMAQHSRDSGDITCSSDSNGMIMISNSPVPAGDYQPIRLTESLLNKHNETMEKELLKKHRDTRSTSKEFREKKSNETRKKKKEHLARCNASYHPTSAGATEVHQPHGLKRASMTTEAEPTSHKHHCPSPRQARRNKRQAPPATFTQPPEPVTTTVASNPWPSPPANNMNAFILGVGIPPQMSIMSPMPHAMLPMHTMPAVPGMYPMYYAQPAPQPMPTTSGHHYAPSTSQQHQFQAPIMMYGHPMYGQPLMYSPLTHPMSYPMQQSHMMAQSMQQHYSNTMNPLGLTSSNYEEACKPSLTLRPGKVQGSSWRDKKRAEFHGASKTDNGTADSLSSNALNRESGPHKSQNGNEDSTIYSTDIQSSNSLSHKSTEAATNTPPPRESRLNKLNRLGNSEETPDKTDGESSYSSFYSSFFKTESGSAEDSGDGKNGNKPPVRSPLNRVPQPAKKPAAWRKMEPPWLEQVCVTSELVYKYQIHTKSLEETLTADKQKMKNLEQPSLVNEQLSQLYLDLQLEGVAARLTLEEGITSSSSSGEETTTNVKTSRRKREYSKLVMIYEEDAPLPPPSDDAADAVASSSSTS

>MsCLK CLOCK c30631_g2

FEVLGTSGYDYYHFDDLEKVVSCHEALMQKGELTSCYYRFLTKGQQWIWLQTRFYITYHQWNSKPEFVVCTHRVVSYADIAKSMKQEGVDGDTVSEAEANRGVMKEAPSDDAMVSMSPSYMSEASDAFGSSSYQQLSQVSPASVKSASAGSTTGTVATAGTVATAGASWTRASLVRFTGSDTGSLSGESRSSQRNSMREQPMAHKPADTAPAPQHGMGAQYLDPAPYVSAVGMPGVLPLSIPPMPVIVSSDQAQMQLQRKHEELQQMIVRQQEELRQVKEQLLLARLGILQPLINVQGPYVNPEDIQGNQRLPAQIVYEGGTPRAITGYPPQHPPHSGAHHQHMPQ

>MsCYC Cycle c31074_g1

MARELARVHDYYEPLHELQPAHHYHHYDLHHQASASAAPSYELTAGGGGACAESAGAHAHAPHPPPMQQASHQHHHDPRKRKTSHYGSDGYELPGCEAPLGAPQQPLLTSASATRKRKPSSYGTSSAYDDDGGDDARSTRTLPDKKQNHSEIEKRRRDKMNTYISELSAMIPMCGAMARKLDKLTVLRMAVQHLRSVRGALSACPLSARPRPAFLSERELNQLILHAAHDCFLLVVGCDRGRLLYVSASVRQMLHYDQTELLGQSLFDILHPKDVAKVKEQLSSSDLSPRERLIDAKTMLPVKADMQAGASRLCPGARRSFFCRIKCKANPQAVQEPAPVKEDPEPSAKLRKKSNEKKYCVVQCTGYLKSWAPAEMSEAGSSEAADDVDAGSLSCLVAVGRALPDLAPEPPAPAPEPPQRQLQYVSRHAPDGKFLFVDQRVTLALGFLPQELLGTSLYEYVAGPELGAVARAHKAALLSREPLRTPPYTFRRKDGSLARLHTHFKPFKNPWTKDVECLVANNTVVTAAHEDGIDAGQTACDLYKQKADVEMQRLIESQVESHKIGSAIAEEALRRSSSEFSPELPADLLQDAVFNQNWPLKANVLYDQASLVDNILGAELTGYGQVRNNVPLSAAASGSPPAQAELTGSPPPTSPPLPPLGLDGNGEAAMAVIMSLLEADAGLGGPVNFSGLPWPLP

>MsVRI vrille c29769_g1

MVAEFILSQQQLLSGAGGGGLGPAPPPSRAPPPNRGRQSVSPYPMDHSQGPVGSPGDPNDYSPFEFNKRKEFFGQRKQREFIPDSKKDDGYWDRRRRNNEAAKRSREKRRFNDMVLEQRVVELSKENHVLKAQLDAIKEKYGICGETLISIDQVLATLPTCDQVLCVTKRSKLTTNTLFPAPPPPPPAQPASPPSPPPQRQPEPYQERLPAPEAYYPHPAHYEPPGSVLNLSRSRRVPSPYELSSLSGSGDETAEQYAPENNGLPLKLRHKSHLGDKDVASALLSLQHIKQEPGPRSSPSWDGEGSSDERDSGISIGAEYRPQRPEDRLAAEEEDAHLKAELARLATEVATLKNMMHQNKSRAHEH

>MsTIM timeless c32116_g1

MVQLIALLYKDQHVVTLHKLLNLWIEASLSESSEDNESNTSPPDRGSDSSPMCTSEPTSDSSDMGGSGKSNDDPNSVNKDWDASSQMNNTSENGNVPFRRPEEKESDTEDKNNTTASPNDNVVQSDNGMSTTCSSGVQSKANSQGQSPGNTSEKKPVISETSDCGYGTQIENQESISTSSNEDELPSKKPVHQKPHNPKQRVNNNKNRAGVSMQERKRKKIVKRGKSNIINVQGLSHKTPTDDDISNVLKEFTVDFLLKGYNSLVQTLHDQILSNHQLEIDTSHFFWLVTYFLKFATQIELDLENVNSVLSIDIVSYLTAEGVNLCEQFELAIKLDGNDLKPNIRRLHLVVTAIREIIQAIDVYKKLPHICKEDQDALLKLQLKMCETEELRSLLVLLLRHYNPKYHSKQYLQDVIVTNHILLMFLDGPMKSPEYNGATNMVDHIRQFASSDIMYQYGLLLEDYADNGEFVNDCVFTVMHHVGGELESLITLFQPKILKTFTTIWKSEFEICDDWSDLIEYVINTFIKKPHSLQSRILIESFEEVKIVQKPVPAPQASPEATQAGDKKKPTNSSASCTSKGKAESRWTEDELSSLSWNYMQCSALPDVVGEIISLYKEDGIIKSRDSVIKELYTQNLINKEDYDRYVKGETDRNVKTVQVMKEMRDDEINKLCEQLTQDGKSKFLDWVQKVLLETCSAKIYLDKLARKSESENNANETKLTTLKKSNEFHVLSPVSYHSILLNQSVPLVPWNCKQAAVCKDLKFLQLLHKLGFHMPVDSGKVFIRIPQFWSSDFLYDVAGKISPIDKSKLKFSVNDISDSGISNSMQQESLLLLPENTKDITMMESPENFYQIHKQKHLATMVNFTPMPGSAFNSDGSDVEKHNWLELVQKSQEYKIALNLSSSIINNEEDEGSDIVMHMAGKDAAVSVPPLLLAAPVTISSTATITSTTFPGTGLMMPTIMSIKQETTHFNVPVPDSEHFSVCETASVASDLTRMYVSDEDEKPETVRPLIKMNPIIDENCGDLDEDNNSETRLRVNFFSPF

>MsCRY1 cryptochrome 1 c31435_g1/g2

MLGGSVLWFRHGLRLHDNPSLHCALEEKGFPFFPIFIFDGETAGTKLVGYNRMRYLLEALEDLDSQLKKHGGRLIMLKGKPNVVFRRLWEEFGIRRLCFEQDCEPVWRARDDSVKAACKEIGVVCKENVSHTLWEPDTVIKANGGIPPLTYQMFLHTVATIGDPPRPVSNVDFTGVKFGSLPECFYQEFTVFDKTPKPEDLGVFLENEDIRMIRWVGGETTALKQMQQRLAVEYETFLRGSYLPTHGNPDLLGPPISLSPALRFGCLSVRSFYWALQDLFRQVHQGRLATQSASHVIAGQLIWREYFYTMSVNNPNYGQMAGNPICLDIPWKEPQGDELQRWVEGRTGFPFVDAAMRQLRTEGWLHHAARNTVASFLTRGTLWLSWEHGLNHFLKYLLDADWSVCAGNWMWVSSSAFEALLDSGECACPVRLGQRLDPSGEYVRRYVPELARMPVQYIYEPWKAPIDVQERATCIIGKDYPGPVVNHLVAAQKNKNAMKELRHILQKAPPHCCPSSEDEIRQFMWLNE

>Ms cryptochrome 2 c31387_g4

YGRVRDHNIMSKCREVGITVTSRVSHTLYKLDQIIERNGGKAPLTYHQFQALIASMPPPPKAEAPITAQTLNGATTPVTDDHDDRFGVPTLEELGFETEGLKPPVWVGGESEALARLERHLERKAWVASFGRPKMTPQSLLASQTGLSPYLRFGCLSTRLFYYQLTELYKRVKRVRPPLSLHGQILWREFFYCAATRNPNFDRMEGNPICVQIPWEKNQEALAKWASGQTGFPWIDAIIIQLREEGWIHHLARHAVACFLTRGDLWISWEEGMKVFDELLLDADWSVNAGMWMWLSCSSFFQQFFHCYCPVRFGRKTDPNGDFIRRYIPALKNMPTRYIHEPWVAPESVQQSARCIIGRDYPMPMVDHAKASQVNIERIKQVYAQLAKYKPQGTLNPNAVQRPNVMQSSPSPNSIITSINQSNYLCSQAPEPPTTTPQIIPYKDNDVVFQKPMNHRSMKPSFKQVVIVQKKQNTNVIQTVTQSKEKYIVNGQPAKQENYDFKNLVINNYVQGYSNNQEIFQNQQTNKNELFAQPTLKINSFNYEKQKFFLSAFTDNGVRRSAVHDEVPPPFTVALNHDTNMPYIRENKNEK

>MsSGG shaggy c28955_g1

MSGRPRTTSFAEGSKSVRKTFDNQPPKPPLGGVKISSDVDTPKKNELASEKKASSHRKDGSKVTTVVATPGQGPDRPQEVSYADMKLIGNGSFGVVYQAKLCDTGELIAIKKVLQDKRFKNRELQIMRRLEHCNIVKLKYFFYSSGEKKDEVYLNLVLEYIPETVYKVARHYSKDEQTIPISFIKLYMYQLFRSLAYIHSLGICHRDIKPQNLLLDPKTGVLKLCDFGSAKHLVRGEPNVSYICSRYYRAPELIFGAIDYTTKIDVWSAGCVVAELLLGQPIFPGDSGVDQLVEIIKVLGTPTREQIREMNPNYTEFKFPQIKSHPWAKVFRACTPPDAISLVSRLLEYTPGARLSPLQACAHSFFDELREPAARLPNGRPLPPLFNFTEYELSIQPSLNDFLKPRAGAAPAADAAAGAAPADEAPGSCSAEPRA

>MsPDP1 PAR-domain protein 1 c21770_g1

MSDRDRSSPSLTDTPLKTYIGRHDDDYPLINGNDFLSHGASFNKKYGKSVNSKDPLEDKKDDSDLWEAQAAFLGPTLWDKTLPYDPDLKYADLDEFLSENGMSGEGLGGAHLGGAAFGPALGLPAPVTKRERSPSPSDCMSPDTINPPLSPADSTFSMASSGRDFDPRTRAFSDEELKPQPMIKKSRKQFVPDDLKDDKYWARRRKNNMAAKRSRDARRMKENQIAMRAGYLEKENMGLRQEVELLKKENHILREKLSKYADV

>MsCK2a casein kinase II alpha subunit c17691_g1

MAVPSRARVYADVNSQRPREYWDYESYVVDWGNQEDYQLVRKLGRGKYSEVFEAINITNNEKCVVKILKPVKKKKIKREIKILENLRGGTNIITLQAVVKDPVSRTPALIFEHVNNTDFKQLYQTLSDYDIRYYLYELLKALDYCHSMGIMHRDVKPHNVMIDHDHRKLRLIDWGLAEFYHPGQDYNVRVASRYFKGPELLVDYQMYDYSLDMWSLGCMLASMIFRKEPFFHGHDNYDQLVRIAKVLGTEELFEYLDKYHIELDPRFNDILGRHSRKRWERFVHSENQHLVSPEALDFLDRLLRYDHYERYTAREAMDHPYFYPIVKEQGRMVSSNSPTPNALQGPISTTE

>MsCK2b casein kinase II subunit beta c25884_g1

MSSSEEVSWISWFCGLRGNEFFCEVDEDYINDKFNLTGLNEQVPHYRQALDMILDLEPEYFLDDDLDDNPNQSDLVEQASEILYGLIHARYILTNRGIGQMLEKFQAGDFGHCPRVYCECQPMLPLGLSDVPGEAMVKLYCPRCMDVYTPKSSRHHHTDGAYFGTGFPHMVFMVHPEYRPKRPASQFVPRLYGFKIHPLAYQIQQQAAANFKAPLRSLSYNNGKR

>MsMET methoprene-tolerant homolog-2 c40384_g1

ENPREVRNRAEKMRRDRLNQSVAELAMMVPPVVAARRKIDKTTVLRLTAHYLRAHQYVFGDSIGQTAQPFNPSSMLKVLSMFNGFLITTTYRGIVVVVSQNVNQYLGYSELDLLGQNLLTITHEGD

>MsSLMB Slimb c39876_g1

RKVRTDSLWRGLAERRGWIQYLFKPKPGSQHPSHSFYRQLYPKIIKDIQSIEDNWRMGKHNLQRINCRSENSKGVYCLQYDDNKIVSGLRDNTIKIWDRKTLQCVRELQGHTGSVLCLQYDERAIISGSSDSTVRVWDVNTGAMLNTLIHHCEAVLHLRFCNGMMVTCSKDRSIAVWDMTSTTEIM

>MsDBT double-time c31342_g1

MLNTKMELRVGNKYRLGRKIGSGSFGDIYLGTNIVTKEEVAIKLECIKTRHPQLHIESKFYKLMQGGVGIPAIKWCGSEGDYNVMVMELLGPSLEDLFNFCSRRFSLKTVLLLADQLITRIEDIHYRNFIHRDIKPDNFLMGLGKKGNLVYIIDFGLAKKYKDARTLQHIPYRENKNLTGTARYASINTHLGIEQSRRDDLESLGYVLMYFNRGSLPWQGLKAATKRQKYERISEKKLSTPFDELCKNHPIEFQLYLKYCRRLRFEERPDYSHLRQLFRTLFHRQGFTYDYVFDWNMLKFGGQRGNYQSGGNDRTLRRHEQNQEQENTAGAAGQPSTTVAAISEWR

>MsTH tyrosine hydroxylase c17613_g1

MAVAAAQKNREMFAIKKSYSIENGYPSRRRSLVDDARFETLVVKQTKQSVLEEARARANDSGLDSDFIQDGIHIGNGDNSPTVEDGTQQDETKNGHLADADIGDDAGKTDDDYTLTEEEVILQNAASESPEAEQAIQQAALLLRMRDGMGSLARILKTIDNYKGCVQHLETRPSQLTGVQFDALVKVSMSRINLLQLIRSLRQSTSFAGVNLVSENNISSKTPWFPRHASDLDNCNHLMTKYEPELDMNHPGFADKDYRERRKQIAEIAFGYKYGDPIPSITYKESENATWQRVFNTVLDLMPKHACKEYKVAFGKLQAANIFVPQHIPQLEDVSNFLRKHTGFTLRPAAGLLTARDFLASLAFRVFQSTQYVRHANSPFHTPEPDCIHELLGHIPLLADLSFAQFSQEIGLASLGASDAEIEKLSTVYWFTVEFGLCKENQQLKAYGAALLSSIGELLHALSDKPELRPFEPASTSVQPYQDQEYQPIYYVAESFEDAKDKFRRWVSTMSRPFEVRFNPHTERVEVLDSVDKLETLIWQLNTEMLHLTNAVKKLKDSQFE

>MsDCC1 dopa decarboxylase 1 c31937_g1

MSDQDLKLKFETMEAGDFKDFAKAMTDYIAEYLENIRDRQVVPSVKPGYLRPLVPEQAPEKAEPWTAVMADIERVVMSGVTHWHSPRFHAYFPTANSYPAIVADMLSGAIACIGFTWIASPACTELEVVMLDWLGQMLGLPESFLARSGGEAGGVIQGTASEATLVALLGAKNRTMLRVKEQHPEWTDTDILSKLVGYCNKQAHSSVERAGLLGGVKLRALQPDGKRRLRGDTLKDAIEEDVRNGLIPFYVVATLGTTSSCAFDNLEEIGEVCSSKNIWLHVDAAYAGSAFICPEYRYLMKGVDKADSFNFNPHKWMLVNFDCSAMWLKEPRWIIDAFNVDPLYLKHDQQGSAPDYRHWQIPLGRRFRALKLWFVLRLYGVENLQKHIRKHIALAHLFERLCSADERFEIYEEVTMGLVCFRLKGGNEQNEELLRRINGRGKIHLVPSKIDDTYFLRVAICSRFSEESDIHISWEEVKASADEILKAR

>MsDDC2 dopa decarboxylase 2 c26899_g1

MDAQQFREFGKAAIDVLADYVENIRDKDVLPSVEPGYLLNALPEDAPEQPEDWQEILKDFNQSIMPGVTHWHSPRFHAFYPTGTSYASIIGNMLCDGLSVIGFSWLSSPACTELEVLTMNWLGKLLGIPEDFLHCSSGPGGGIIQGSASEATLVGLLAAKDKTVRRLIRNDPTLDEDEIKPKLVAYTSDQCNSSVEKAGLLGSMKMRLLKADNDGRLRGETLKNAFEEDRAQGLIPCYVIANLGTTGTCAFDPLYELGPVCNEADVWLHVDAAYAGAAFMCPEYRHLMRGIELSDSIDVNAHKWMPVSFDCSAMWVKDGYDLVRAFDVQRIYLDDVKTENKIPDYRHWQIPLGRRFRSLKLWTVMRVYGAEGLRKHVRDQITLAQYFAKLVRADERFIVEPEPSMGLVCFRLKEGDIITKKLLESLTAKKKVFMVAGTYRDRYVIRFVICSRLTVKEDVDYSWKQIKDETDLLYTDKIHTKAQIPAIDQIVAREICEKSK

>MsEbony ebony c31839_g1

MGSIPRVSVVSGARTLVPAAPLPKHLAHLAGSDNTALVYADETCNVRVSYAELEARTNSLARAISSRARPSGANRDGDYVIAVCMQPSHSTITTLLATWKAGAAYVPMEPSFPQARVSHILKDAEPALIVYDETANPSMFSGSGVPAVSYEELAHEASGLSADAVQDMEALAPSRSESIAIVLYTSGSTGIPKGVRLSYSAVCNRLWWQFHTFPYSDSEVNCVWKTALTFVDSVCEIWGPLLHGRTLLILSRETTRDPQKLVNVLADNQIQRLVLVPTLLRSILMYLTLKPSERPLQNLKLWVCSGETLSKDLAAQFFKTFGNHNGFKLANFYGSTEVMGDVTYYVLESASQVDIHPTIPIGVPLDNSAVYLLDEEMNPARESEPGEVWVSGRNLAAGYVGGQGADKFVDNPHAAHPDFGRLYRTGDFGVLEKGVILYAGRTDSQIKIRGHRVDLQEVDRAVAGVDGVDKCVVLCYGLDRGNPEILAFVTIKPEARIAAQHIEAALKNSLTSYMIPQVIEVESVPLLVNGKVDRQALLKMYENTNNNDDSEIPLDIDYSSIPASELRAATVLFETVGEVLGRSARAAISARAGFYELGGNSLNSIYTITRLRDKGYYIDISDFLGAANLGEVLSHMSTSPHSEADDSELFTAEPLADEHKQQVIEMIVSSFYNKAELEQFLKHEIDTNDYAVLISDTWPALINANLSVVLKNPSGSPVAVALNFDARDEPDVESEETGGLMKIMLFLEFVESSVRDSMLPRGKGTILHSFMMATAESLTPRENVAAIRALENATMNVAKERGFLGVFTTNTSPLTQQLGTDVLGYQTLLDYQINQYVDPNGDRIFGKAPDDMRAVVCWKPLE

>MsTan tan c30840_g3

MRELNGVSCEKPLGRRATVPVIYVQGSHYDVGYDVGRTFSSVIKSFISSYENLRDFEKEYKTDTGRNAYDKTLTNMRKKFPYYVKEIQGVADGAGVPFYQLFLLQMDDLIGNVNDNHIPRNDTGGCSSMAFKNPHATLLGHTEDAFHETLNHFYIMSAHIIPTPEDREAGAVEERFASLCYAGHMPGYTMGYNQNGLVFSINTLSPLILKHGNTPRTFLTRAMLSAKNFSQAEQILRDEGLGIGNGFSVNMIWTNNWGDRQIYNVEVSPDLKADRSHLNIQKYDKDLLVHCNKYQRTNVTEVTGPIIDSSVERLQVIHGYPKPRDRHDLAQILSDTTGKNFRVFADRPDDIIMTIAAGIFDLDKRTWSIYINKPKLSEPVAVLPITFSCLDY

>MsaaNAT1 arylalkylamine N-acetyltransferase 1 c31939_g1

MAVTSARNIVNLKETGHFERENARIDKYTLADKLPEQLAALDISKASQTSYHVRTITAKDKERTLDFLRRFFFRDEPLNLAVNLLETPTSRCLELEEYASSSIDEGVSLAAVDENGEFVGIVLNAIVHRDDVDDSDKVEQCPHHKFRRILRLLTHLSKEAKIWEKVPASENAMMDLRIASTHPDWRGRRIMKVLAEESERIAKAHGACAIRMDATSAFSARAAERLGYSKVYSVPYADILDGPQPQPPHFEASVYLKKF

>MsaaNAT2 dopamine N-acetyltransferase-like c29401_g2

MAGYEVKPVEAGDTEAILVLLRKTFFIDEPMNQAVGLCAEGTCNELEDYCKQYLPGDGWSFKAVDKEGNIVGVMVSGICDLKEPEVGSDYTSLAKTCSNPKFARILYVLGQREAGAKLWDKFPDEKEVLDVKIAATDPNWRKKGIMNALLFETEKLAKKRSVRILRMDTSSAYSAMSAEKLGFTCMFSAAYVDIKLDGRPIIVPEPPHVDDRVYTKILYERNA

>MsGTPCH GTP cyclohydrolase I c27740_g1

MAAVNGKDSDPIVPPSSLIRRVSSRSVRNSISEEKENGDGPVKGSFVSKYKKAPEALKSFHLTDAESELEVPGTPMTPRTSTTPGHENCTFHHDLELDHRPPTREALLPDMANSYRLLLTGLGEDPERAGLLKTPERAAKAMLFFTKGYDQSLEEVLNNAIFDEDTDEMVVVKDIEMFSMCEHHLVPFYGKVSIGYLPQGKILGLSKLARIVEIFSRRLQVQERLTKQIAVAVTQAVRPAGVAVVIEGVHMCMVMRGVQKINSKTVTSTMLGVFRDDPKTREEFLNLVHSK

>MsPPO1 prophenoloxidase 1 c10055_g1/c39766_g1

NLLLFFERPTEPCFMQKGEDKAVFEIPEHYYPDKYKAVTTTIANRFGDDAGRTIPVRNIALPNLNLPMELPYNEQFSLFIPKHRTMAGKLIDIFMNMRDVEDLQSVCSFCQLRINPYMFNYCLSVAILHRPDTKGINIPTFAETFPDKFMDPKVFRKAREVSNVVTSGNRLPVTIPVNYTANDTEPEQRVAYFREDIGIN

>MsPPO2 prophenoloxidase 2 c30210_g2

MADVVESLKLLFDRPNEPLITPKGDQKAVFQLSEKLVPPEYASNGVELNDRFGDDAGEKIPLKTLTNYPSFSKASQLPSDADFSLFLPKHQEMATEVIDALLGVPENQLQDFLSTCVFARANLNPQLFNYCYSVALMHRDDTKNVPIQNFAETFPSKFMDSQVFQQAREVAAVVPQTVARTPIIIPRDYTATDLEEEHRLAYWREDIGVNLHHWHWHLVYPFTASQRSIVAKDRRGELFFYMHQQLIARYNCERLNNSLKRVKKFSNWREPIPEAYFPKLDSLTSSRGWPPRQANMRWQDLKRPVDGLDITINDMERWRRQIEEAISTGMVTHSDGSRSALDIDKLGNMLESSILSPNRELYGSIHNNGHSFSAYMHDPTHRYLESFGVIADEATTMRDPFFFRWHAWIDDICQSHKESSYVRPYTRSELENQGVQITSVACETDGYPANTLNTFWMSSDVDLSRGLDFSDRGPVYARFTHLNNRPFRYVIKCNNTGSARRTTCRIFIAPKFDERNLVWALTEQRKMFIEMDRFVVPLNAGENTITRLSTESSVTIPFEQTFRDLSAQSNDPRRPNLAEFNFCGCGWPQHMLVPKGTEAGAAYQLFVMLSNYDLDSVEQEDGSPMSCVQASSFCGLRDKKYPDRRAMGFPFDRPSSTATNIEDFILPNMGLQDITIRLLNQTEPNPRNPPSSS

>MsLAC1 laccase c31746_g1

MESEKEVAQVREVSMVKPNYMLMSLQRILVLTVILAGVLVVVHYTPMPEEYFENCDRECHELDWPMICRVKLVIEVYKTLSKSCGSCTEKDGGDCPATCISADGRERGVLVANRALPSPTLHVCHNDILVVDVVHRAPAHALSIHWRGQPQKETPFMDGAPMLTQCPQPAYTTFQYKFRASAVGTHMYHAHSAADAADGLAGAFVVRQSARLDPIKSLYDVDASEHTIYVSEWGHSMGPLAGVISRIPNAESLLINGKGKSSESPDAPQSSFNVEYGKRYRFRLAYGGGSKSCPIIFSIDHHVLEVVTLDGHQIEPQRVNSVELGRGERVDFIVDAKKVPGVYKIRVAAVKSCQDNLEGVANLVYEDKKQKVLHKDDDKVDTEVSRELTTVTSDRCESDNVVCLSEIHAATKLPVELTENLDKTIYVPFNYSTRQISAKRVESWGQTDGHRFTYPASPLLTQGGDVGANQFCTKEPGHGDECVHVKHIPLGATVELVMFDQGGESDHIFHLHGYSFHVVGMRELHRSFDSETIKKMNEEGTLFNKNLVDPVEKDTIVVPKFGVVALRFKADNPGFWMMRDERSSHWTRGLDFILKVGEQSDFVQAPPDFPKCGSYVGPEYFLI

>MsLAC2 laccase c32155_g1

MNHGNYGQVVSAIFLLLLINLTGIKTQTETAQSFETTTFDFNTQNADDAKEGTQQTNNPYVFVLEDDEQNATISEDFHHKAPESASRVLVAIKPNGTNVLKIEPNRKSNPPAKHPVRFMQDEKETVISKNVKSKDAKIRAHVRYDEVTGELIDGDHPCHRECREGEEPMICYYHFVLEWYQTMSKACYDCPYNLTDCSRPDCIPADGMNRPLNVINRKMPGPAVEVCQHDRVIVDVENDLMTEGTTVHWHGQHQRGTPYMDGTPYVTQCPILPETTFRYQFNATHAGTHFWHSHSGMQRADGAAGVFVVRKPKSQDPHGRLYDYDRSEHVMLVTDWIHDLAMGMFTDHHHSKGDNKPPTLLINGVGRFKVFNETAKPMYMQAARFNVEQGYKYRFRVINAEFLNCPIELSVDGHNITVIASDGYDLQPITTTSLVTYAGERYDFILEANNEIDNYWIRYRGLMDCDEIFTKAKQVGVLHYEGAMDLEPPGDPTWEELHNDGLQLNALNKGEEDEETISVAEMRSLEGHDDSLKEVADYQFYIAYDFYAKNNSHFHRSPYYGYYQVPAKKNRLYTPQLNHISMKLPSSPLMLARPSMDNFCNASSIDESCTEGYCECSHVLAVKLNSVVELIIVDEGVTFDANHPFHLHGHSFRVVGLRRLAKDTTIEEIKAYDKAGLLKRNLKNAPIKDTVTVPDGGYTVVRFKADNPGYWLFHCHIEFHVEIGMALVFKVGEHKDMAPIPRDFPTCGSYLPDNMLEHATTEKARSEEDVITISHWWPVVFVNGTSSSTNVYASGLSILFSILLLALNLKS

>Msyellow-c c30567_g2

PPLPLPYFNTSQYYHTSNMRAVGFFVALAVVTVCQAATPQLRFAWKQMDYTWETPESRANAISEKRFIPENNLPLGLARWKNKMFVTIPRWKAGVASTLNYVDVDGPQDQLLKPYPSFKDNFVPDSAKELPSNNSLISVFRVFVDECDRLWVIDSGMADIFGEGNQVNGPSIVIFDLKTDQLIHRYQCKAGDMKEDSFFANIIVDVNKDTCDDAYAYIPDLGGYGVVVYSLKQDDSWRISHHYFHFEPLAGSYNVSGIEFQWTDGVFGLALSEPREDGYRTMFFHAFSSTKEFCVSTELLRNYKSIDKIEAFHDFKLLGDRGERTQSAASYYDPKTQVLFYTQVNRDGVACWNSNKPYTPDNNPLIITDPKLFEFPNDMKVDNEGTLWVLTDKLARFLYRTMDPNEVNYRIFSINTAEAVEGTACQ

>Msyellow-f1 c31466_g1/g2

MGAEKVGNRLFVTIPRRRFGIPATLNYIDLSVHGRTRSPPLTPYPDMARSRSLISVYRTRADECGRLWTVDTGTIEIPNNPRYLQTPAIVVFDLKTDRQILRYQLKSSDLPGNNTPTGLASITVAIKNGDCSDAYAFISDVVTYGVIVFSLKQNDSWRQQHNYFHFNPTAGNLRVAGQSFTWNDGVFSVAVGPEGPDGCRPTYFHPMVSSQEFSVSSCELESREANQTAYTVLGDRGTNSQCTMHDLHAPTNVMFFAEVGRDSVSCWNTERPLNPDNMEILAQDSQRLSYPSDLHVTDDEVWVTANTLPRFIYSRLDTDEYNFFIYRAKVRDLISGTVCAGNARPSPGFSSSYGGYHRS

>Msyellow-f2 c29411_g1

MFSNKIILLLVLISYCNGQYFIPNNHFSDMKQVFSWNQIGYNFDGVKYVKDEPLQKIVGGIFFAEEREEREEDERFFIQYNNIPIGFEVFHDKVFITVPRRRYGIPSTLNYVDLKSSKSSSPLLKPYPDAESTNNFVSVYRPRVDACERLWMVDTGLLEVPNNFLQIRPPHIVVYDLKTNKEVLRYEIPADVLVNGTTSGLTSITVDVTPSTCDDAYAYINDLARNGLIVYSMKKRDCWRFSHPSFVYDVGATDFVVAHQTINWHDGIFSIALSDPDSTGRRTAYYHPLVSTQEFSVSNEVLKDKNANFETGFKLEGVRGAFSQSGSHDFHSATQTLLYANVAQDSIMCWNTRTPLTSKNSVPVAQSHSKLVYISDLKVIGDDVWVLVNQIPTFIYSQFNITEPNFFVHKGKVRDLIKGTACDK

>Msyellow-d c30885_g1

MSQGIERFFLLSYCLACCWPGLGAKSDLRVVRQWPELQFVFPSETARQIALENRYYVPGNSVPIDVDVHHRQGREGSRIFVTIPRFDEGRPITLGTVDEQGNIVAYPDYSWHDNQGHNCLGFTSVFRVAIDVCQRLWVMDAGKIGDDQVCPPQLLAFDLATDTLIYRHIVSNHSYSDASLFITPVVDVRPRGPGDCANTYVYVADVSAYGLLVVDVMRNRSWRTTHRTFYPFPSRGTFTIDGESFDLMDGVLGMALSPFQHGRERFLYYHSLASTTENVVNTRILRNDTYEDDPNIDPDAVYVFPEERSTQSAAEAMDSNGILYFGLMEPPSIWCWNTATQFSQRNFHEVAVDRETLQFASGVKVVMNAKGEEELWVLTSSFQRVMTGSISSDRINYRIHAEKIKNILANSPCKDSPKGNKHGYHANLIDRAGDGHIRRPSRYGSASYL

>Msyellow-h c20824_g2/g1

MEKILLTVILTSIVTTNYGYIQIASRKPLGTLFRWKQIDYAYPTPQDRQDAINNGQFNATNVIPLGVERWKDRVFISTPRWKTGTPATLSSVPISSPTESPPLEPYPNWDWHNAGNCTGLTSVFRMNIDHCGVMWVLDSGQVEAFETPRQLCPPTLFAINLSTDTVVGRYPIPSEFVLQNSLITNLVVDSRDVRCRDLHVYIADAWRFGLIVFRHEDAAFWRFSHYSFYPEPLLSNYTLHGLNYQWSDGLFGMSLGKYQQGDRPLYYHSMSSSLEFVVSTSVIRDPTRVSNSVGEFSLLGDSRGADGQVSAAAVDRNGVMFFNLISRDSIGCWDTRKPYTNRNLVVVAQNNNTMIFPNDLRMDHEVPQMAWIITNRLPMYQFNLIDPNECNYRVMYLDPVAAVQNSMCQPEIRNISEDQDTIDS

>Msyellow-x c28958_g1

MLALLLLAVAAAAAQNDTYPRPPAPRREQFRVIYEWNAVDFEWKSPEDREASLNTSRYIPQNVLISGINFYQDDLFLTMPRMLDGVPATLATIPVTPTDTAPKLRPFPSWEQNAVGDCNALQFVQNIEIDKNGIMWILDNGRVGTLTQNPDPKCPPSLVLIDLKTGKNEMERIPFPSDSVNPNTTYLNDLVVDNRDGDYAYITDNSAVDPGIIVFRRRDKKSWKVRDTKSMTSVTEATFFRINGTTVNLPVNIDGIALGPQYRNEEGKVDRKLYYCPLSSFHLYAVNVSVLQNESLAQQGEGALRPYVVDLGSKASQTDGMKMDSTGVLFYGLIGNSTIAEWNTTVDFRVGQRTIARDPNYIQWVDRFTFDGKGNVYVVINRLYNFVKNQVSLSEVNYRILKSHTGSKSYVFSEDLEEPAAPQHGAAAAPALATSLALLALAHLLA

>Msyellow-b c31930_g1

MGGLKTLIILQLIYFCGCMDQLHIVYEWKQIDYEFPSPAARAQAIETKAFIPENNIPMGLEIYEDRLFITVPRWKSGVPASLNYINLKDNSTNSPKLIPYPSWAAHSIGMDGKPPEIVSPFRVRADRCGRLWVLDNGKMGNLETNVTKYPPSIIVYDLKTDNLLRKYVFPADQVKEDSGFANIAVEDADCENTYAYAGDLGKPAIVVYSWEKNESWRITHHFFHPDPLACDFSVKGFNFSWTDALFGLGLSAPNADNYSTLYFHPMASYDEFAVSTEYLRNQSLAEENFKAFKVLGSRGPNAQSSVSFVDPKTGVLFYSLVNLNAVACWKTSNKEYLMKNQGRIYMNEVTMVYPTDIKVDYNDNLWILSNRMPIWMYGTLDPNDVNFRVFSAPVVDAISHTACDITPRSDILDKFVNKVKNATNSLVAKIKQNSGGVAISPLSVVTVLMVSWIISLTI

>Msyellow c37756_g1

CPYSLNIYDLNTDQRIRRYVFRPEDIVASTFIANIALDEGRTCDDTFAYFSDELGYGLIAYSWQQNRSWRFSHGFFMPDPLTGDFNIGGLNFQWSAEGVFGITTSPIGSDGYRTLYFSPLASNTQFAVSTRILRDESKVKGSYKDFQVVGVRGANGHTTSKVMDEYTGVELFNL

>MsSNMP1 c31246_g1

MLLPKELKYSAIAGGVAVFGLIFGWVLFPVILKGQLKKEMALSKKTDVRKMWETIPFALEFKVYLFNYTNAEEVQKGAKPILKEIGPYHFDEWKEKVEIEDHEEDDTITYKKRDTFYFNPELSAPGLTGEEIVVMPHIFMLGMALTVNRDKPAMLNMVGKAMNGIFDNPPDIFMRVKALDILFRGIIINCARTEFAPKATCTALKKEGVSGLIIEPNNQFRFSIFGTRNNTIDPHIITVKRGIQNVMDVGQVVAVDGKPEQTIWKGACNEYQGTDGTVFPPFLTENDRIQSFSTDLCRSFKPWYQKKTSYRGIKTNRYIANIGNFADDPELQCFCPDPDKCPPKGLMDLAPCIKAPMYASMPHYLESDPALLNNVKGLNPDINQHGIEIDFEPISGTPMVAKQRIQFNLQLLKTDKIDLFKDLSGDIVPLFWIEEGLALNKTFVNMLKHQLFIPKRVVGVLRWWMVSFGSLGAVIGIVYHFRDHIMRLAVSGDTKVSKVTPEEGQEQKDISVIGAQAQEPAKINI

>MsSNMP2 c27191_g1

MLGKHSKLIFAVSIGFLVVAIIMASWGFQKVVDKQIQKSVQLENDSLMFDKWLKLPMPLDFKVYVFNVTNVEDVNKGEKPILEEIGPYVYKQYRERTVLGYGPNDTIKYMLRKRFEFDAEASGGLTEDDEVTVIHFSYLAALLTVHDMMPSLVGVINKALEQFFPSLEDAFLRVKVRDLFFDGIYLSCDGDNAALGLVCGKIKGDLPPTMRMAEGSNGFYFSMFSHMNRSETGPYEMNRGRDNIYELGNIVSYKGQEIMPMWGDKYCGQINGSDSSIFAPINEANVPQKLYTFEPDICRSLYVDLVEKRELFNISAYYYEISESALAAKSANHDNKCFCRKNWSANHDGCLLMGLLNLMPCQGAPAIASLPHFYLGSEELLEFFQSGIAPQREKHNSHVYIDPTTGVVLSGVKRLQFNIELRKIDTIPQLSSVPTGLFPMLWLEEGATIPDSIQQELRDSHKLLGYVEVARWLLLTVAVIAVIASAVAVARANALLSWPRNSNSVSFILGPSVTQVNKGN

>MsCSP1 c28555_g2

MKSYIALLVLSVAAMALARPEEAKYTDRYDNVNLDEVLSNRRLLVPYVKCILDQGKCAPDGKELKEHIKEALENECGKCTETQKSGTRRVIGHLINHEDAYWRELTAKYDPQRKFTAKYEKELKEIKQ

>MsCSP2 c25454_g1/g2

MQIKYALVLCCVAAVSLAQTQRPAVSDTADEALVLQGVVEQRQLKCALGEAPCDPIGKRLKTLAPLVLRGACPQCTPQETKQIQRTLSYVQRNFPQQWAKIVRQYAG

>MsCSP3 c28555_g3

MNSLTVLCLFALVALAVARPDGKYTDRYDSVNLDQILSNRRLLVPYIKCMLDQGKCTPDGKELKTHIREALEQDCAKCTKAQRDGTRQVMGHLINHEVDYWNELKAKYDPKNLYSTKHEQELRKLKQ

>MsCSP4 c25813_g1

MKFVLLLCVMVAAVVADDKYTDKYDNIDLDEILSNKRLLDAHYKCVMDKGKCTAEGKELKDHLTEAIENGCAKCTENQEKGAQKVIDHLIKNELDMWRELAAKYDPTGNWRKKYEDRARAAGIVIPAE

>MsCSP5 c26967_g1

MKADCVLLATLMAVVAADFYSSKYDSFDVQPLLENDRILLSYTKCFLDEGPCTPDAKDFKKVIPEALETTCGKCSPKQKQLIRMVVKAVIERHPEAWQQLSDKFDKDRKFKDSFDKFLAEED

>MsCSP6 c30240_g1

MVNLFLKMRAVFVLCVLVYVVVGQELNDMGNMPKYDSRYDYLDVDAIFTNKRLVRNYVDCLINSVRCSPEGKALKRILPEALRTKCVRCTERQKRAAVKVIRRLKNDFPEEWSKLASRWDPT

>MsCSP7 c17971_g1

MKTLFILCALVIAVSARPEEQYTTEYDNIDIDEILNNDRLFKSYFECLVGEGKCTPAGKELKSHMPDALQTECSKCSPKQKEGTKKVMKFLINNKPEQWKRLCAKYDPEGKYASKYEKELKEVSQ

>MsCSP8 c25444_g1

MRVLIVLSCLVVLAFAAEKYNAKYDNFDVETLISNDRLLKAYINCFLDKGRCTPEGSDFKKTLPEAIETTCAKCTEKQKGNIRKVIKAIQQKHPKEWDDLVKKNDPSGKNRANFDKFIQGSR

>MsCSP9 c27600_g1

MKLIVAVALLCVVAMAWGKPASTYTDKWDNINVDEILESQRLLKAYVDCLMDRGRCTPDGKALKETLPDALENECSKCTEKQKSGSDKVIRHLVNKRPDLWKELSTKYDPDNIYQDKYKTQIESAKQ

>MsCSP10 c27994_g1

MKTILVLCVLIAAVCARPEATYDTRYDNFDVESLVENVRLLKSYGHCFLGTGPCTPEGSAFKKTIPDALQTGCGKCSPRQRHLIRVVVNGFQTKTPDIWKQLVKKEDPNGEFKETFTRFLKASD

>MsCSP11 c3142_g1

LVAVAITLACVQAAEDKYSSKYDNINLDEVLGNKRLLNGYMKCTLDQGPCTAEGKELKYYISDGLKTGCSKCTPRQRKGVKKVMKY

>MsCSP12 c3874_g1

MKTWLLCLCVLTVVVSCYSQGPNRYENFNADAIIQNDRILLAYYKCVMDKGPCTRDGKNFKRVLPETLATACGRCNPAQKTIVRKLLLGIRTKSE

>MsCSP13 c21517_g1

MKLLIVLALVAAALARPDDSHYDEKYDNFNIDEVITNERLLKNYAHCLIGDGKCTPEGNEFKKLLPEATKSNCGKCTDKQKVHVAKAIKAIKEKLPTEYETLRSQIDPEGAHAEDINKYVAKYAP

>MsCSP14 c45234_g1

MQIIILTALCVGLVAGLHVQAGPQMTDAQLEQTLADKNTMQRHIKCALGEGPCDPVGRRLRTLAPLVLRGACPQCSMQETRQIRRTLAFVQRNYPWEWAKIVRQYG

>MsCSP15 c25484_g1

MKVLIVLTALVAFAAAAALTPEELKMLEAFDFDALFANDEQRKIVFDCMLDKGDCGPYKQLVELSTKTVTTKCADCSPAQKTKYDYVLKVLHDKYEPVYTEFLKKANAKKE

>MsCSP16 c25284_g1

MKVVLLTLCLALGVLAQDKYESANDDFDVSEVLSNPRLLNSYSKCLLNQGPCTPEVKQVKEKLPEALETRCAKCTDKQKQMGKALAQEVKKNHPDIWKQLVAMYDPQGKYQQAWQDFLKE

>MsCSP17 c24984_g1

MNFLVLSVVVTLAAFAAADLTYTDRYDHVNVDEILDNRKLLVPYIKCTLDQGRCTPDGKELKAHIKDAMQTGCAKCTKKQKKAAKKVVKHIRAKEQDYWKQIVNKYDPGNEYTETYEAFLASPDESK

>MsCSP18 c27178_g2

MMMLYSSLAMMLLTYLTIQSNATETSTYTTKYDGIDLDEILNNERLLTGYVNCLMDNGPCTADGKELKKNIPDAIENDCKKCTDRQRDGSDRVMHYLIDHRPDDWVKLEEKYNSDGSYKMKYLSSKKTEDSKETNGTKSEEETKNSSKE

>MsGOBP1 c27221_g2/g1

MTPTTLVLALGLAAALADVNVMKDVTLGFGQALDKCRQESDLTEEKMEEFFHFWRDDFKFEHRELGCAIQCMSRHFNLLTDSSRMHHDNTEQFIQAFPNGEVLARQMVSLIHGCEKQFDHEEDHCWRILHVAECFKQACVQHGVAPTMEMMMTEFIMEAEAR

>MsGOBP2 22940_g1

MTSKCGLLLAVMAAVAGSVMGTAEVMSHVTAHFGKALEECREESGLSAEILEEFQHFWREDFEVVHRELGCAIICMSNKFSLLQDDSRMHHVNMHDYVKSFPNGEILSGKLVELIHNCEKKFDSMTDDCDRVVKVAACFKVDAKAAGIAPEVAMIEAVMEKY

>MsPBP1 c26255_g1

MVLHRSATMSARLALVVIASLFIAVECSQEIMKNLAINFAKPLEDCRKEMDLPDSVLTDFNNFWKEGYEFTNRQTGCAILCLSSKLELLDPEMKLHHGRAQEFAQKHGADEAMAKQLVDMLHSCMQTTPDDANDPCLKTLKVVTCFKTKIHELKWAPSMDLIVGEVLAEV

>MsPBP2 c23411_g1

MADSRMRLACLVCVIFVASSAMASKELLTKMSSGFTKVVDQCKNELNVGEHIMQDMYNFWREEYALVNRDLGCMVMCMAAKLDLIGDDQKMHHGKAEEFAKSHGADDALAKQLVGLIHECETTHAGVEDACSRTLEVAKCFRTKIHELKW

>MsPBP3 c18728_g1

MGRNCIFFALVLMAVGVKEIAPSKDAMKYITSGFVKVLEECKHELNMNDQIMADLYHFWKLDYALLSRDTGCAIICMSKKLDLMDASGRMHHGNAQEFALKHGAGDDVASKIVTIIHDCEKKFERDDDECLRVLEVAKCFR

>MsOBP1 c23590_g1

MSKFTCLVFFIVAASISKAYASEEEKAAFREAVKPIIEECSKEHGVGIDELKAAKAAASADGIDNCFLGCVFKKAEVINAKGEFDLDNALTKLKGFVSNEDHFAKFEDIGKKCASVNEKPVSDGDAGCERAALLTACFLEHKGEMPLNF

>MsOBP2 c23590_g2

MSKFTCLVLCVVAVSISRAYASEEDKAAFRAAIQPIVDECSKEHGVSSDDIESAKTAGSADNIKPCFLGCVLKKAEILNAKGEYDSDKALTKLKKFVPDETKYAKYAEIGKKCESVNEKAVSDGEAGCERGALLTACFLENRADIL

>MsOBP3 20537_g1

MSKFTCIVFCIVAASLTKVSHAVTEEEKAAFREVMAPIIDECSGEHGVSKADIQAAKEAGSADGIKPCFLGCVMKKTETLDDKGLFDAETALSKLRTFVKSDEDFAKFEEIGKACMSVNEKSVSDGEAGCERAKLVLACFLEHKADIPF

>MsOBP4 c10494_g1

MFKLAYLLFCAVAVSLSGIVRAGEEDAEAFREAIKPFITECAKEHGISWEDIAKAKETHTVSSLKPCFVGCIFKKFEIINDKGEYDLEANLDKIKIFVKNEDLLTQLRDIMKKCVSVNDESVSDGNAGCERAMLLAKCFAELKSEILI

>MsOBP5 c20205_g1

MSKFSCLAFCVVVVSLNSVLAEDGPANEGDVLDIVFECAKENEVKASEILAVMTSRDVTLVNPCLWSCCLKKGGFIDDKGQYVLNPGLTYVKNIVKSDQFYTFIEKSAKQCESVKDKAGSECELGALLAACIVEQMMKM

>MsOBP6 c25084_g1

MSEFMSVLCAIVMSLNSVYTDAPLGLGDINTILLDCAKENKVTNDQLRTVVTSQDTKSVNSCFFACLFKRSKIMNDKGEFDVKTGLTYVRQVLPGRPETVVAESIIKECESVKNTAVNDGEAGCERAALLVACLLEQTTKKARKPK

>MsOBP7 26404_g1

MYKFTCFVFYILYAVFTQAESDSSDSGSDEVFDKLSHECMEKFGVTEDDLNGVVKTSDVTNIDSCYWGCYFTKMGVLNDKGQFDLNNFQTTMKKLMKDDEDYDNLEKLVKKCEPVKDETVTDGEAGCERGTLFAVCFVKNDGDFI

>MsOBP8 c21768_g1

MFKSTSLILYAVAVSLSNANDDGSKEFVSMVDECARLNGHTMSELSEVMSNGDVSVMKPCFWGCAFTKTGFLNDKGQYDVDSGLIGVKKYMKDPLGLEKLEQMARQCESVNDKVVSDGNAGCERGMLAAKCFLEKDNGQIVPSAL

>MsOBP9 c24094_g1

MFNVYFCVFVCGVLSLNIKASSLDDLKLKYVEVIIECSNDYPITVADMTELRKKIMPDSEPIRCLFACVYKKTGMMNEKGELSVDGVNEMSRKYLA

>MsOBP10 c23134_g1

MIKFSVVCLYFVVVAVHFWNVKCMTKDQEQEIIKAMKPLAEECASYCGLKDEDLKKYQGGDDMNPCFKKCMMQKLGLLDQEGKYDKATLHETMSQYGEDKEKAQKIEDQIDSCFMANADNNGDDEEAIKKRVDVMFNCIKELKE

>MsOBP11 c23549_g1

MNTSNFQSIFCIICIVSLFFSYSHAMTRQQLKNSGKLMKKSCMPKNDVTEDEVGDIEKGKFIENRNVMCYVACIYTMTQVVKNNKLSYEAVIKQVDIMFPAEMRDAVKAAATFCKDTTKKYKDLCEASYWTAKCMYDYDAENFVFP

>MsOBP12 c24343_g1

MDRKGLCLLIVAMFLATGSDAMSRQQLKNSGKVLKKNCMNKNQVTEDQIGTIDKGNFVEDKKVMCYIACIFEMTNVIKNGKLNYDASIRQIDLMYPPDLKEGAKAAVDKCKDVQKKYKDICEASFYVAKCMYEFNPADFIFA

>MsOBP13 c25075_g1

MLYSGSVFLLSFMLIMLNSSFISAMTREQVKSSGKMIKKTCSVKNNLSEDQVKDVDKGNFIEEKNFMCYVACVYKMGQTIKGNTINHDMMLKQVEMMFPTEMKAPVKAAIEHCRPVAKKYKDVCEAAYWTAKCTYEFDPANFMFP

>MsOBP14 c23825_g1

MNQSYLLLLIAACVEISYGMTRAQVKKTMTIIKNQCMPKNSVTEDQVKNIEQGDFNEDPNIMCYVACVYKSLQVVKNDKLDVGLISKQIDALYPPELKEPTKKAVALCINSQDNYNDLCSRVFHGAKCLYEKDPACFIFP

>MsOBP15 24885_g1

MLLIEIVKFLILVAMCEAMTMKQIRNTGKMMRKSCQPKNNVEDDKIDPIAEGVFIDEKEVKCYMACIMKMANTIKNGKLNFDAAIKQADLLLPDEVKEPAKEAILACKKAADGHKDICDASFHVTKCIYNQNPGIFYFP

>MsOBP16 c21911_g1

MFTGTLPVVLCLVAAAYGGKEKPVFSDEIKEIIQTVHDECVGKTGVAEEDITNCENGIFKEDTKLKCYMFCLMEEASLVDDDGTVDYDMLVSLIPDEYYERTTKMIFSCKHLDTPDKDKCQRAFEVHRCSYGKDPDLYFLF

>MsOBP17 c17143_g1

MTMWFRALAMLVAGLAAAQAIEMDEDMAELARMVRESCAAETGADVALVEQVNAGADLMPDAKLACYMKCTMETAGMMSDGEVDIEAVLALLPPELAAHKAPSLRACGTVHGADHCDTAWKTQKCWQAANKADYFLI

>MsOBP18 c29119_g1

MKSFVVFCLVLVVGVYANVTLPPTQQEKAQKLAAECVKESGVSTEVLAEAKKGHIVEDENLKKFTFCFFKKAGIVDSDGKLNVEVATAKLPPGVDKEDAKKVLEGCKSKTGKDTADTVFEIFKCYHKGTKTHILLAGL

>MsOBP19 c25074_g1

MKTFLVLAACILLAQGLTDEQKEKLKKHNTECLAETKVDEALVNKLKTGDYKTESEPLKKYALCMLMKSELMTKEGKFKKDVALAKVPNAADKPNVEKLIDSCLANKGNTPHQTAWNYVKCYHEKDPKHAIFV

>MsOBP20 c27420_g1

MKLFVVLCIVLVTEIYAAYVPLPPDHTDSNLEECRKTSEFTDDNLNKMKTNPFVEDGGEIFKKFIKCYLEKTGAITEDGKLNVDEALPKLGPNFAKKIFEHCKTHVETKGEEFVVVPTTTASDYSECFRQGVSNYIWNAKQEGFEPFTYEWQK

>MsOBP21 c25764_g1

MTKVLLATVLIVITFALTRAASTQMKDAMPKEPMTTTTMANQDSSIDSTDIDVIAVMNACNESFRIEMSYIQAMNESGSFLDETDKTPKCFIRCVFTNVGIVSEDGKQFNPARAAFIFAGERNGKPMDDIGDMTAACAADRQETCPCERSYQFLRCLMSMEIEKYEKS

>MsOBP22 c26026_g1

MFKFCVFLAFCVAASYGAPGGGTYCGETPSVIYQCLNSPKVISAVPAKCAKYDDECERLTCVFRESKWLDGTAVDKAKVLAHLDQYERDHAEWGPAVQFAKTACLGPELKAQGVFLNCPAYDVTHCILSSFIKHATPTQWSSSASCSYPHAYAAACPVCPSDCFSAQVPIGSCNACYLQPRTP

>MsOBP23 c23916_g1

MFKWLIIVALVAASYGDPISESRDNKSATLKPLSVCCDIPELGDPKHLAKCSNPKLPGPCNDIQCVFEESGFLTDVNTLNKEAYKNHLKQWEENHAGWSVAVDKAIKDCVDSDPRQHLNYPCKAYDVFTCTGIAMLKKCPAAAWKC

>MsOBP24 c25874_g1

MIRSCLVLAAVFQVLFGQESGPDPRDGFRQPVPHYCLSPPPGTDLHKCCPIPKLFPDGDMERCGIEKASVDQSKNPPKPRIPCKESICLMQNANMLLANHSVDYEKLRTFVDIWADSNPEFTEAILEAKKACAKDGGPSGPPVCEQDRIFYCLTSNVLWNCKLRDFEDCRVLKAHMDECRPYYWKKREEDEANAPTS

>MsOBP25 c26853_g1

MCLINYHVLILCLILVESYALNCRSSGGPKEAELKNIYKKCLKMQEGKNSSRGNSEQDYKEPRGQIQRSDWERGRTTGSKENKNGRDDRMSGKDRKGGSSMRDRDDMMGRTDDRMDRNDDRNNRNDDRMSSNNDRSGGRGRMGGNNNRNDMSRGRDDRFGNYNGKEDFPQSNEYGGHEMPGQGQYNNYYSTTPAPRRYKRERRPENSGQRSQYNPNNHKITGYEDSFRSDEKNTTENSSKETDNNACALHCFLENLEMTAEDGMPDRYLVTHAITKDVKDEDLRDFLQESIEECFQILDNENTEDKCEFSKNLLICLSEKGRANCDDWKDDLKF

>MsOBP26 c29915_g1

MVRKIGGLLCCLCVFGISLSDSAISADSESRCRNPPTAPQKIERVITLCQDEIKLSILREALDVIKEEHTMPAQRRRDKREVPFTHDEKRIAGCLLQCVYRKVKAVDGYGFPTLEGLVGLYSDGVNERGYFMAVLEASRECLMKNHDKFSRTVPMDNGRNCDISFDIFECISDRIGEYCGTAGL

>MsOBP27 c24197_g1

MYRLVILSIVAVSALADEMGMRECGRMFHPHSVRCCKKTSELKDKFMLSEDLKECFQMRGNPVTCENEVCIAKKKGFATDDDKLDYTKLEEVMTKEIDDKDLLADMIKNCVNGDLEKYGPPDFCEFMKMRHCISMQMLNHCPDWDDAGECSKLKGAVADCVKLFA

>MsOBP28 c27900_g1

MVGVVVPTVLLALLPAWVASSGEGNIKLLENEVAIALKACTYPEETTASKDGTSKERQRRSDDYDGSPRIDNNMKEGNRYSHERRNNDSGDQMMVLNATDYDYEGYGTGNMGEKLLTSIPRPASPNLHNNINNNNTSRTRRSEPLLNKPDSDQCLSQCVFANLQVVDSRGIPREAELWNKVQSAVTSQQSRSALHDQIRACFEELQSEAEDNGCSYSNKLERCLMLRFSDRQVDGKATTKKPTSTEQS

>c22587_g1

MFVKNLAVLFTLTLVTFCNSSFIEDLPKCHLEDFDCLKDVYQTMIRKMGKEGLKEFNIPPLDPMKLTNVNVNVHNLVNVTMVDGIVKGIRDCVFDAFSINIKEGRGHQENTCDLVIKGHYTAEASSDLIASLLGGSSIHGDGHAKVTVGQVHVKFDFPFYAQKRDDGEIYIKCNYDLVKYDYDIGGPFVIKADNLYLGDKESSKLITDMINQNWRILMTGFGQPFITKAIEEFYFKWSGNFFDKVAARHFIIEDLSPYARP

>c30753_g1

MFGSKTVFYLLTVFSACFGAVDIKKYLKVCDRNAIDVNDCMADAVQKGIAVMIHGIPELGVPPIDPYLQKEFRVEYKNNQILAKMILKNIYVEGLKEAKVHDARLRADDDKFHLEVDLTSPMVAVKAQYYGEGQFNSLKIVAYGDFNTTMTDLVYTWKLSGVTEKNGTETYVRIKDFYMRPDLASIVTEFRNENPESREFTDLGTRFANENWQTLYKEFLPYAQANWKRIGIKVANKLFLKVPYDQLFPSSS

>c28009_g1

MKGFVFTVLVAIFGLAACNEEIHITACREEDAACLKASAQAAVPLLAAGVPSMGIAVMDPMHVEQVKTTQAGLAMDFRNTSVTGLRHCKVLDLKRHHHLTNLDLKCSVVMRGDYTLGGKLLIMPIEGSGRYSIKIHDIVVKIQFHVDEVPRDGVTYWIVKSWKFTTDVQKGVHFMFKNLFNGNKQLSDAVHQFANSNWKDIFQEVAPPIVKVIVSNVVVESTKLFDKVPLNKLVIQ

>c27577_g1

MYSRYSFLVLALSVVYSNAKLAPFIQKCKAEDSKCLKETAQNAIPIFANGIPELGVQKLDPFTMKSLDASTPGLKLKLWDITGTGLKDCVAKKVQRDEGKSKITVKLQCSVDFKGKYDMSGQLLVLPIQGNGNAHVVLNKVVILADVDLSDNIGKDGEKHWTIKSWKHSYDLKDKSTIELENLFNGNEVLGRAARELIASSSNEIVKEVGPPIVKAIIAKIIENIENFFKHVPASELE

>c29527_g1

"MNNKVFILVFLTYISLAVASKAPFITKCKANDDKCHTESAQKVIPLFADGIPELNVEKHDPLILKYVDASTSNLKLIVTDIVVKGLKNCVAKKISRGDLKLVVKIQCAVDFKGKYDMNGQLFLLPIAGSGDLTAYVPSILIEVLADVKEKTGKDGKMHWAVKSWSHTFELKEKSDVKFENLFPDNELLRKTTEELIAKNGNDVIIEIGKEIIKALCGKAIEGINKFFLAVPYEDLTL

**Supplementary Table 4.**

| Gene | Accession number | Description |
| --- | --- | --- |
| **Clock genes** | | |
| MsCRY2 | AFR54427 | cryptochrome 2 [Mythimna separata] |
| BmCRY1 | NP_001182628 | cryptochrome 1 [Bombyx mori] |
| BmCRY2 | NP_001182627 | cryptochrome 2 [Bombyx mori] |
| BmPER | ABF21088 | period [Bombyx mori] |
| BmCLK | XP_012549005 | circadian locomoter output cycles protein kaput [Bombyx mori] |
| BmCYC | NP_001036982 | Cycle like factor b [Bombyx mori] |
| BmVRI | XP_004923610 | PREDICTED: nuclear factor interleukin-3-regulated protein [Bombyx mori] |
| BmTIM | NP_001037622 | timeless [Bombyx mori] |
| BmSGG | XP_004928285 | PREDICTED: glycogen synthase kinase-3 beta isoform X5 [Bombyx mori] |
| BmPDP1 | XP_004924931 | PREDICTED: hepatic leukemia factor isoform X11 [Bombyx mori] |
| BmCK2a | NP_001036956 | casein kinase 2 alpha subunit [Bombyx mori] |
| BmCK2b | NP_001036989 | casein kinase 2 beta subunit [Bombyx mori] |
| BmMET | BAJ05086 | methoprene-tolerant homolog-2 [Bombyx mori] |
| BmSLMB | XP_012545004 | PREDICTED: beta-TrCP isoform X2 [Bombyx mori] |
| BmDBT | NP_001037285 | double-time protein [Bombyx mori] |
| DmCRYd | BAA35000 | blue light photoreceptor [Drosophila melanogaster] |
| DmPHR6-4 | NP_477188 | (6-4)-photolyase, isoform B [Drosophila melanogaster] |
| DmPER | CAA27285 | per protein [Drosophila melanogaster] |
| DmCLK | AAD10630 | CLOCK [Drosophila melanogaster] |
| DmCYC | NP_524168 | cycle [Drosophila melanogaster] |
| DmVRI | NP_723075 | vrille, isoform C [Drosophila melanogaster] |
| DmTIM | AAC46920 | TIM [Drosophila melanogaster] |
| DmSGG | NP_476714 | shaggy, isoform A [Drosophila melanogaster] |
| DmPDP1 | NP_001261546 | PAR-domain protein 1, isoform N [Drosophila melanogaster] |
| DmCK2a | NP_524918 | casein kinase IIalpha, isoform B [Drosophila melanogaster] |
| DmCK2b | NP_542940 | casein kinase II beta subunit, isoform B [Drosophila melanogaster] |
| DmMET | NP_511126 | Methoprene-tolerant, isoform A [Drosophila melanogaster] |
| DmSLMB | AAC38852 | Slimb [Drosophila melanogaster |
| DmDBT | AAC39134 | casein kinase I homolog [Drosophila melanogaster] |
|  |  |  |
| **Melanin synthesis genes** | | |
| MsPPO1 | BAM76811 | prophenoloxidase 1 [Mythimna separata] |
| BmPPO1 | AAG09304 | prophenoloxidase-1 [Bombyx mori] |
| BmPPO2 | AAG09303 | prophenoloxidase-2 [Bombyx mori] |
| DmPPO2 | NP_610443 | prophenoloxidase 2 [Drosophila melanogaster] |
| Bmtan | NP_001170882 | tan protein [Bombyx mori] |
| Dmtan | NP_572543 | tan, isoform A [Drosophila melanogaster] |
| BmaaNAT | NP_001073122 | arylalkylamine N-acetyltransferase [Bombyx mori] |
| DmaaNAT | NP_523839 | dopamine N acetyltransferase, isoform A [Drosophila melanogaster] |
| BmDDC | NP_001037174 | aromatic-L-amino-acid decarboxylase [Bombyx mori] |
| DmDDC | AAF53763 | dopa decarboxylase, isoform B [Drosophila melanogaster] |
| BmLACx | [XP_012552135](https://www.ncbi.nlm.nih.gov/protein/827563025?report=genbank&log$=protalign&blast_rank=4&RID=4CRJDNXK014) | PREDICTED: L-ascorbate oxidase isoform X1 [Bombyx mori] |
| DmLAC1 | NP_609287 | multicopper oxidase-1, isoform A [Drosophila melanogaster] |
| BmLAC2 | XP_004930412 | PREDICTED: laccase-2-like [Bombyx mori] |
| DmLAC2 | NP_001137606 | laccase 2, isoform D [Drosophila melanogaster] |
| BmTH | NP_001138794 | tyrosine hydroxylase [Bombyx mori] |
| DmTH | NP_476898 | pale, isoform B [Drosophila melanogaster] |
| Bmebony | BAH11147 | ebony protein [Bombyx mori] |
| Dmebony | ABO27243 | ebony [Drosophila melanogaster] |
| BmGTPCH | NP_001138797 | GTP cyclohydrolase I isoform B [Bombyx mori] |
| DmGTPCH | AAR20855 | punch [Drosophila melanogaster] |
|  |  |  |
| **Yellow family genes** | | |
| Dmyellow-b | NP_523586 | yellow-b, isoform A [Drosophila melanogaster] |
| Dmyellow-c | NP_523570 | yellow-c, isoform A [Drosophila melanogaster] |
| Dmyellow-f | NP_524335 | yellow-f, isoform A [Drosophila melanogaster] |
| Dmyellow | NP_476792 | yellow [Drosophila melanogaster] |
| Dmyellow-h | NP_651912 | yellow-h [Drosophila melanogaster] |
| Dmyellow-e3 | NP_650288 | yellow-e3 [Drosophila melanogaster] |
| Dmyellow-d | NP_523820 | yellow-d [Drosophila melanogaster] |
| Dmyellow-e | NP_524344 | yellow-e [Drosophila melanogaster] |
| Bmyellow-x | NP_001037430 | yellow-b precursor [Bombyx mori] |
| Bmyellow-e | BAI39592 | Yellow-e protein [Bombyx mori] |
| Bmyellow-d | ABC96694 | yellow-d [Bombyx mori] |
| Bmyellow | BAH11146 | yellow protein [Bombyx mori] |
| Bmyellow-f | NP_001037424 | yellow-fa precursor [Bombyx mori] |
| Bmyellow-c | ABC96696 | yellow-c [Bombyx mori] |
| Bmyellow-b | BGIBMGA014224-PA [SilkDB] |  |
| Bmyellow-h2 | BGIBMGA007255-PA [SilkDB] |  |
|  |  |  |
| **Sensory neuron membrane protein** | | |
| BmSNMP1 | NP_001037186 | sensory neuron membrane protein 1 [Bombyx mori] |
| SeSNMP1 | AGN52676 | sensory neuron membrane protein 1 [Spodoptera exigua] |
| DkSNMP1 | AII01132 | SNMP [Dendrolimus kikuchii] |
| SeSNMP2 | AGN52677 | sensory neuron membrane protein 2 [Spodoptera exigua] |
| DkSNMP2 | AII01133 | SNMP2 [Dendrolimus kikuchii] |
| BmSNMP2 | XP_012547405 | sensory neuron membrane protein 2-like [Bombyx mori] |
|  |  |  |
| **Chemosensory protein** | | |
| BmCSP1 | ABH88194 | chemosensory protein 1 [Bombyx mori] |
| BmCSP2 | ABH88195 | chemosensory protein 2 [Bombyx mori] |
| BmCSP3 | ABH88196 | chemosensory protein 3 [Bombyx mori] |
| BmCSP4 | ABH88197 | chemosensory protein 4 [Bombyx mori] |
| BmCSP5 | ABH88198 | chemosensory protein 5 [Bombyx mori] |
| BmCSP6 | ABH88199 | chemosensory protein 6 [Bombyx mori] |
| BmCSP7 | ABH88200 | chemosensory protein 7 [Bombyx mori] |
| BmCSP8 | ABH88201 | chemosensory protein 8 [Bombyx mori] |
| BmCSP9 | ABH88202 | chemosensory protein 9 [Bombyx mori] |
| BmCSP10 | ABH88203 | chemosensory protein 10 [Bombyx mori] |
| BmCSP11 | ABH88204 | chemosensory protein 11 [Bombyx mori] |
| BmCSP12 | ABH88205 | chemosensory protein 12 [Bombyx mori] |
| BmCSP13 | ABH88206 | chemosensory protein 13 [Bombyx mori] |
| BmCSP14 | ABH88207 | chemosensory protein 14 [Bombyx mori] |
| BmCSP15 | ABH88208 | chemosensory protein 15 [Bombyx mori] |
| BmCSP16 | ABH88209 | chemosensory protein 16 [Bombyx mori] |
| DkCSP1 | AII01028 | chemosensory protein [Dendrolimus kikuchii] |
| DkCSP2 | AII01029 | chemosensory protein [Dendrolimus kikuchii] |
| DkCSP3 | AII01030 | chemosensory protein [Dendrolimus kikuchii] |
| DkCSP4 | AII01031 | chemosensory protein [Dendrolimus kikuchii] |
| DkCSP5 | AII01032 | chemosensory protein [Dendrolimus kikuchii] |
| DkCSP6 | AII01033 | chemosensory protein [Dendrolimus kikuchii] |
| DkCSP7 | AII01034 | chemosensory protein [Dendrolimus kikuchii] |
| DkCSP8 | AII01035 | chemosensory protein [Dendrolimus kikuchii] |
| DkCSP9 | AII01036 | chemosensory protein [Dendrolimus kikuchii] |
| DkCSP10 | AII01037 | chemosensory protein [Dendrolimus kikuchii] |
| DkCSP11 | AII01038 | chemosensory protein [Dendrolimus kikuchii] |
| DkCSP12 | AII01039 | chemosensory protein [Dendrolimus kikuchii] |
| DkCSP13 | AII01040 | chemosensory protein [Dendrolimus kikuchii] |
| DkCSP14 | AII01041 | chemosensory protein [Dendrolimus kikuchii] |
| DkCSP15 | AII01042 | chemosensory protein [Dendrolimus kikuchii] |
| DkCSP16 | AII01043 | chemosensory protein [Dendrolimus kikuchii] |
| DkCSP17 | AII01044 | chemosensory protein [Dendrolimus kikuchii] |
| SeCSP1 | ABM67688 | chemosensory protein CSP1 [Spodoptera exigua] |
| SeCSP2 | ABM67689 | chemosensory protein CSP2 [Spodoptera exigua] |
| SeCSP3 | ABM67690 | chemosensory protein CSP3 [Spodoptera exigua] |
| SeCSP4 | AKT26481 | chemosensory protein 4 [Spodoptera exigua] |
| SeCSP5 | AKT26482 | chemosensory protein 5 [Spodoptera exigua] |
| SeCSP6 | AKT26483 | chemosensory protein 6 [Spodoptera exigua] |
| SeCSP7 | AKT26484 | chemosensory protein 7 [Spodoptera exigua] |
| SeCSP8 | AKT26485 | chemosensory protein 8 [Spodoptera exigua] |
| SeCSP10 | AKT26486 | chemosensory protein 10 [Spodoptera exigua] |
| SeCSP11 | AKT26487 | chemosensory protein 11 [Spodoptera exigua] |
| SeCSP12 | AKT26488 | chemosensory protein 12 [Spodoptera exigua] |
| SeCSP13 | AKT26489 | chemosensory protein 13 [Spodoptera exigua] |
| SeCSP14 | AKT26490 | chemosensory protein 14 [Spodoptera exigua] |
| SeCSP16 | AKT26491 | chemosensory protein 16 [Spodoptera exigua] |
| SeCSP18 | AKT26492 | chemosensory protein 18 [Spodoptera exigua] |
| SeCSP19 | AKT26493 | chemosensory protein 19 [Spodoptera exigua] |
| SeCSP20 | AKT26494 | chemosensory protein 20 [Spodoptera exigua] |
|  |  |  |
| **Odorant binding protein** | | |
| DkGOBP1 | AGJ83357 | general odorant-binding protein 1 [Dendrolimus kikuchii] |
| DkGOBP2 | AGJ83353 | general odorant-binding protein 2 [Dendrolimus kikuchii] |
| DkPBP1 | AGJ83349 | pheromone binding protein 1 [Dendrolimus kikuchii] |
| DkPBP2 | AII01010 | odorant binding protein [Dendrolimus kikuchii] |
| DkOBP1 | AII00987 | odorant binding protein [Dendrolimus kikuchii] |
| DkOBP2 | AII00988 | odorant binding protein [Dendrolimus kikuchii] |
| DkOBP3 | AII00989 | odorant binding protein [Dendrolimus kikuchii] |
| DkOBP4 | AII00990 | odorant binding protein [Dendrolimus kikuchii] |
| DkOBP5 | AII00991 | odorant binding protein [Dendrolimus kikuchii] |
| DkOBP6 | AII00992 | odorant binding protein [Dendrolimus kikuchii] |
| DkOBP7 | AII00993 | odorant binding protein [Dendrolimus kikuchii] |
| DkOBP8 | AII00994 | odorant binding protein [Dendrolimus kikuchii] |
| DkOBP9 | AII00995 | odorant binding protein [Dendrolimus kikuchii] |
| DkOBP10 | AII00996 | odorant binding protein [Dendrolimus kikuchii] |
| DkOBP11 | AII00997 | odorant binding protein [Dendrolimus kikuchii] |
| DkOBP12 | AII00998 | odorant binding protein [Dendrolimus kikuchii] |
| DkOBP13 | AII00999 | odorant binding protein [Dendrolimus kikuchii] |
| DkOBP14 | AII01000 | odorant binding protein [Dendrolimus kikuchii] |
| DkOBP15 | AII01001 | odorant binding protein [Dendrolimus kikuchii] |
| DkOBP16 | AII01002 | odorant binding protein [Dendrolimus kikuchii] |
| DkOBP17 | AII01003 | odorant binding protein [Dendrolimus kikuchii] |
| DkOBP18 | AII01004 | odorant binding protein [Dendrolimus kikuchii] |
| DkOBP19 | AII01005 | odorant binding protein [Dendrolimus kikuchii] |
| DkOBP20 | AII01006 | odorant binding protein [Dendrolimus kikuchii] |
| DkOBP21 | AII01007 | odorant binding protein [Dendrolimus kikuchii] |
| DkOBP22 | AII01008 | odorant binding protein [Dendrolimus kikuchii] |
| DkOBP23 | AII01009 | odorant binding protein [Dendrolimus kikuchii] |
| SeGOBP1 | AHI16728 | general odorant-binding protein 1 [Spodoptera exigua] |
| SeGOBP2 | AGH70098 | general odorant-binding protein 2 [Spodoptera exigua] |
| SePBP1 | AAS46620 | pheromone binding protein 1 [Spodoptera exigua] |
| SePBP2 | AAS55551 | pheromone binding protein 2 [Spodoptera exigua] |
| SePBP3 | AHI16727 | pheromone binding protein 3 [Spodoptera exigua] |
| SeOBP1 | ADY17884 | odorant binding protein [Spodoptera exigua] |
| SeOBP2 | ADY17885 | odorant binding protein [Spodoptera exigua] |
| SeOBP3 | ADY17886 | odorant binding protein [Spodoptera exigua] |
| SeOBP4 | AFM77983 | odorant binding protein [Spodoptera exigua] |
| SeOBP5 | AFM77984 | odorant binding protein [Spodoptera exigua] |
| SeOBP6 | ADY17882 | odorant binding protein [Spodoptera exigua] |
| SeOBP7 | AGH70103 | odorant binding protein [Spodoptera exigua] |
| SeOBP8 | AGP03454 | odorant binding protein [Spodoptera exigua] |
| SeOBP9 | AGP03455 | odorant binding protein [Spodoptera exigua] |
| SeOBP10 | AGP03456 | odorant binding protein [Spodoptera exigua] |
| SeOBP11 | AGP03457 | odorant binding protein [Spodoptera exigua] |
| SeOBP12 | AGP03458 | odorant binding protein [Spodoptera exigua] |
| SeOBP13 | AGP03459 | odorant binding protein [Spodoptera exigua] |
| SeOBP14 | AGP03460 | odorant binding protein [Spodoptera exigua] |
| SeOBP15 | AGH70102 | odorant binding protein [Spodoptera exigua] |
| SeOBP16 | AGH70104 | odorant binding protein [Spodoptera exigua] |
| SeOBP17 | AKT26495 | odorant binding protein [Spodoptera exigua] |
| SeOBP18 | AKT26496 | odorant binding protein [Spodoptera exigua] |
| SeOBP19 | AKT26497 | odorant binding protein [Spodoptera exigua] |
| SeOBP20 | AKT26498 | odorant binding protein [Spodoptera exigua] |
| SeOBP21 | AGH70105 | odorant binding protein [Spodoptera exigua] |
| SeOBP22 | AKT26499 | odorant binding protein [Spodoptera exigua] |
| SeOBP24 | AKT26501 | odorant binding protein [Spodoptera exigua] |
| SeOBP26 | AKT26503 | odorant binding protein [Spodoptera exigua] |
| SeOBP27 | AKT26504 | odorant binding protein [Spodoptera exigua] |
| SeOBP28 | AGH70107 | odorant binding protein [Spodoptera exigua] |
| SeOBP29 | AGP03461 | odorant binding protein [Spodoptera exigua] |
| BmGOBP1 | BGIBMGA012611-PA [SilkDB] | GOBP1 [Bombyx mori] |
| BmGOBP2 | BGIBMGA012614-PA [SilkDB] | GOBP2 [Bombyx mori] |
| BmPBP1 | BGIBMGA012615-PA [SilkDB] | PBP1 [Bombyx mori] |
| BmPBP2 | BGIBMGA012616-PA [SilkDB] | PBP2 [Bombyx mori] |
| BmPBP3 | BGIBMGA012617-PA [SilkDB] | PBP3 [Bombyx mori] |
| BmOBP5 | Gong et al., 2009 | OBP5 [Bombyx mori] |
| BmOBP7 | BGIBMGA008356-PA [SilkDB] | OBP7 [Bombyx mori] |
| BmOBP8 | BGIBMGA008355-PA [SilkDB] | OBP8 [Bombyx mori] |
| BmOBP9 | Gong et al., 2009 | OBP9 [Bombyx mori] |
| BmOBP10 | Gong et al., 2009 | OBP10 [Bombyx mori] |
| BmOBP11 | NP_001140190 | OBP6 [Bombyx mori] |
| BmOBP12 | Gong et al., 2009 | OBP12 [Bombyx mori] |
| BmOBP13 | BGIBMGA008353-PA [SilkDB] | OBP13 [Bombyx mori] |
| BmOBP14 | Gong et al., 2009 | OBP14 [Bombyx mori] |
| BmOBP15 | BGIBMGA008352-PA [SilkDB] | OBP15 [Bombyx mori] |
| BmOBP16 | Gong et al., 2009 | OBP16 [Bombyx mori] |
| BmOBP17 | BGIBMGA008351-PA [SilkDB] | OBP17 [Bombyx mori] |
| BmOBP18 | BGIBMGA008474-PA [SilkDB] | OBP18 [Bombyx mori] |
| BmOBP19 | NP_001140188 | OBP19 [Bombyx mori] |
| BmOBP20 | BGIBMGA002308-PA [SilkDB] | OBP20 [Bombyx mori] |
| BmOBP21 | BGIBMGA003463-PA [SilkDB] | OBP21 [Bombyx mori] |
| BmOBP22 | BGIBMGA002630-PA [SilkDB] | OBP22 [Bombyx mori] |
| BmOBP23 | BGIBMGA002629-PA [SilkDB] | OBP23 [Bombyx mori] |
| BmOBP25 | BGIBMGA002627-PA [SilkDB] | OBP25 [Bombyx mori] |
| BmOBP26 | BGIBMGA002666-PA [SilkDB] | OBP26 [Bombyx mori] |
| BmOBP27 | BGIBMGA002626-PA [SilkDB] | OBP27 [Bombyx mori] |
| BmOBP28 | Gong et al., 2009 | OBP28 [Bombyx mori] |
| BmOBP29 | BGIBMGA010039-PA [SilkDB] | OBP29 [Bombyx mori] |
| BmOBP30 | BGIBMGA010011-PA [SilkDB] | OBP30 [Bombyx mori] |
| BmOBP31 | BGIBMGA010010-PA [SilkDB] | OBP31 [Bombyx mori] |
| BmOBP32 | BGIBMGA009290-PA [SilkDB] | OBP32 [Bombyx mori] |
| BmOBP33 | BGIBMGA009291-PA [SilkDB] | OBP33 [Bombyx mori] |
| BmOBP34 | BGIBMGA009292-PA [SilkDB] | OBP34 [Bombyx mori] |
| BmOBP35 | Gong et al., 2009 | OBP35 [Bombyx mori] |
| BmOBP36 | BGIBMGA009352-PA [SilkDB] | OBP36 [Bombyx mori] |
| BmOBP37 | BGIBMGA009366-PA [SilkDB] | OBP37 [Bombyx mori] |
| BmOBP38 | BGIBMGA013247-PA [SilkDB] | OBP38 [Bombyx mori] |
| BmOBP39 | BGIBMGA000225-PA [SilkDB] | OBP39 [Bombyx mori] |
| BmOBP40 | BGIBMGA011298-PA [SilkDB] | OBP40 [Bombyx mori] |
| BmOBP41 | BGIBMGA011276-PA [SilkDB] | OBP41 [Bombyx mori] |
| BmOBP42 | BGIBMGA011433-PA [SilkDB] | OBP42 [Bombyx mori] |
| BmOBP43 | BGIBMGA011432-PA [SilkDB] | OBP43 [Bombyx mori] |
| BmOBP44 | BGIBMGA010425-PA [SilkDB] | OBP44 [Bombyx mori] |
